# Diet and the Developing Brain: A Systematic Review of Nutritional Influences on Adolescent Cognitive and Academic Outcomes

**DOI:** 10.1016/j.advnut.2026.100648

**Published:** 2026-05-07

**Authors:** Hayley A Young, Chantelle M Gaylor, Anthony Brennan, Abigail McIntosh, Amy R Griffiths

**Affiliations:** School of Psychology, Faculty of Medicine, Health and Life Sciences, Swansea University, Wales, United Kingdom

**Keywords:** adolescence, diet, cognition, academic performance, infancy

## Abstract

Adolescence is a critical period of neurodevelopment, yet the role of nutrition in shaping cognitive and academic outcomes during this stage remains underexplored. This systematic review synthesizes evidence from 48 controlled trials and 25 prospective studies examining how diet influences cognitive performance and educational attainment between ages 8 and 19. Four databases (PsycINFO, Scopus, PubMed, Web of Science) were searched up to February 2026. Studies were synthesized by design, with risk of bias assessed using Cochrane and Joanna Briggs Institute tools. Prospective studies beginning in infancy—typically of higher methodological quality—suggest that unhealthy diets in the first 3 y of life may have lasting adverse effects on intelligence in adolescence. Controlled trials of dietary interventions during adolescence point to potential benefits for cognitive and academic outcomes, though findings vary and are often constrained by methodological limitations. To advance research in this field, we propose 7 guiding principles, including adopting a life course perspective, moving beyond nutrient isolation, using biologically valid biomarkers, incorporating puberty and sex-specific analyses, standardizing outcome measures, prioritizing context and population characteristics, and controlling for key confounders. These principles aim to strengthen the design, relevance, and impact of future studies in adolescent nutrition and brain health.

This study was preregistered on PROSPERO as CRD42023413970.

## Graphical abstract

**Figure:**
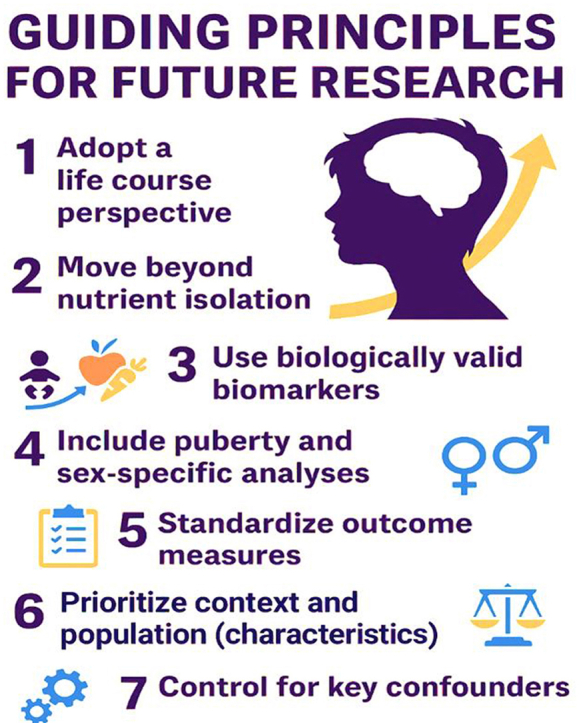

**Statement of Significance**

This review synthesizes evidence from controlled trials and prospective studies linking diet to adolescent cognitive and academic outcomes. It also introduces a 7-principle roadmap to guide more rigorous, policy-relevant, and developmentally informed research in adolescent brain health.

## Introduction

There is growing recognition that nutrition plays a foundational role in shaping neurocognitive development and lifelong brain health [1]. Historically, research in this area has concentrated on the effects of individual micronutrients during the highly sensitive neurodevelopmental window spanning mid-gestation to the first 2 y of life [2]. The significance of this period is underscored by global health estimates suggesting that the elimination of iron, zinc, and iodine deficiencies could result in a 10-point increase in mean global IQ [3]. However, far less is understood about how diet influences the brain and cognitive function during adolescence—a life-stage equally characterized by intense neurodevelopmental activity.

Adolescence constitutes a second window of neuroplasticity, marked by widespread structural and functional reorganization of the brain, driven in part by hormonal and endocrine shifts during puberty [4]. Neurobiological changes, including synaptic pruning, myelination, and the refinement of frontoparietal connectivity—are particularly pronounced in the prefrontal cortex, a region critical for executive functioning and higher-order cognition [5]. These neural changes underpin a cascade of emerging cognitive abilities which, in turn, are bidirectionally related to academic achievement and psychosocial development [6,7].

Crucially, this period of enhanced brain plasticity also confers heightened nutritional sensitivity. Adolescents face unique vulnerabilities to both overnutrition (e.g., diets high in ultraprocessed foods) and undernutrition (e.g., micronutrient insufficiencies), which may have enduring consequences for cognitive trajectories [8,9]. Despite this, the impact of diet on cognitive and academic outcomes during adolescence remains underexplored. A range of dietary interventions have been implemented in adolescent populations—including micronutrient supplementation [10,11], fatty fish meals [12], and school breakfast programs (SBPs) [13]—but their effects have yet to be synthesized in a unified framework.

To address this gap, the present systematic review aims to provide a comprehensive evaluation of the influence of diet on adolescent cognitive and academic outcomes. The review includes both randomized controlled trials (RCTs) and rigorously conducted non-RCTs that examine the effects of specific nutrients, whole diets, and broader dietary patterns. In recognition of the hierarchical nature of brain maturation—where later neurocognitive development is scaffolded on earlier developmental milestones [14]—the review also incorporates longitudinal studies, exploring associations between early-life diet (from birth to 3 y of age) and cognitive and academic performance during adolescence, as well as studies examining the impact of dietary patterns during early adolescence on outcomes measured later in adolescence.

In doing so, this review aims to advance theoretical and empirical understanding of how dietary exposures during key developmental stages contribute to neurocognitive function, learning, and educational attainment.

## Methods

The present review was conducted in accordance with the PRISMA guidelines [15]. PRISMA 2020 checklists are provided in Supplemental Document 1 (Supplemental Tables 1 and 2). A protocol was registered on the Open Science Framework registry (https://osf.io/c6xze) and PROSPERO (CRD42023413970). The systematic review was completed in 2 phases. During phase I, a literature search was conducted to determine the scope of literature using preliminary eligibility criteria (Supplemental Table 3). During phase II, the final research questions and eligibility criteria were implemented.

### Search strategy and selection criteria

A systematic search for studies published up to December 2024 was conducted using 4 databases: PsycINFO, Scopus, PubMed, and Web of Science. The search was restricted to English-language articles only, and both British and American spellings of key search terms were used. MeSH terms were applied. The following search terms were used: [Adolescents OR Adolescence] AND [Food OR Meal OR Diet OR Dietary pattern OR Nutrient] AND [Cognition OR Memory OR Attention OR Executive functioning OR Academic performance OR Brain functioning OR Brain structure OR Neuroimaging OR Functional magnetic resonance imaging OR fMRI OR Positron emission tomography OR PET]. The literature search was conducted twice—once using the term “infants” and once excluding this term. The purpose of this additional search was to determine the number of prospective studies that examined the impact of diet during infancy (<3 y) on cognitive or academic performance during adolescence. All 13 prospective studies that began in infancy were already captured in the primary search without the “infants” term; the additional search including this term did not identify any further eligible studies but confirmed that infancy-origin cohorts had been comprehensively retrieved. Reference lists from articles and reviews identified during the literature search were checked for relevant studies. Titles and abstracts were read to check for duplicates and to determine whether studies met the preliminary eligibility criteria listed in Supplemental Table 3 (Supplemental Document 1). Full texts were retrieved if abstracts did not provide sufficient information for evaluation. Studies that did not fulfil the preliminary eligibility criteria or were clearly irrelevant to the review were eliminated during phase I. The remaining articles were read in their entirety to establish their suitability during phase II. The systematic search was conducted independently by 2 authors (CMG and HAY). Any disagreements were resolved by discussion and where necessary involved a third reviewer (AM). To ensure that no eligible studies published during the review period were missed, we conducted an updated search of all databases on 16 February, 2026. This search identified several additional publications [[16], [17], [18], [19], [20], [21]], but these did not meet the a priori inclusion criteria (e.g., cross-sectional design only or absence of eligible adolescent cognitive or academic outcomes) and were therefore excluded.

This review forms part of a single registered project that originally aimed to synthesize associations between adolescent diet and a broad set of outcomes, including mental health and cognitive/academic performance. During phase I, scoping searches indicated that the breadth and heterogeneity of outcomes would be better addressed in separate but linked syntheses. The protocol was therefore prospectively updated in phase II, before application of the final eligibility criteria and data extraction, to specify that mental-health and cognitive/academic outcomes would be reported in 2 distinct reviews. A companion review focusing on adolescent mental-health outcomes has since been published [22], whereas the present manuscript reports the cognitive and academic outcomes.

### Eligibility criteria

The list of inclusion and exclusion criteria implemented during phase I can be found in Supplemental Table 3 (Supplemental Document 1). Briefly, studies examining the association between any aspect of diet and cognitive or academic performance during adolescence were eligible, including cross-sectional studies or acute (single consumption) or chronic (intervention period >1 mo) RCTs or non-RCTs. Prospective studies examining the association between: *1)* dietary intake during infancy and cognitive or academic performance during adolescence and *2)* dietary intake during early adolescence and cognitive or academic performance during late adolescence were also included. Studies involving adolescents from the general population or with pre-existing nutritional deficiencies were included, whereas adolescents with a mental or physical disorder were excluded.

In line with the WHO definition of adolescence [23], only studies with an age range between 10 and 19 y were eligible for inclusion during phase I. However, the preliminary literature search revealed that several studies recruited both children (<10 y) and adolescents, and few of these studies analyzed children and adolescents separately. To avoid excluding many potentially important studies from the present review, the age range was extended to 8–19 y during phase II. A total of 39 acute RCTs, 48 chronic RCTs/non-RCTs, 101 cross-sectional studies, and 25 prospective studies were identified as eligible during phase I. Because an unexpectedly large number of studies were identified, phase II focused on the most rigorous studies, specifically chronic RCTs/non-RCTs and prospective studies. PROSPERO was updated accordingly.

The final inclusion criteria implemented during phase II were as follows: *1)* chronic RCTs or non-RCTs that investigated the effect of any aspect of diet (excluding nutritive/nonnutritive sweeteners, breastfeeding, caffeine, or alcohol) on cognitive or academic performance in adolescents (8–19 y) from the general population or with pre-existing nutritional deficiencies, *2)* prospective studies that investigated the relationship between dietary intake during infancy and cognitive or academic performance during adolescence, and *3)* prospective studies that investigated the relationship between dietary intake during early adolescence and cognitive or academic performance during later adolescence. Studies that related changes in cognitive or academic performance to changes in biochemical measures (e.g., circulating nutrient levels) or brain functioning/morphology (e.g., MRI or functional MRI) were included.

### Data extraction

The following data were extracted by CMG: *1)* participant characteristics [sample size, sex distribution, mean age/age range, socioeconomic status (SES), and baseline nutritional status], *2)* year of publication, *3)* study characteristics (design, country of origin, and number of schools), *4)* dietary intervention (meal composition/timing and supplement dose/frequency/duration, if relevant), *5)* dietary intake (method of assessment and dietary patterns, if relevant), *6)* outcome measures, and *7)* details for quality appraisal.

### Outcome measures

The primary measure was cognitive performance, operationalized using validated and objective tests. Secondary outcome measures included standardized or unstandardized measures of academic performance. Additional outcomes included objective measures of brain morphology/functioning or biochemical status. These measures were only extracted if they were related to changes in cognitive or academic performance.

### Organization process

Studies were first categorized by design: *1)* RCTs and non-RCTs, *2)* prospective studies beginning during infancy, and *3)* prospective studies beginning during early adolescence. Next, RCTs and non-RCTs were categorized according to dietary intervention. Prospective studies were categorized according to dietary intervention and pattern. Cognitive tests were also grouped into specific cognitive domains based on [24] where possible, and included general cognitive ability (intelligence), memory (visual, verbal, spatial, semantic, or working memory), executive function (verbal fluency, inhibition, reasoning, problem solving, planning, or mental flexibility/shifting), attention (sustained or selective), perception, and information processing speed. Using the United Nations classification system [25], countries were classified as either having a developed economy, developing economy, or an economy in transition.

### Risk of bias and certainty of evidence

The Cochrane Risk of Bias (RoB) 2 Tool [26] was used to assess RoB in cluster and noncluster RCTs. The Joanna Briggs Institute Critical Appraisal Tool for Quasi-Experimental Studies and Cohort Studies was used to assess RoB in non-RCTs and prospective studies, respectively. The following cut-offs were applied: low RoB if ≥ 71% of answers scored “yes,” moderate RoB if 51%–70% of answers scored “yes,” and high RoB if ≤ 50% of answers scored “yes.” RoB assessments were completed after data extraction by 2 independent authors (CMG and AM). If a consensus could not be reached after discussion, a third reviewer (HAY) was consulted. To obtain additional information, theses and study protocols were checked and study authors were contacted where possible. RoB outcomes are summarized in Supplemental Tables 4–8

## Results

### Study selection

**FIGURE 1:**
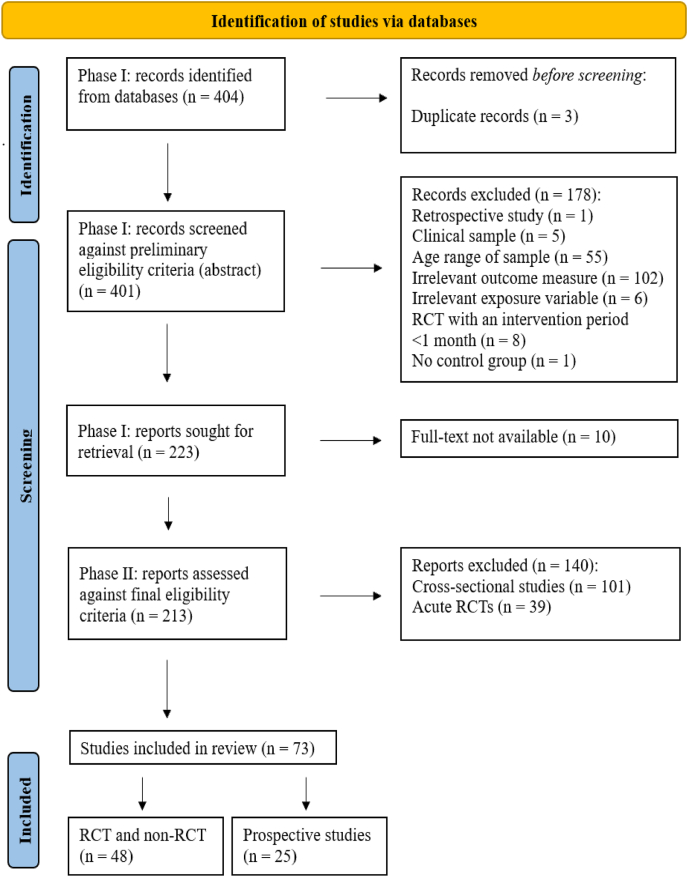
PRISMA flow diagram of the screening and literature selection process. Ten full-text reports could not be retrieved, including 4 multivitamin and mineral studies [[27], [28], [29], [30]]. RCT, randomized controlled trial.

The PRISMA flow chart is presented in Figure 1. A total of *N* = 404 publications were initially identified. In total, 213 full-text reports were assessed for eligibility, of which 140 were excluded, leaving 73 studies included in the review.

The final *N* = 73 studies were included in the systematic review, *n* = 48 RCT and non-RCT (Table 1), *n* = 13 prospective studies beginning during infancy (Table 2), and *n* = 12 prospective studies beginning during adolescence (Table 3). TABLE 1, TABLE 2, TABLE 3 provide a summary of the main findings, with fuller descriptions of study characteristics and outcomes in Supplemental Tables 9–11.

**TABLE 1:** Randomized and non-RCTs.

Author (y)	Sample	Dietary intervention	Cognition	School performance	Dietary/biochemical measures	Results
Choline (*n* = 1)						
O’Connor et al. [31]	*N* = 122, 9–13 y, United States (SCENE study)	9-mo double-blind RCT; egg powder vs. milk powder vs. placebo, incorporated into 10 weekly foods	NIH Toolbox: working memory, attention/inhibition, processing speed, visual memory, mental flexibility (composite nonverbal cognition)	—	Not central here	Selective attention and inhibitory control improved in the milk powder group vs. placebo; no clear cognitive benefit in the egg (higher choline) group
LCPUFA interventions (*n =* 10)						
Handeland et al. [12]	*N* = 426, 14–15 y, Norway, mixed SES, high baseline iodine/vitamin D deficiency and low omega-3 index (FINS-TEENS)	3-mo cluster-RCT; fatty fish 3×/wk vs. meat 3×/wk vs. fish oil capsules (equivalent omega-3 dose)	D2 Test of Attention (attention, processing speed)	Norwegian spelling and reading test (data not reported due to ceiling effects)	Not central in this paper	Fatty fish led to greater gains in processing speed than both control groups and better attention vs. supplements; some apparent benefits in the meat group on omissions disappeared after adjusting for compliance
Handeland et al. [32]	FINS-TEENS cohort as above	As Handeland et al. [12]	As Handeland et al. [12]	—	Fatty acids, vitamin D, ferritin, urinary iodine concentration	Omega-3 index and DHA increased most in the supplement group, then fish, least in meat; vitamin D, ferritin and iodine status did not relate to cognitive scores, so mediation was not supported
Kennedy et al. [33]	*N* = 86, 10–12 y, United Kingdom	2-mo double-blind RCT; low-dose DHA (400 mg/d), high-dose DHA (1000 mg/d) vs. placebo	2 computerized batteries: memory, attention, processing speed	—	Not reported here	Low-dose DHA improved delayed verbal memory vs. placebo (pre- and post-breakfast); high-dose DHA was associated with a decline in delayed verbal memory prebreakfast
Kirby et al. [34]	*N* = 348, 8–9 y, United Kingdom, mixed SES	17-wk double-blind RCT; fish oil + vitamins vs. placebo	IQ (KBIT-2), working memory, attention, impulsivity	Reading and spelling (WIAT-II)	Cheek cell fatty acid composition	EPA increased in the active group; per-protocol analyses suggested improved problem solving in compliers only, with no clear intention-to-treat effects on cognition or achievement
McNamara et al. [35]	*N* = 33 boys, 8–10 y, United States	2-mo double-blind RCT; low-dose DHA (400 mg/d), high-dose DHA (1200 mg/d) vs. placebo	Sustained attention (CPT-Identical Pairs)	—	DHA, AA, LA; fMRI brain activation	DHA levels rose sharply in supplemented groups; no behavioral change in attention, but DHA increased dorsolateral prefrontal activation and altered occipital/cerebellar activity vs. placebo during the task
Pinar-Martí et al. [36]	*N* = 771, 11–16 y, Spain, mixed SES	6-mo RCT; 30 g walnuts/d vs. habitual diet	Attention, working memory, nonverbal IQ (inductive reasoning)	—	Fatty acid composition	Intention-to-treat: no cognitive effects; per-protocol (>100 d intake): walnut group showed better attention and nonverbal IQ and higher ALA levels
Portillo-Reyes et al. [37]	*N* = 59, 8–12 y, Mexico, low SES, mild–moderate malnutrition	3-mo double-blind RCT; fish oil (180 mg DHA + 270 mg EPA/d) vs. placebo	Broad battery: processing speed, nonverbal IQ, memory, inhibition, mental flexibility, perceptual integration	Academic grades (language, mathematics, history, geography, science, civic education)	—	Supplementation improved processing speed, perceptual integration, inhibition and nonverbal IQ; >70% of the active group showed large, clinically meaningful gains on several measures vs. <25% of controls
Teisen et al. [38]	*N* = 199, 8–9 y, Denmark, mixed SES	4-mo RCT; 300 g oily fish/week vs. 300 g poultry/week	Attention, processing speed, spatial memory, working memory, inhibition, mental flexibility	—	Fatty acids and vitamin D	Omega-3 and vitamin D increased more in the fish group; they showed fewer processing speed errors and faster mental flexibility/shifting than the poultry group
van der Wurff et al. [39]	*N* = 256, 13–15 y, Netherlands, low omega-3 index (<5%; Food2Learn)	12-mo double-blind RCT; fish oil vs. placebo (dose adjusted by cohort)	Attention, processing speed, working memory, inhibition, mental flexibility	—	Fatty acid composition, omega-3 index	Omega-3 index and EPA/DHA increased in the active group but remained suboptimal; both groups improved cognitively over time with no consistent between-group differences, and changes in omega-3 index did not predict cognitive change
van der Wurff et al. [40]	Food2Learn cohort as above	As van der Wurff et al. [39]	—	Dutch, English, mathematics grades	Omega-3 index	No meaningful changes in grades and no association between omega-3 index and academic performance
Whole grains (*n =* 1)						
Chung et al. [41]	*N* = 28 boys, 15–17 y, Korea, high SES	9-wk single-blind RCT; wholegrain mix (rice, beans, walnuts) vs. polished rice, 3×/d	Attention, working memory, inhibition, processing speed, mental flexibility, memory, verbal fluency (computerized tests)	—	Hemoglobin, BDNF, S100B	Some aspects of attention were better preserved in the wholegrain group (AX-CPT performance), whereas delayed verbal memory improved more in the control group; BDNF increased slightly with wholegrains and decreased in controls
Nordic diet (*n =* 3)						
Sørensen et al. [42]	*N* = 739, 8–11.6 y, Denmark, mixed SES (OPUS School Meal Study)	6-mo within-school cluster-RCT (3-mo periods); NND lunches/snacks vs. packed lunches from home	D2 Test of Attention (attention, processing speed)	Standard Danish reading and mathematics tests	7-d food diary	NND meals increased intake of vegetables, fish, potatoes, fiber and key micronutrients and reduced saturated fat. Reading speed and accuracy improved, particularly in some subgroups; errors of omission and commission were higher after NND periods, with no overall benefit in attention/processing speed
Sørensen et al. [43]	OPUS cohort as above	As Sørensen et al. [42]	Underlying “school performance” and “reading comprehension” factors	Factor scores for “school performance” and “reading comprehension”	Fatty acids, hemoglobin, ferritin	NND increased omega-3 status and lowered n-6:n-3 ratio without changing ferritin/hemoglobin; NND was associated with better composite school performance and reading comprehension vs. control
Sørensen et al. [44]	OPUS cohort as above	As Sørensen et al. [42]	Reading and attention stratified by sex, parental education, baseline reading level	Standard Danish mathematics and reading; subgroups of poor vs. normal/good readers	Not central here	Effects varied by subgroup: increases in impulsivity and attentional errors occurred mainly in boys and children with higher parental education, whereas reading accuracy gains were also greater in these groups and in children with better baseline reading
Multinutrient interventions (*N* = 11)						
Chellappa and Karunanidhi [11]	*N* = 109 girls, 17–19 y, India, low–middle SES, high iron and zinc deficiency	4-mo double-blind RCT; iron, zinc, iron+zinc, or placebo	Processing speed, nonverbal IQ, attention, verbal and visual memory	—	Hemoglobin, ferritin, zinc	Iron and iron+zinc improved iron status; zinc alone increased zinc. All active groups showed better processing speed, with iron-containing groups also showing gains in visual memory
Haskell et al. [45]	*N* = 78, 8–14 y, United Kingdom	3-mo double-blind RCT; daily MVM (16 vitamins/minerals) vs. placebo	Attention, processing speed, spatial and verbal memory, semantic memory, delayed visual and verbal memory	—	Not reported here	MVM improved attention and processing speed at 1–3 mo vs. placebo; 1 visual memory outcome favored controls
Kalaichelvi [46]	*N* = 240 girls, 12–15 y, India, all with IDA	2-mo cluster-RCT; iron- and vitamin C–rich nutritional ball vs. habitual diet	Verbal and performance IQ	—	Hemoglobin	Hemoglobin normalized in many girls in the active group and remained low in controls; verbal IQ was higher in the intervention group at both postintervention follow-ups
Lynn and Harland [47]	*N* = 413, 12–15 y, United Kingdom; small proportion anemic/iron deficient	4-mo double-blind RCT; iron + vitamin C vs. placebo	Nonverbal IQ	—	Ferritin, hemoglobin stratified by iron status	Little overall effect in the full sample; among iron-deficient participants, iron supplementation produced a clinically meaningful gain (∼6 IQ points difference) in nonverbal IQ vs. placebo
Perlman et al. [48]	*N* = 684, 8–12 y, United States, low SES, many below RDAs for key nutrients	8-mo double-blind RCT; school-based MVM vs. placebo (weekdays only)	—	Standardized achievement (TerraNova) and GPA (reading, language, mathematics, science, social sciences)	Not central here	No significant effects on academic test scores or GPA
Petrova et al. [49]	*N* = 103, 8–14 y, Spain	5-mo double-blind RCT; fortified milk (DHA, vitamins, minerals) vs. regular milk	Working memory and processing speed (WISC-IV subtests)	—	Lipids, iron, ferritin, DHA, calcium, vitamins D and E	Fortified milk improved DHA and vitamin D; working memory improved more in the active group, and a small processing-speed benefit was fully mediated by changes in vitamin D, not DHA
Schoenthaler et al. [50]	*N* = 615, 12–16 y, United States, mixed SES	13-wk cluster-RCT; 3 doses of MVM vs. placebo	IQ (verbal and nonverbal), nonverbal reasoning, processing speed	CTBS (broad academic achievement)	Micronutrient status (not fully reported)	Medium/high-dose MVM produced modest gains in nonverbal IQ, especially in compliant participants; effects on academic achievement were less clear
Sen and Kanani [51]	*N* = 161 girls, 9–13 y, India, low SES, high anemia rates	12-mo cluster-RCT; 3 doses of iron–folic acid vs. habitual diet	Working memory, planning, attention, visual memory	—	Hemoglobin	All iron doses increased hemoglobin, especially in anemic girls; medium and high doses improved performance across all cognitive tests, with more limited benefits at the lowest dose
Southon et al. [52]	*N* = 51, 13–14 y, United Kingdom	4-mo nonrandomized blinded trial; daily MVM vs. placebo	Verbal and nonverbal IQ	—	Hemoglobin, iron, ferritin, zinc, copper, selenium, folate, vitamins B1, B2, B6, B12, C, D3	MVM increased vitamin B12 and folate but had no measurable impact on IQ
Snowden [53]	*N* = 30, 9–10 y, United Kingdom, mixed SES	10-wk triple-blind RCT; daily MVM vs. placebo	Verbal and nonverbal IQ	—	—	Nonverbal IQ increased more in the MVM group than in the placebo group; verbal IQ did not differ
Wang et al. [54]	*N* = 296, 12–14 y, China, low SES, high vitamin B2/selenium deficiency	6-mo cluster-RCT; fortified milk vs. unfortified milk	—	Academic grades in Chinese, mathematics, English, physics, social science, and ethics	Ferritin and vitamins B2, B12, D, and selenium	Fortified milk reduced B2 and iron deficiency and led to larger improvements in Chinese, mathematics, English, and ethics scores than unfortified milk
Iron (*n =* 12)						
Buzina-Suboticanec et al. [55]	*N* = 50, ∼9 y, rural Croatia, high prevalence of mild anemia and micronutrient deficiencies	10-wk double-blind cluster-RCT; oral iron vs. placebo	IQ (verbal, nonverbal, full-scale) and processing speed	—	Transferrin saturation, iron, zinc, vitamins A, B2, E, and C	Iron improved hemoglobin and transferrin saturation and led to gains in processing speed and nonverbal/overall IQ, especially in children with lower baseline hemoglobin
Bruner et al. [10]	*N* = 81 girls, 13–18 y, United States, iron deficient	2-mo double-blind RCT; ferrous sulfate vs. placebo	Attention, processing speed, verbal memory	—	Hemoglobin, ferritin	Iron improved iron status and immediate verbal memory vs. placebo
Devaki et al. [56]	*N* = 120, 15–18 y, India, mix of IDA, ID and normal iron status	8-mo non-RCT; iron supplementation in IDA/ID and 1 normal-iron group vs. normal-iron control	Immediate and delayed verbal memory, nonverbal IQ, full-scale IQ	Mathematics test	Hemoglobin, ferritin	Iron-treated groups showed improved iron status and broad gains in memory, IQ and maths performance, particularly in IDA/ID adolescents; no comparable improvement in controls
Kashyap and Gopaldas [57]	*N* = 130 girls, 8–15 y, India, low SES, ∼90% anemic	2-mo RCT cycles during a school year; ferrous sulfate vs. placebo	Working memory, planning, attention, visual memory	—	Hemoglobin	Anemia prevalence fell dramatically during supplementation and rebounded after; cognitive gains in working memory, planning and attention at the end of the second term were greater in the iron group
Karkada et al. [58]	*N* = 60 girls, 11–17 y, India, low SES, 50% mildly anemic	3-mo non-RCT; ragi-based iron-rich food in anemic girls vs. usual diet in nonanemic controls	—	Academic grades	Hemoglobin	Anemia largely resolved in the intervention group and increased in controls; academic grades did not change significantly
Lambert et al. [59]	*N* = 116 girls, 12.5–17.9 y, New Zealand, iron deficient	2-mo double-blind RCT; ferrous sulfate vs. placebo	Verbal memory, attention, inhibition, working memory	—	Ferritin, hemoglobin	Iron improved ferritin and prevented hemoglobin decline; immediate verbal memory improved in the iron group (especially for later list items), and changes in iron indices correlated with improvements in recent recall and working memory
Pollitt [60]	*N* = 68, 8–11 y, Egypt, low SES, 41% with IDA	4-mo double-blind RCT; ferrous sulfate vs. placebo	Attention, verbal IQ, impulsivity	—	Ferritin, hemoglobin	Hemoglobin increased in IDA children regardless of group but declined in non-IDA children on placebo; among IDA children, iron improved impulsivity vs. placebo
Pollitt et al. [61]	*N* = 1358, 9–12 y, Thailand, low SES, small proportion with IDA/ID	4-mo double-blind RCT; escalating ferrous sulfate dose vs. placebo plus deworming	Nonverbal IQ	Educational Achievement Test (language, mathematics)	Ferritin, hemoglobin	Iron improved hemoglobin in IDA and ID children but produced no detectable effects on IQ or academic achievement at group level
Rezaeian et al. [62]	*N* = 200 girls, 14–18 y, Iran, ∼20% anemic	4-mo single-blind cluster-RCT; twice-weekly ferrous sulfate vs. habitual diet	Attention (Toulouse–Piéron)	—	Hemoglobin	Iron increased hemoglobin and produced larger gains in attention scores than in the control group
Scott et al. [63]	*N* = 140, 12–16 y, India, low SES, high iron deficiency/anemia	6-mo double-blind RCT; iron-biofortified pearl millet vs. control millet	Processing speed, attention, visual memory, inhibition	—	Hemoglobin, ferritin, body iron	Biofortified millet improved iron stores and led to larger improvements in processing speed, attention and related cognitive tasks than control millet
Soemantri et al.[64]	*N* = 119, ∼11 y, Indonesia, low SES, 66% with IDA	3-mo double-blind RCT; ferrous sulfate vs. placebo	Attention and nonverbal IQ	Educational Achievement Test (biology, mathematics, social science, language)	Hemoglobin	In IDA children, iron increased hemoglobin and led to greater gains in academic achievement than placebo; non-IDA children still outperformed treated IDA children postintervention
Soemantri et al. [65]	*N* = 130, ∼10 y, Indonesia, low SES, 45% with IDA	3-mo double-blind RCT; ferrous sulfate vs. placebo	Nonverbal IQ	Educational Achievement Test	Hemoglobin	Iron-normalized hemoglobin in IDA children and maintained levels at 3-mo follow-up; no clear IQ effects and educational outcomes were not formally compared between groups
Iodine (*n* = 4)						
Gordon et al. [66]	*N* = 184, 10–13 y, New Zealand, low SES, mild iodine deficiency	6-mo double-blind RCT; daily iodine vs. placebo	Working memory, nonverbal IQ, processing speed	—	Urinary iodine concentration, total thyroxine	Iodine supplementation restored normal iodine status and improved nonverbal IQ, with no clear effects on working memory or processing speed
Huda et al. [67]	*N* = 305, 8–10 y, Bangladesh, severely iodine-deficient areas	4-mo double-blind RCT; single iodized oil capsule vs. placebo	Broad battery: attention, memory, nonverbal IQ, processing speed, inhibition	—	Urinary iodine, TSH, total thyroxine	Iodine status improved but remained mostly in the mildly deficient range; no detectable cognitive benefits over 4 mo
Isa et al. [68]	*N* = 165, ∼11 y, Malaysia, moderately–severely iodine-deficient villages	12-mo non-RCT; single iodized oil capsule vs. habitual diet	Nonverbal IQ (percentile bands)	—	Urinary iodine, thyroid volume	Iodine status and thyroid volume improved in both groups but more so with supplementation; IQ percentile distributions shifted upward over time, with somewhat different patterns between groups
Zimmermann et al. [69]	*N* = 310, 10–12 y, Albania, severely iodine-deficient areas	6-mo double-blind RCT; single iodized oil capsule vs. placebo	Processing speed, nonverbal IQ, working memory	—	Urinary iodine, thyroid volume, thyroid hormones	Iodine substantially improved thyroid and iodine status and led to greater improvements in processing speed and nonverbal IQ vs. placebo
Vitamin D (*n* = 1)						
Grung et al. [70]	*N* = 50, 13–14 y, Norway, vitamin D deficient/insufficient	3-mo double-blind RCT; daily vitamin D vs. placebo in winter	Problem solving (Tower of Hanoi, Tower of London)	—	Vitamin D levels	Supplementation normalized vitamin D and improved performance on more complex Tower of Hanoi levels; easier tasks and Tower of London performance were unchanged
Polyphenols (*n* = 2)						
Nidich et al. [71]	*N* = 34, 8–9 y, United States	5-mo double-blind RCT; Maharishi Ayur-Ved Student Rasayana vs. placebo	Nonverbal IQ (Cattell Culture Fair)	—	—	Herbal supplementation led to a larger increase in nonverbal IQ than placebo
Tefagh et al. [72]	*N* = 86 girls, 15–17 y, Iran, average SES	6-wk double-blind RCT; Ustukhuddus Alavi herbal supplement vs. placebo	Working memory, sustained attention, processing speed (Paced Auditory Serial Addition Test)	—	—	Working memory was better in the active group at 3 wk; sustained attention was better in the active group at 3 and 6 wk
SBPs (*n* = 3)						
Cueto and Chinen [13]	*N* = 590, ∼12 y, Peru, low SES, 20 schools (full vs.-multigrade)	3-y non-RCT; government SBP providing fortified mid-morning breakfast vs. no SBP	Visual memory, processing speed	Unstandardized mathematics and Spanish reading comprehension	—	In multigrade schools, SBPs were associated with better picture recognition, mathematics and reading; in full-grade schools, SBP schools performed worse academically than controls
Murphy et al. [73]	*N* = 4350, 9–11 y, United Kingdom, mixed SES, 111 schools	12-mo cluster-RCT; Primary School Free Breakfast Initiative vs. waitlist control	Verbal memory (Word List Recall; subsample)	—	Breakfast quality (number of healthy items consumed)	The intervention improved consumption of healthy breakfast items but did not affect verbal memory; SES of school did not modify the effect
Shemilt et al. [74]	*N* = 6042, ∼9.9 y, United Kingdom, low SES, 27 schools	12-mo cluster-RCT; funding for SBP vs. no funding (analysis limited to 3 mo due to contamination)	Processing speed, mental flexibility (TMT-A, TMT-B)	—	—	At 3 mo, SBP funding was associated with faster processing speed; later data were not interpretable due to contamination of the control group

Abbreviations: AA, arachidonic acid; ALA, alpha-linolenic acid; BDNF, brain-derived neurotrophic factor; CPT, continuous performance test; cluster-RCT, cluster randomized controlled trial; CTBS, comprehensive tests of basic skills; D2, D2 Test of Attention; fMRI, functional MRI; GPA, grade point average; ID, iron deficiency; IDA, iron-deficiency anemia; KBIT-2, Kaufman Brief Intelligence Test, Second Edition; LA, linoleic acid; LCPUFA, long-chain PUFA; MVM, multivitamin and mineral; *N*, sample size; NIH Toolbox, NIH Toolbox cognitive battery; NND, New Nordic Diet; RCT, randomized controlled trial; RDA, recommended dietary allowance; S100B, S100 calcium-binding protein B; SBP, school breakfast program; SES, socioeconomic status; TSH, thyroid-stimulating hormone; TMT-A/TMT-B, Trail Making Test parts A and B; WIAT-II, Wechsler Individual Achievement Test, Second Edition; WISC-IV, Wechsler Intelligence Scale for Children, Fourth Edition; FINS-TEENS, Fish Intervention Studies-TEENS; OPUS, Optimal well-being, development and health for Danish children through a healthy New Nordic Diet; AX-CPT, AX-Continuous Performance Test.

**TABLE 2:** Longitudinal studies that examined the effect of diet quality during infancy (<3 y) on adolescent outcomes.

Author (y)	Sample	Dietary exposure/infant exposure/status	Cognitive measures (follow-up)	Academic measures (follow-up)	Biochemical/neurological measures	Main findings (adjusted/long-term)
General diet quality (*n =* 10)						
Feinstein et al. [75]	*N =* 5471, United Kingdom, ALSPAC; diet at 3 y; grades at 10–11 y; mostly middle SES	43-item caregiver FFQ at 3 y; PCA: junk food, health-conscious, traditional patterns	—	Standardized English, maths, science at 10–11 y (combined score)	—	Higher junk food pattern at 3-y predicted lower academic scores at 10–11 y; effect attenuated but remained significant after extensive maternal/child covariates. Health-conscious pattern was initially positive but became nonsignificant after adjustment.
Golley et al. [76]	*N =* 4429, United Kingdom, ALSPAC; diet at 6 mo; IQ at 8.5 y; mostly middle SES	Complementary Feeding Utility Index at 6 mo (breastfeeding duration, timing of solids, exposure to iron-rich/textured/HFSS foods)	WISC-III total, verbal, performance IQ at 8.5 y	—	—	Higher feeding index at 6 mo was associated with higher total, verbal, and performance IQ after adjusting for child and maternal factors. After additional adjustment for maternal IQ, the association with performance IQ was lost, but total and verbal IQ remained related.
Mou et al. [77]	*N =* 1888, Netherlands, Generation R; diet at 1 y; IQ at 13 y	221-item caregiver FFQ at 1 y; diet quality score and PCA patterns: vegetables/potatoes/grains; snacks/processed/sugars; butter/margarines/whole grains/dairy	WISC-V full-scale IQ at 13 y	—	MRI brain morphology at 10 y	Snacks/processed/sugars pattern at 1 y was not directly associated with IQ at 13 y, but related to lower cerebral white matter volume at 10 y. White matter volume statistically mediated the association between this dietary pattern and later IQ.
Northstone et al. [78]	*N =* 7044, United Kingdom, ALSPAC; diet at 3 y; IQ at 8.5 y; mostly middle SES	Caregiver FFQ at 3 y; PCA patterns: processed, traditional, health-conscious, snack	WISC-III full-scale, verbal, performance IQ at 8.5 y	—	—	Processed pattern at 3 y was associated with lower full-scale IQ at 8.5 y after adjustment. Snack pattern was positively associated with full-scale IQ. Health-conscious pattern’s positive association with IQ was no longer significant after covariate adjustment.
Nyaradi et al. [79]	*N =* 1346–1455, Australia, Raine Gen2; diet at 1–3 y; IQ at 10 y; mixed SES	24-h recalls at 1, 2, 3 y; EAT diet score (higher = more guideline-concordant diet: wholegrains, veg, fruit, dairy, white meat, legumes; less red/processed meat, snacks, SSBs)	Peabody Picture Vocabulary Test-III (verbal IQ); Raven’s Matrices (nonverbal IQ) at 10 y	—	—	Higher EAT score at 1 y predicted higher verbal and nonverbal IQ at 10 y after adjusting for sociodemographic and family factors. Diet scores at 2–3 y predicted nonverbal IQ before adjustment, but not after. Sweetened beverages at 1 y were negatively, and fruit and dairy intake positively, associated with IQ components.
Nyaradi et al. [80]	*N =* 717, Australia, Raine Gen2; diet at 1 y; cognition at 17 y; mixed SES	As Nyaradi et al. [79]	CogState at 17 y: processing speed (Detection, Identification), visual and spatial memory (1 Card Learning, CPAL)	—	—	Higher EAT score at 1 y was associated with faster information processing speed at 17 y. No associations were found with visual or spatial memory.
Nyaradi et al. [81]	*N =* 2287, Australia, Raine Gen2; diet at 1–3 y; academics at 10 and 12 y; mixed SES	As Nyaradi et al. [79]	—	Western Australian literacy and numeracy tests (reading, writing, spelling, maths) at 10 and 12 y	—	Higher EAT scores at 1–3 y were consistently associated with better reading, writing, spelling, and mathematics scores at 10 and 12 y. Fruit intake at 1 y and dairy intake at 1–3 y were independently and positively associated with multiple academic outcomes.
Smithers et al. [82]	N ≈ 7052/5610/6366, United Kingdom, ALSPAC; diet at 6, 15, 24 mo; IQ at 8.5 y	Caregiver FFQs at 6, 15, 24 mo; PCA patterns: homemade traditional, ready-to-eat, discretionary snacks/soft drinks; plus breastfeeding (6 mo) and contemporary (15, 24 mo) patterns	WISC-III full-scale, verbal, performance IQ at 8.5 y	—	—	Discretionary snack/soft-drink patterns at 6, 15, and 24 mo were consistently associated with lower IQ scores at 8.5 y. Traditional pattern at 6 mo, breastfeeding pattern at 6 mo, and contemporary patterns at 15–24 mo were positively associated with IQ. Ready-to-eat pattern showed mixed associations (early negative, later positive) with IQ.
Smithers et al. [83]	*N =* 7652, United Kingdom, ALSPAC; diet 6–24 mo; IQ at 8 and 15 y	Caregiver FFQs at 6, 15, 24 mo; trajectories for healthy, discretionary, traditional, ready-to-eat patterns	WISC-III IQ at 8 y; WASI IQ at 15 y	—	—	A healthy dietary trajectory across infancy was associated with higher full-scale and verbal IQ at 8 y but not at 15 y. Traditional and discretionary trajectories were associated with lower full-scale IQ at 15 y, with less clear effects at 8 y.
Zhu et al. [84]	*N =* 745, rural China; diet 6–23 mo; cognition 10–12 y; mixed SES; all breastfed; offspring of prenatal micronutrient RCT participants	Caregiver FFQs at 6, 9, 12, 18, 24 mo; composite feeding score based on WHO complementary feeding guidelines and use of iron-rich/fortified foods; timing of cows’ milk and high-protein foods	WISC-IV full-scale and nonverbal IQ, verbal comprehension, working memory, processing speed at 10–12 y	—	—	Higher composite feeding scores showed a dose–response relationship with better IQ, verbal comprehension, working memory, and processing speed. Regular consumption of iron-rich/fortified foods between 6 and 24 mo was associated with better performance across all cognitive domains. Very early introduction of high-protein foods and very late introduction of cows’/goats’ milk were linked with less favorable IQ profiles.
Iron (*n =* 3)						
Algarin et al. [85]	*N =* 132, Chile, low SES; infants with/without IDA at 6, 12, or 18 mo; all treated with iron; follow-up at 10 y	All infants received oral iron for ≥6–12 mo; groups defined retrospectively by IDA vs. non-IDA status in infancy	Go/No-Go task (inhibitory control) at 10 y	—	ERPs (N2 latency, P300 amplitude) at 10 y; current iron status	Despite normal iron status at 10 y, those who had IDA in infancy showed poorer inhibitory control, longer N2 latency, and smaller P300 amplitude than peers without prior IDA, suggesting persistent neurofunctional differences.
Lozoff et al. [86]	*N =* 167, Costa Rica, low SES; infants 12–23 mo in RCT; follow-up at 11–14 y	3-mo double-blind infant trial; iron-sufficient infants got placebo; those with ID/IDA received therapeutic iron. Follow-up groups: good iron status vs. chronic ID across treatment	Wide battery at 11–14 y: IQ, perception, selective and auditory attention, processing speed, spatial memory, general abilities	School grades; reading and arithmetic skills; directed writing at 11–14 y	Current iron status; home environment and maternal IQ/education covariates	Adolescents with chronic iron deficiency (not fully corrected in infancy) had poorer arithmetic, writing, selective attention, and spatial memory than those whose iron deficiency resolved or who were always sufficient. Differences in IQ scores were attenuated after covariate adjustment.
Lukowski et al. [87]	*N =* 114, follow-up of the Costa Rica RCT; cognitive testing at 19 y	Same infant trial and status groups as Lozoff et al. [86]	At 19 y: tests of processing speed, mental flexibility/shifting, planning, spatial working memory, visual memory	—	Current iron status and home/maternal factors	Young adults with residual iron deficiency in infancy showed poorer mental flexibility/shifting and planning abilities than those with normal iron status in infancy, indicating long-lasting executive function differences despite later treatment.

Abbreviations: ALSPAC, Avon Longitudinal Study of Parents and Children; CogState, computerized cognitive test battery (CogState); CPAL, Continuous Paired Associate Learning (CogState task); ERP/ERPs, event-related potential(s); FFQ, food frequency questionnaire; HFSS, high fat, salt and sugar foods; ID, iron deficiency; IDA, iron-deficiency anemia; IQ, intelligence quotient; N, sample size; N2, negative-going ERP component (∼200 ms post-stimulus); N/A, not assessed / not available; P300, positive ERP component (∼300 ms post-stimulus); PCA, principal component analysis; RCT, randomized controlled trial; SES, socioeconomic status; SSB, sugar-sweetened beverages; WASI, Wechsler Abbreviated Scale of Intelligence; WISC-III/IV/V, Wechsler Intelligence Scale for Children (Third/Fourth/Fifth Edition); 24-h recall, 24-hour dietary recall; EAT, Eating Assessment in Toddlers; Gen2, Generation 2.

**TABLE 3:** Prospective studies examining the effect of diet during early adolescence on outcomes during late adolescence.

Author (y)	Sample	Exposure (fish/diet/breakfast)	Cognitive measures	Academic measures	Biochemical/neurological	Main findings (adjusted)
Fish intake (*n =* 2)						
Åberg et al. [88]	*N =* 3972 males, Sweden; diet at 15 y, cognition at 18 y; mixed SES	Self-reported fish frequency at 15 y: >1×/wk, 1×/wk, <1×/wk	Conscription intelligence tests at 18 y: combined IQ, verbal abilities, visuospatial abilities	—	—	Higher fish intake at 15 y was strongly positively associated with combined IQ, verbal and visuospatial abilities at 18 y. Those eating fish >1×/wk had significantly higher IQ and better verbal/visuospatial scores than those eating fish <1×/wk after adjustment for sociodemographic and lifestyle covariates.
Kim et al. [89]	*N =* 9448 (4674 males, 4774 females), Sweden; diet at 15 y, grades at 16 y; mixed SES	Self-reported fish frequency at 15 y: >1×/wk, 1×/wk, <1×/wk	—	Standardized school grades at 16 y (sum of 16 subjects)	—	Compared with <1×/wk, fish 1×/wk and >1×/wk at 15 y were both associated with higher average grades at 16 y, with the largest grade difference in the >1×/wk group. Effects remained after adjusting for socioeconomic and lifestyle factors.
General dietary quality (*n =* 7)						
Dubuc et al. [90]	*N =* 187, Canada, elite school; diet, cognition, grades at 12–13 y and 15–16 y	Self-reported number of meals/day, fruit and veg servings, and breakfast frequency on weekdays/weekends at baseline and 3-y follow-up	Arrow Flankers (attention/inhibition) and n-back (working memory) at baseline and 3-y follow-up	Standardized grades in science, mathematics, language (average of 9 subjects) at baseline and 3-y follow-up	—	In girls, lifestyle changes (diet, sleep, activity, screen/social media time) did not predict change in grades, but baseline daily meal intake predicted change in attention and working memory. In boys, increased fruit and vegetable intake from baseline to follow-up was associated with improved average grades.
Faught et al. [91]	*N =* 4253 (2007 males, 2246 females), Canada, CLASS study; diet at 10–11 y, grades at 12–13 y; mixed SES	Harvard FFQ; adherence to Canada’s Food Guide recommendations for servings of fruit and veg, grains, milk/alternatives, meat/alternatives, and free sugars/saturated fat on previous day	—	Standardized tests in reading, writing, mathematics at 12–13 y (meeting vs. not meeting expectations)	—	After adjustment for covariates and other lifestyle behaviors, meeting milk or meat recommendations at baseline predicted higher odds of meeting mathematics expectations. Meeting all food-group recommendations (except fruit and veg) predicted meeting reading expectations. Meeting recommendations for meat, free sugars, sleep, or screen time predicted meeting writing expectations.
Faught et al. [92]	*N =* 11,016 (5288 males, 5728 females), Canada, COMPASS; diet 13–18 y, grades 14–19 y; mixed SES	Self-reported previous-day intake vs. Canada’s Food Guide (fruit and veg, grains, milk/alternatives, meat/alternatives) at baseline and 1-y follow-up	—	Self-reported mathematics and English grades at baseline and 1-y follow-up	—	Compared with those never meeting recommendations, meeting meat recommendations at either/both time points, meeting milk recommendations at both time points, or meeting fruit and veg recommendations at follow-up were each associated with better mathematics grades. Meeting meat recommendations at follow/both time points or fruit and veg recommendations at follow-up only was associated with better English grades.
Mou et al. [77]	*N =* 2326, Netherlands, Generation R; diet at 8 y, IQ at 13 y; mostly middle SES	221-item caregiver FFQ at 8 y; diet-quality score and PCA patterns including “whole grains, soft fats, and dairy”	WISC-V full-scale IQ at 13 y	—	MRI brain morphology at 10 y (total brain, cerebral grey matter, etc.)	Higher adherence to the whole grains/soft fats/dairy pattern at 8 y was associated with greater total brain and cerebral grey matter volumes at 10 y. These brain volumes, in turn, statistically mediated the association between this dietary pattern and higher IQ at 13 y. No associations were seen with hippocampal or amygdala volume.
Nigg and Amato [93]	*N =* 334 (150 males, 184 females), Hawaii, Fun 5 after-school program; baseline 9–12 y, follow-up 14–17 y	Self-reported daily servings of fruit & vegetables at baseline and 5-y follow-up	—	Self-reported average academic grade at 5-y follow-up	—	Higher fruit and vegetable intake at baseline unexpectedly predicted lower academic grades 5 y later after adjusting for sex and ethnicity. (Direction is opposite to most other diet–achievement findings.)
Nyaradi et al. [94]	*N =* 602, Australia, Raine Gen2; diet at 14 y, cognition at 17 y; mixed SES	212-item caregiver FFQ at 14 y; “healthy” pattern (fruit, veg, whole grains, legumes, fish) and “Western” pattern (soft drinks, fried/refined foods, takeaways, red/processed meat)	CogState battery at 17 y: processing speed (Detection), attention (Identification), visual memory (One Card Learning), spatial memory (CPAL, Groton Maze Learning)	—	—	Western dietary pattern was associated with slower processing speed and poorer delayed spatial memory. Lower intake of leafy greens and fruit was related to slower processing speed and poorer spatial memory, respectively. Higher intake of fried potatoes, crisps, and red meat was associated with slower processing speed, poorer attention, and poorer visual and spatial memory.
Purtell and Gershoff [95]	*N =* 8544 (4357 males, 4187 females), United States, ECLS-K; diet at 10–11 y, grades at 13–14 y; mixed SES	Self-reported fast-food frequency in past week at 10–11 y: none, 1–3 times, 4–6 times, daily	—	Standardized tests in literacy, mathematics, and science at 13–14 y	—	Children who ate fast food daily at 10–11 y had poorer grades in mathematics, literacy, and science at 13–14 y than those who ate none. Any fast-food intake was associated with smaller gains in maths scores over time, after extensive adjustment for diet, SES, health, and lifestyle covariates.
Mediterranean diet (*n =* 1)						
Hayek et al. [96]	*N =* 563 (283 males, 280 females), Lebanon; 15–18 y; 7 schools (4 private, 3 public); mixed SES	64-item FFQ; adherence to Mediterranean diet via KIDMED index at baseline	—	Self-reported average academic grade at 6- and 12-mo follow-up	—	Increases in KIDMED (Mediterranean diet adherence) over time were associated with increases in academic achievement. The positive association between higher adherence and better grades was evident at both 6 and 12 mo, independent of parenting style, school type, and sociodemographic covariates.
SBPs (*n =* 2)						
Murphy et al. [97]	*N =* 133 (58 males, 75 females), United States; 8–14 y; 3 schools; low SES (>70% eligible for free/reduced meals)	Change in participation in SBP over 4 mo: increased, decreased, or unchanged frequency of school breakfast	—	Mathematics, science, social studies, and reading grades at baseline and 4-mo follow-up	—	Pupils who increased SBP participation showed significantly larger gains in mathematics grades over 4 mo compared with those whose participation stayed the same or decreased.
Powell et al. [98]	*N =* 115, Jamaica; 12–13 y; 1 rural school; low SES; undernourished sample	3-mo SBP: 1 class received 100 mL milk plus cake or meat pastry daily; other classes received syrup drink or no breakfast	—	Wide Range Achievement Test (arithmetic, spelling, reading) at baseline and 3-mo follow-up	—	Provision of a substantive school breakfast (milk + food) for 3 mo led to greater improvements in arithmetic scores than syrup drink or no breakfast. Effects on spelling and reading were less clear.

Abbreviations: Arrow Flanker task, measure of attention and inhibitory control; CLASS study, Canadian cohort name as reported by authors; CogState, computerized cognitive test battery (CogState); COMPASS, Canadian school-based cohort study name as reported by authors; CPAL, Continuous Paired Associate Learning (CogState task); ECLS-K, Early Childhood Longitudinal Study, Kindergarten cohort; FFQ, food frequency questionnaire; KIDMED, Mediterranean Diet Quality Index for children and adolescents; PCA, principal component analysis; *N*, sample size; n-back, working memory task paradigm; SBP, school breakfast program; SES, socioeconomic status; WISC-V, Wechsler Intelligence Scale for Children, Fifth Edition; Gen2, Generation 2.

### Randomized and non-RCTs

#### Study characteristics

Of the 48 controlled trials, 30 were RCTs, 2 were non-RCTs, 13 were cluster-RCTs, and 3 were cluster non-RCTs. Three studies used a within-subjects design [[42], [43], [44]], and the remaining 45 studies used a between-subjects design. Of the RCTs, 28 were double-blind. Mean age ranged from 8.7 [71] to 18.4 y [11], and intervention periods ranged from 6 wk [72] to 3 y [13]. Most enrolled both male and female adolescents, apart from 2 studies that only enrolled males [35,41] and 9 studies that only enrolled females [[10], [11], [46], [51], [57], [58], [59], [62], [72]]. Dietary interventions included a range of nutrients and foods, including iron (*n* = 12), iodine (*n* = 4), vitamin D (*n* = 1), choline (*n* = 1), polyphenols (*n* = 2), O3 fatty acids (*n* = 10), multiple micronutrients (*n* = 11), and whole grains (*n* = 1). Studies also examined the effects of the New Nordic Diet (NND; *n* = 3) and SBPs (*n* = 3).

#### RoB and certainty of evidence

Thirty-Five RCTs showed some concerns of bias (Supplemental Tables 4 and 5), mostly because the method of randomization and/or allocation sequence was not reported, a preregistered protocol was not located, and it was unclear whether participants and/or outcome assessors were blinded. Eight studies showed a high RoB, mostly because participants and/or outcome assessors were not blind, baseline differences between intervention groups may reflect inadequate randomization, and dietary compliance was poor. Cluster-RCTs were additionally judged with some concerns of bias if it was unclear whether participants were identified before cluster randomization, and with high concerns of bias if data were not available for all clusters and participants within clusters. Three of 5 non-RCTs were judged as having a moderate RoB, generally due to baseline differences between groups, incomplete follow-up, and a lack of information on statistical methods.

#### Effect of diet on cognitive and academic performance during adolescence

##### Iron

Twelve studies (10 RCTs, 2 non*-*RCTs) evaluated iron supplementation in adolescents across a range of countries, with most conducted in lower-income contexts. Interventions ranged from 2 to 8 mo and used various iron formulations, most commonly ferrous sulfate. Although all studies were of sufficient duration to improve ferritin or hemoglobin, most were <4 mo, limiting potential for sustained cognitive change.

Effects on cognition were mixed. Improvements in verbal memory were reported in 3 studies, particularly among iron-deficient or anemic females [10,56,59]. One study also found benefits in visual memory after 6 mo [63]. Working memory improved in 1 study [57] but not others.

Across 5 studies assessing nonverbal IQ, positive effects were found only in adolescents with lower baseline hemoglobin or iron deficiency (ID) [55,56]. Two studies reported improvements in full-scale IQ, again mostly in those with ID or iron deficiency anemia (IDA). No effects were observed on verbal IQ. Improvements in planning [57] and inhibitory control [63] were also noted in isolated studies.

Seven studies assessed attention; 3 reported significant improvements, typically in participants with anemia or mixed iron status [62,63]. Some benefits were also observed for information processing speed [62,63], though effects were inconsistent. One study reported improved impulsivity scores, though interpretation was complicated by group-wide hemoglobin improvements [60].

Four studies examined academic performance using either standardized tests or school grades. Findings were inconsistent. Soemantri et al. [64] reported domain-specific academic gains in IDA-treated participants, whereas other studies found no effect or failed to test for group differences. Devaki et al. [56] observed improvements in mathematics scores, particularly in those with low baseline iron.

In summary, iron supplementation shows modest benefits for certain cognitive domains, particularly verbal memory and IQ measures, primarily in adolescents with poor iron status. Effects on academic outcomes and other cognitive subdomains were mixed. Interpretation is complicated by short intervention durations, heterogeneity in participant iron status, and potential comorbid nutritional deficiencies.

##### Iodine

Four studies (3 RCTs, 1 non-RCT) examined the effects of iodine supplementation on cognitive outcomes in adolescents from New Zealand, Malaysia, Bangladesh, and Albania. All samples were iodine-deficient, ranging from mild deficiency with normal thyroid function to moderate-to-severe deficiency with hypothyroidism. Interventions included daily supplementation or Lipiodol administration over 4–12 mo. Most studies reported improved iodine status, though one [67] showed minimal posttreatment improvement.

Three studies reported improvements in nonverbal IQ after iodine treatment, especially in adolescents with more severe deficiency [66,68,69]. However, findings from [68] were compromised by apparent control group contamination, and baseline group differences may have influenced results in other studies. The fourth study [67] found no improvement in nonverbal IQ, likely due to inadequate restoration of iodine status.

There was little evidence that iodine improved other cognitive domains. No improvements were observed for working memory, verbal memory, spatial memory, or executive functions (e.g., inhibitory control, verbal fluency, attention). Information processing speed improved in 1 study only [69], with no changes reported in the others.

In summary, iodine supplementation may improve nonverbal IQ in iodine-deficient adolescents if status is sufficiently restored. Effects on other cognitive domains are limited. Methodological issues, including nonrandomization, baseline imbalances, and insufficient repletion, constrain interpretation across studies.

##### Choline

A double-blind RCT in the United States [31] examined the effects of 10 weekly meal replacements made with whole egg (choline), milk, or gelatin powder on cognition over 9 mo. Improvements in attention and inhibitory control were observed only in the milk group. No changes were found in other cognitive domains. Low compliance in the egg group and a lack of choline biomarker data limited interpretation of the choline-specific effects.

##### Vitamin D

An RCT in Norway [70] tested the effects of daily vitamin D supplementation (38 μg) over 3 mo on problem solving and planning. Only the supplemented group achieved vitamin D sufficiency postintervention. Improvements were observed with the more demanding levels of the Tower of Hanoi task, but not on simpler levels or on the Tower of London, possibly due to differing executive demands.

##### Polyphenols

Two RCTs investigated polyphenol-rich supplements. Nidich et al. [71] found that 5 mo taking a herbal food supplement (Maharishi Ayur-Ved Student Rasayana) improved nonverbal IQ, compared with placebo. Tefagh et al. [72] reported that 6 wk of Ustukhuddus Alavi improved working memory and sustained attention in Iranian adolescents, though no effects on information processing speed were found.

##### Long-chain PUFAs

Ten RCTs examined the effects of O3 long-chain PUFA (LCPUFA) interventions on adolescent cognition, using fish oil or algae oil supplements, fatty fish meals, or walnuts. Most were conducted in high-income countries; 1 study was based in Mexico. Interventions ranged from 2 to 12 mo, with placebo or habitual diet as controls. Compliance and biomarker changes varied.

Attention improved in 3 of 8 studies, including those using fatty fish meals [12], walnuts [36], and fish oil supplements [37]. However, effects were often limited to subgroups with high compliance or compromised by control group differences in nutrient uptake. Several studies [35] found no behavioral improvements in attention despite biomarker increases, though neural activation increased during cognitive tasks. Four studies reported no effects on attention.

Information processing speed was assessed in 6 studies, 3 of which found significant improvements [37,38], including in malnourished adolescents and those consuming fatty fish meals. However, biomarker improvements did not consistently correlate with cognitive gains.

The effects on memory were mixed. Delayed verbal memory improved in 1 study [33], though findings were inconsistent across memory subtypes. One study [37] reported >70% of the fish oil group showed clinical improvement in working memory, but between-group differences were nonsignificant.

Some improvements in executive function were reported. Inhibitory control [37], verbal fluency, and mental flexibility [38] improved in isolated cases, but findings were inconsistent overall.

Three studies assessed IQ. Perceptual reasoning [37] and inductive reasoning [36] improved in participants with good compliance. No effects were seen on full-scale IQ [34]. One study found improvements in perceptual integration, with >70% of the active group achieving a clinically significant gain.

Academic performance was assessed in 4 studies; no significant effects were observed, partly due to ceiling effects in 1 case [12].

In summary, evidence linking O3 supplementation to cognitive or academic improvement in adolescents is inconsistent, though some benefits have been observed for attention, processing speed, and specific executive functions, particularly under conditions of high compliance or nutrient insufficiency. Effects are domain-specific and may depend on formulation, co-nutrient intake, and participant nutritional status.

##### Mixed grains

Chung et al. [41] conducted a 9-wk RCT in a Korean school dormitory comparing whole grain, bean, and walnut-based meals to a refined rice-based control diet. Delayed verbal memory improved more in the control group, whereas sustained attention declined only in the control group, suggesting a potential protective effect of the active diet. Plasma brain-derived neurotrophic factor levels declined in the control group but slightly increased in the active group, indicating possible effects on synaptic plasticity.

##### New Nordic Diet

A within-subjects RCT in Denmark assessed the effects of the NND on cognitive and academic performance [[42], [43], [44]]. Compared with a control period (home-packed meals), the NND period was associated with greater improvements in reading speed, and smaller increases in inattention and impulsiveness. No changes were observed in concentration, processing speed, or maths. O3 LCPUFA levels increased during the NND period. Effects varied by sex, parental education, and baseline reading ability. Confounding factors included classroom disruption, cooking participation, and extended lunch duration.

##### School breakfast programs

Three studies (2 RCTs, 1 non-RCT) examined the effects of government-funded SBPs on adolescent cognition and academic performance in low-SES settings in the United Kingdom and Peru. Intervention durations ranged from 12 mo to 3 y; controls were schools without SBP implementation.

Memory outcomes were mixed. Murphy et al.’s [73] RCT found no effect on verbal memory >12 mo, though high protocol noncompliance limited interpretation. Cueto and Chinen [13] found that in lower-SES, mixed-grade Peruvian schools, visual memory improved in schools implementing SBPs, but no effect was seen in higher-performing full-grade schools.

Information processing speed improved in 1 RCT after 3 mo [74], though widespread protocol violations and crossover contamination limited follow-up analysis. No differences were found in the Peruvian study.

Academic performance, assessed only in [13], showed improved math and reading scores in mixed-grade SBP schools, but poorer outcomes in full-grade SBP schools compared with controls.

In summary, findings suggest potential benefits of SBPs on select outcomes, but are undermined by noncompliance, design limitations, and inconsistent baseline conditions. Furthermore, high-quality, controlled evaluations are needed to clarify SBP effects on cognitive and academic outcomes.

##### Multinutrient interventions

Eleven studies (10 RCTs, 1 non-RCT) assessed multinutrient interventions, including multivitamin and mineral (MVM) supplements, fortified milk, and iron-based combinations (e.g., with zinc, vitamin C, or folic acid), across 2–12 mo. Most trials used placebo controls and were conducted in both high- and low-income countries.

Non-verbal IQ improved in 3 studies following MVM or iron supplementation, particularly in participants with pre-existing deficiencies [47,50,53]. Two studies found no effect despite biomarker improvements. Verbal IQ improved in 1 trial using an iron- and vitamin C-based fortified snack in anemic adolescents [46], but not in others.

Memory outcomes were mixed. Verbal memory showed no effect in 2 studies. Visual memory improved in anemic adolescents after iron or iron–folic acid supplementation [11,51], whereas 1 study found a decline attributed to chance. Working memory improved in 2 studies—one using fortified milk with O3s, and one using iron–folate—but not under low-frequency dosing. Spatial and semantic memory showed no change.

Executive function findings were variable. Planning improved with iron–folate supplementation [51], but inhibitory control did not. Attention improved in 2 studies [45,51], but not in 1 iron-zinc trial. Information processing speed improved in 2 studies using MVMs or iron–zinc [11,55], but not in others.

Academic performance was measured in 3 studies. Two MVM trials reported no effect. In 1 case, this may be due to suboptimal iron dosing [48]. However, fortified milk improved academic grades in adolescents with baseline vitamin B2 deficiency [54].

In summary, multinutrient interventions showed mixed effects across cognitive domains, with nonverbal IQ, visual memory, and attention appearing most sensitive to change, particularly in nutritionally vulnerable populations. However, variability in intervention content, dosing, duration, and baseline nutritional status limits firm conclusions. Evidence for improvement in academic performance is currently inconsistent.

### Prospective studies beginning during infancy

#### Study characteristics

Of the 13 studies identified, 11 measured cognition during adolescence [[76], [77], [78], [79], [80], [82], [83], [84], [85], [86], [87]] and 3 measured academic achievement during adolescence [[75], [81], [86]]. The mediating effect of brain morphology was also examined by [77]. Ten studies were conducted in countries with developed economies (Australia [*n* = 3], United Kingdom [*n* = 5], China, and the Netherlands), and 3 in countries with developing economies (Chile and Costa Rica [*n* = 2]). Three studies examined the effects of IDA during infancy on later development [85,86,87]. Ten studies assessed dietary intake from 6 to 36 mo of age and cognitive/academic performance from 8 to 17 y of age, using a 24-h food recall- or Food Frequency Questionnaire. Six studies created a dietary quality score or index [[76], [77], [79], [80], [81], [84]], 5 studies identified dietary patterns using principal component analysis or factor analysis [77,78,82,83,75], and 4 studies examined specific foods or food groups [79,80,84,81].

#### Risk of bias

Ten studies showed a low RoB. The remaining 3 studies did not meet the low RoB cut-off due to the use of 24-h food recall questionnaires. All studies statistically controlled for the influence of key confounding factors; however, only 2 studies controlled for maternal IQ [76,83], 2 studies controlled for previous diet [76,75], and 1 study controlled for both previous and current diet [78].

#### Association between diet during infancy and cognitive and academic performance during adolescence

##### General diet quality

Seven longitudinal studies examined the relationship between infant diet (birth to 3 y) and later intelligence, using large cohort datasets from Australia, the United Kingdom [Avon Longitudinal Study of Parents and Children (ALSPAC)], The Netherlands, and China. Most studies adjusted for 8–13 covariates, including SES and maternal education, though only 2 adjusted for maternal IQ.

Across studies, better diet quality during infancy—characterized by higher intake of fruits, vegetables, dairy, and whole grains, and lower intake of processed or sugary foods—was consistently associated with higher verbal and full-scale IQ scores during childhood or adolescence. Evidence for nonverbal IQ was less consistent. Positive associations were typically strongest for diet at age 1, with weaker or null findings for diet at ages 2 and 3 [76,78,84,79].

Specific nutrient-rich foods were also implicated: dairy and fruit intake at 1 y predicted higher IQ scores in multiple studies. Adherence to complementary feeding guidelines (e.g., introduction of iron-rich and protein-rich foods between 6 and 12 mo) was also associated with better verbal and full-scale IQ in Zhu et al. [84]. Processed food intake at 6–24 mo was negatively associated with IQ scores [82]. Although 1 ALSPAC study found a “snack” dietary pattern (finger-foods, biscuits) positively associated with IQ [78], this may reflect SES or parent–child interaction factors.

Trajectory analyses showed that a “healthy” dietary pattern across the first 2 y was weakly associated with IQ at age 8 [83], whereas “discretionary” and “traditional” dietary patterns were negatively associated with IQ at age 15.

No direct link was found between diet quality and IQ [77], but the authors reported that a processed dietary pattern at 1 y was negatively associated with white matter volume at age 10, which in turn mediated the relationship with IQ at age 13.

Only 2 studies explored other cognitive domains. Diet at 1 y was positively associated with information processing speed, working memory, and verbal comprehension [84,80], but no associations were found for spatial or visual memory, and executive function was not assessed in any study.

Two studies examined academic performance in adolescence. A “junk food” dietary pattern at age 3 [75] and lower diet quality scores at ages 1–3 [81] were negatively associated with math and literacy scores at ages 10–12, even after adjusting for multiple confounders, including earlier diet.

In summary, there is growing evidence that early diet quality, especially in the first year of life, predicts later intelligence and academic outcomes, with the strongest support for verbal IQ and literacy/math attainment. However, methodological limitations remain, including limited adjustment for maternal IQ, few analyses of other cognitive domains, and an over-reliance on dietary patterns over mechanistic or nutrient-specific analysis.

##### Iron

Three longitudinal studies examined the long-term effects of early IDA. In [85], infants treated for IDA had normal hemoglobin levels by 10 y yet continued to show poorer inhibitory control and altered event-related potential markers (longer N2 latency, reduced P300 amplitude) compared with iron-sufficient peers. Similarly, authors in [86] and [87] found that adolescents and young adults who were IDA in infancy had poorer attention, arithmetic, memory, and executive function despite iron repletion, suggesting that early IDA may have lasting neurocognitive effects, even when iron status normalizes.

### Prospective studies beginning during adolescence

#### Study characteristics

Of the 12 prospective studies identified, 4 measured cognitive performance [77,88,90,94], and 9 measured academic performance [90,89,[91], [92], [93],[95], [96], [97], [98]]. The mediating effect of brain morphology was examined [77]. Fish intake was assessed in 2 studies [88,89], adherence to the Mediterranean diet in 1 study [96], and general diet quality in 7 studies [77,[90], [91], [92],^94^,^93^,^95^]. Two studies examined the longitudinal effects of an SBP [97,98]. Ten studies were conducted in countries with developed economies [Sweden (*n* = 2), Canada (*n* = 3), United States (*n* = 2), Australia, Hawaii, and the Netherlands], and 2 in countries with developing economies (Jamaica and Lebanon).

#### Risk of bias

Two, 6, and 4 studies showed a low, moderate, and high RoB, respectively (Supplemental Table 8). Most studies did not measure dietary intake using a validated food frequency questionnaire or control for the influence of key confounding factors including dietary intake at follow-up, pubertal status, other relevant aspects of diet, or sleep quality. Three studies used self-report measures of academic achievement, and nonresponse bias was an issue in almost all studies. One SBP study did not control any confounding factors [97], and one did not check for baseline differences between groups [98].

#### Association between diet during early adolescence and cognitive and academic performance during late adolescence

##### General diet quality

Twelve longitudinal studies explored links between diet during mid-adolescence (8–14 y) and later cognitive or academic performance.

In Mou et al. [77], no association was found between overall diet quality or patterns at age 8 and IQ at age 13. However, a “whole grains, soft fats, and dairy” pattern was associated with greater brain volume at age 10, which mediated later IQ. In contrast, Nyaradi et al. [94] found that a “Western” dietary pattern at age 14 was negatively associated with information processing speed and spatial memory at 17, but not other domains. Poorer performance was linked to higher intakes of fried potatoes, red meat, and potato chips, and lower intakes of leafy greens and fruit.

In Purtell and Gershoff [95], daily fast food consumption at ages 10–11 predicted poorer maths, literacy, and science grades at ages 13–14, even after adjusting for other dietary components.

Evidence on fruit and vegetable intake was mixed. One study [93] found a negative association with academic performance, though only gender and ethnicity were adjusted for. Others reported no association [91], potentially due to low rates of recommended intake. However, Dubuc et al. [90] found that increased fruit and vegetable consumption over 3 y predicted higher academic grades in males.

Meeting dietary guidelines for meat or milk intake consistently predicted better maths and reading achievement in 2 studies [91,92]. Associations held, even after controlling for multiple lifestyle and demographic factors, although SES was approximated using adolescent spending money.

In summary, frequent consumption of processed and fast foods in adolescence appears consistently linked to poorer cognitive and academic outcomes, whereas evidence for fruit and vegetable intake is more variable. Some associations may be masked by low adherence to dietary guidelines or insufficient adjustment for socioeconomic and confounding factors.

##### Fish intake

Two studies found that frequent fish consumption at age 15 was associated with better cognitive and academic outcomes. Males who ate fish more than once per week had higher IQ and visuospatial scores at 18 [88], and weekly fish intake predicted higher academic grades 1 y later [89]. However, neither study distinguished between fatty and lean fish, nor adjusted for other dietary factors that could confound results.

##### Mediterranean diet

Hayek et al. [96] found that improved adherence to the Mediterranean diet was positively associated with academic grades after 6 and 12 mo, even after controlling for 7 sociodemographic factors. No associations were found with BMI, physical activity, alcohol, or smoking. However, grades were self-reported, raising the possibility of respondent bias.

##### School breakfast programs

Two studies found positive effects of SBPs on academic performance. In undernourished Jamaican adolescents, daily breakfast consumption led to improved arithmetic scores over 3 mo, with no changes in spelling or reading [98]. In low-SES schools (United States), increased SBP participation over 4 mo was linked to a +0.3 grade improvement in maths, whereas decreased participation was linked to a decline [97]. No associations were found for other academic subjects.

## Discussion

### Randomized and non-RCTs

This review summarized the findings from 48 controlled trials that examined the effects of different nutrients, food groups, and dietary patterns on cognitive and academic performance in adolescents, including iron, iodine, vitamin D, choline, polyphenols, O3 fatty acids, multiple micronutrients, whole grains, the NND, and SBPs. Overall, there was limited evidence that any dietary intervention consistently and unequivocally benefited cognitive function or academic achievement during adolescence. However, this is perhaps unsurprising given the methodological heterogeneity between studies, including the type of intervention (foods or supplements), dosage, sample size, length of intervention, SES, measures of cognitive and academic performance, biological sex, age, stage of puberty, and baseline nutritional, cognitive, and academic status.

There was some evidence to suggest that iron treatment may benefit verbal memory, full-scale IQ, and nonverbal IQ in adolescents, especially in those with poorer iron/hemoglobin status at baseline. In contrast, a recent systematic review concluded that iron treatment during adolescence may be beneficial for improving school performance and attention [99]. Our conclusions differ as we defined adolescence using a narrower age range; hence, slightly different studies were reviewed. Most studies consisted of adolescents with mixed iron/hemoglobin status, ranging from normal status to ID or IDA. This complicates the interpretation of findings, especially as many studies did not examine whether pre-existing iron/hemoglobin status moderates the effects of iron treatment. Folic acid, zinc, and vitamin A, B12, D, and C deficiencies also tend to co-occur with iron deficiencies [[100], [101], [102]], which could affect intestinal absorption of iron [103,104] and mask an iron-induced improvement in cognitive or academic performance if left undiagnosed.

Iodine is required for the synthesis of thyroid hormones which are essential for neurogenesis, synaptogenesis, myelination, and neurotransmission [105]. The detrimental cognitive effects of inadequate iodine intake during infancy and in utero are widely established, with cretinism being the most serious consequence [106,107]. In comparison, little is known about the effects of iodine treatment during adolescence. Two well-controlled studies showed that treatment with iodine improved nonverbal IQ in mildly deficient adolescents [66] and both nonverbal IQ and information processing speed in moderately-to-severely deficient adolescents [69]. These findings suggest that iodine treatment during adolescence may produce a “catch-up” effect, which has also previously been reported in children [108]. However, the age at which participants became iodine deficient is unknown, and there may be an age threshold whereby improvements are no longer possible. Given that mild iodine deficiency is a re-emerging issue in developed countries, including the United Kingdom, United States, and Australia, due to changes in food manufacturing practices and dietary habits [109,110], further research is warranted.

There was no consistent evidence that O3 LCPUFA interventions (via supplements, fatty fish meals, or walnuts) improved cognitive or academic performance in adolescents. However, the execution of some studies was unsuccessful, which may have contributed to the absence of effect. For example, the Food2Learn study reported no improvements in 5 cognitive domains after 12 mo of fish oil supplementation [39]. However, 20% of participants withdrew from the study, 32% of participants in the active group stopped taking the supplements, and O3 index (O3I) only increased by 1.15% after 12 mo in the active group, remaining within the suboptimal range (<5%). A systematic review of RCTs involving children and adolescents found that cognitive improvements after O3 LCPUFA supplementation were more likely to occur in studies where participants O3I increased to >6% postintervention [111]. Consistent with this finding, 2 trials that reported improvements in cognitive function after fatty fish intake also reported a significant increase in O3I above 6% [12,32,38], suggesting that future research may benefit from targeting O3I.

There was some suggestion that MVM supplementation during adolescence may improve nonverbal IQ. This is consistent with the results of a meta-analysis which reported a trend towards better nonverbal IQ, but not verbal IQ, in children and adolescents (5–16 y) after MVM supplementation [112]. We were unable to obtain 4 full-text articles that administered MVM supplements [[27], [28], [29], [30]], potentially influencing our conclusions. Very few studies examined the effects of vitamin D [70], choline [31], whole grains [41], or polyphenols [71,72] on cognitive function during adolescence. Research into the effects of supplementation with vitamin D and B12 is important as nutritional deficiencies are common during adolescence [58,[113], [114], [115]].

Three studies examined the effects of SBPs in adolescents. Although improvements in cognitive and academic performance were reported in 2 studies [13,74], all 3 studies were confounded by major methodological limitations including contamination between intervention groups, no baseline measures, unclear timings of cognitive/academic assessments, and no standardization of breakfast composition between schools. The impact of SBPs on cognitive and academic performance in children and adolescents has been reviewed extensively, and it is widely agreed that SBPs are particularly difficult to implement and monitor [[116], [117], [118], [119], [120]].

A major limitation of the interventions conducted to date is that they have often isolated single or multiple ‘known’ nutrients. However, foods contain many thousands of chemicals beyond the well-known nutrients such as vitamins, minerals, proteins, carbohydrates, and fats. Many of these compounds, like polyphenols, flavonoids, and terpenes, have known health benefits, but countless others have not yet been identified or studied [121]. Furthermore, foods contain complex mixtures of compounds that can act in synergy with each other. The combined effects of these compounds can differ from the effects of each one in isolation [122]. Therefore, although logistically much more challenging, studies considering whole foods and/or dietary patterns may be more profitable.

### Neurodevelopmental mechanisms

When considering the potential impact of diet on adolescent brain function, it is important to evaluate the plausibility of the underlying neurobiological mechanisms [121]. Taken together, the mixed but suggestive findings for iron, iodine, O3 LCPUFA, and multimicronutrient interventions are broadly consistent with current understanding of adolescent brain development. This period is characterized by protracted myelination, synaptic pruning and maturation of frontoparietal networks that support executive functions, working memory, and academic skills (e.g., mathematics and reading comprehension). Nutrients that contribute to myelination, neurotransmitter synthesis, and energy metabolism, such as iron, iodine, O3 LCPUFA and B-vitamins, are therefore theoretically well placed to influence cognitive trajectories, particularly when deficiencies arise during sensitive developmental windows. The apparent domain specificity of some effects (e.g., verbal compared with nonverbal IQ, or executive functions compared with global cognition) may reflect the different regional and temporal profiles of neurodevelopment, as well as the interaction between biological potential and enriched learning environments.

### Sources of heterogeneity

Given what is known about adolescent brain development, and the biological plausibility that diet can influence myelination, synaptic pruning, and frontoparietal network maturation, the limited and sometimes contradictory effects observed across interventions are unlikely to reflect a complete absence of nutritional influence on adolescent cognition. Rather, they appear to arise from several sources of heterogeneity in populations, interventions, and methods.

First, baseline nutritional status differs markedly between studies. For iron and iodine, benefits are more frequently observed in samples with evidence of deficiency or marginal status, whereas trials in largely replete adolescents tend to show smaller or null effects. Many studies included adolescents with a mixture of normal status, ID and IDA, and did not formally test baseline status as an effect modifier, making it difficult to identify who benefits most. A similar issue arises for O3 LCPUFA, where background intake of oily fish and fortified foods varies widely between settings.

Second, there is substantial variation in dose and duration of intervention. Micronutrient trials differ in the amount and formulation used (single compared with multiple micronutrients, fortified foods compared with supplements) and in the length of exposure. Cognitive and academic outcomes are unlikely to respond uniformly to short-term compared with longer-term changes in intake. For O3 LCPUFA, for example, trials that produced minimal changes in the O3I reported no cognitive benefits, whereas trials in which the O3I exceeded 6% were more likely to show improvements, suggesting that the intensity of biological change matters.

Third, the timing of exposure is heterogeneous. Some prospective studies examine diet during infancy and early childhood and follow participants into adolescence, whereas others focus on diet assessed during early adolescence itself. These windows correspond to different phases of brain and educational development, and it is plausible that early deficiencies have more persistent effects on global or verbal abilities, whereas later dietary patterns may influence executive functions and current school performance. Aggregating across these developmental periods without distinction may obscure age-sensitive effects.

Fourth, interventions target a wide range of cognitive domains and academic outcomes. Outcomes include global IQ, domain-specific scores (verbal, nonverbal, working memory), executive functions, attention, and various markers of academic achievement. Where effects are detected, they are often domain-specific. At the same time, there is considerable variation in the tests used and in follow-up intervals, which reduces comparability between studies.

Fifth, context of delivery and study setting likely influence both effect sizes and their stability. School-based programs depend on implementation fidelity, teacher engagement, and wider food environments, whereas clinical or community settings may offer more control but less ecological validity for day-to-day adolescent eating patterns. Socioeconomic context and educational systems also differ between countries, particularly between high-income countries and settings with a higher burden of undernutrition and food insecurity.

Finally, heterogeneity is amplified by methodological differences. Studies vary in RoB, adjustment for confounding, and handling of attrition and missing data. As highlighted in Neurodevelopmental mechanisms and Sources of heterogeneity sections, several longitudinal studies did not fully account for maternal IQ, home learning environment or current diet, and included relatively few covariates, leaving substantial scope for residual confounding. Differences in analytic approaches (e.g., intention-to-treat compared with per-protocol analyses) may further contribute to divergent findings.

These factors together suggest that the apparent inconsistency in the literature should not be interpreted as evidence that diet is unimportant for adolescent cognitive or academic outcomes. Instead, the impact of diet appears contingent on who is studied, when in development exposure occurs, what is delivered (and for how long), which domains are assessed, and the context in which interventions are implemented.

### Prospective studies beginning during infancy

To the best of our knowledge, this is the first systematic review to summarize the results of prospective studies examining the association between diet during infancy and cognitive and academic performance during adolescence. Generally, studies showed that an unhealthy dietary pattern (e.g., higher intake of soft drinks, processed foods, and salty snacks, and lower intake of whole grains, fruits, dairy, and vegetables) during the first 3 y of life may negatively affect intelligence during adolescence. There was also evidence to suggest that suboptimal nutrition during the first year of life may be particularly detrimental for long-term intelligence, potentially reflecting the drastic changes in brain structure and function during this period, such as a rapid increase in glucose metabolism in the frontal cortex [123], and myelination in the frontal, occipital, and parietal lobes [124]. Total brain volume also increases by ∼101% in the first year of life, compared with 15% in the second year of life [125]. The first year of life may therefore represent a more pronounced period of vulnerability to nutritional deficiencies or inadequacies.

Verbal IQ also appeared to be more susceptible to the effects of early diet than nonverbal and performance IQ, which contrasts with the MVM supplementation literature discussed in Randomized and nonrandomized controlled trials section. Nonverbal intelligence is a reflection of biological potential, whereas verbal IQ reflects one's knowledge, experience, and environment [126]. Consequently, improvements in nonverbal intelligence would be expected after diet-induced improvements in biology [127]. This raises the question of whether the association between diet during infancy and verbal IQ during adolescence reflects uncontrolled confounding from other environmental factors [82]. According to Benton [127], an increase in verbal intelligence would be expected if biological potential interacts with a stimulating home and school environment over a longer period of time.

Both studies that measured other cognitive domains reported that the consumption of healthier foods or a healthier dietary pattern during infancy was associated with better cognitive function during adolescence [84,80]. Two studies measured academic achievement during adolescence, both of which reported that an unhealthier dietary pattern during infancy was associated with poorer academic achievement during adolescence [75,81]. Although household income during infancy was included as a covariate in both studies, the confounding effects of parental styles and attitudes towards health and education cannot be ruled out, as well as accessibility of books and computers during childhood [[128], [129], [130]].

Despite the promising findings, there are several methodological caveats that should be discussed. Nonresponse bias was an issue in all studies. Generally, those who were lost to follow-up were more likely to be ethnic minorities and have a low birthweight, and less likely to have mothers who are well-educated, health conscious, and middle-to-upper class. Because dietary patterns are specific to geographical regions and populations [131], studies conducted in countries with developed economies cannot be generalized to countries with developing economies, particularly those experiencing a double burden of malnutrition with coexisting undernutrition and rising rates of overweight and obesity [131]. In all studies, the strength of the association between diet during infancy and functioning during adolescence was significantly reduced after adjusting for confounding factors, which raises the possibility that residual confounding remains [83,132]. The number of confounding factors identified varied between studies, ranging from 8 [81] to 19 factors [75]. All studies included maternal education, biological sex, and an index of SES as covariates, 9 studies included maternal age as a covariate, and 7 included a measure of cognitive/language stimulation at home. The confounding effect of maternal IQ was only considered in 2 studies [76,83], which is problematic: studies have shown that the association between breastfeeding and later cognitive development in infancy is largely the result of confounding by maternal IQ [133,134]. Despite being closely related, maternal education is an inadequate surrogate of maternal IQ [78,133]. Other confounding factors that are important but were seldom considered include current diet [78] and diet during childhood [76,78,75]. Using the ALSPAC cohort, Northstone et al. [78] showed that dietary patterns were similar between 4 and 7 y, but changed significantly between 3 to 4 and 7 to 9 y, highlighting the importance of repeated assessments of diet.

Three studies examined the long-term effects of IDA or ID during infancy. Algarin et al. [85] reported that executive functioning at 10 y of age was poorer in those who were treated for IDA during infancy than controls. Positive ERP component [∼300 ms poststimulus (P300)] amplitudes were also smaller in those who were IDA, which may reflect long-lasting dysfunction of the dopamine system. Cognitive and academic performance at 11–14 y, and executive functioning at 19 y, was poorer in those whose ID did not resolve after 3 mo of iron treatment during infancy [86,87]. A limitation of both studies is that prenatal iron and hemoglobin levels were not determined, nor is it clear when levels normalized during infancy/early childhood. Therefore, the importance of iron status during the first 3 y of life for functioning during adolescence remains unclear. Although infants in these studies were otherwise healthy and growing normally, undernourished infants in developing countries are likely to have other comorbid nutritional deficiencies, poorer healthcare, insufficient stimulation at home, lower quality schooling, and parents with lower levels of education, all of which can contribute to cognitive dysfunction and academic underachievement [[124], [135], [136]].

### Prospective studies beginning during adolescence

Twelve studies examined the association between diet during early adolescence and cognitive and/or academic performance during later adolescence. A higher intake of unhealthy foods (e.g., soft drinks, fast food, or crisps) and/or lower intake of healthy foods (e.g., leafy greens, dairy, fish, fruits, or vegetables) during early adolescence may be associated with poorer academic and cognitive performance in later adolescence. However, it is important to note that most of these studies showed either a moderate or high RoB. Furthermore, most studies only measured 1 aspect of diet, including fast food intake [95], fruit and vegetable intake [93], or fish intake [88,89]; an approach which fails to capture the complexity of diet and interaction between nutrients [122,137]. Other aspects of diet that covary with fish, junk food, or fruit and vegetables consumption were rarely included as covariates.

One study examined the association between adherence to the Mediterranean diet and academic grades. Hayek et al. [96] reported that increased adherence to the Mediterranean diet after 6 and 12 mo was associated with an increase in self-reported academic achievement in Lebanese adolescents. Two studies examined the longitudinal effects of implementing SBPs in low SES schools. One reported an improvement in mathematics grades but not science, social studies, and reading grades [97], and the other reported improvements in performance on an arithmetic test but not a spelling and reading test [98]. Because there was a negative association between SBP participation and school tardiness and absence after 4 mo in [97], improvements in mathematic grades may be due to an increase in schooling exposure rather than a nutritional effect per se.

Several other limitations should be considered when interpreting the findings. Residual confounding is an issue within this literature, as 9 studies included <8 covariates. No studies included current tobacco, alcohol, or recreational drug use as covariates, all of which are associated with poorer academic achievement [[138], [139], [140]]. Intake of specific foods was assessed using 1 question in 4 studies [88,89,93,95]. Additionally, across the included studies, “Mediterranean” and “unhealthy/processed” dietary patterns were not operationalized in a uniform way. Some studies used predefined adherence indices derived from food-frequency questionnaires, whereas others identified patterns using data-driven methods (e.g., principal components or cluster analysis) (Table 2). These approaches are common in the literature but variability in scoring and labeling limits direct comparability of effect estimates between studies.

### Limitations

There are several limitations that warrant attention. The quality of RCTs was generally poor, with studies either showing some concerns of bias or a high RoB. There is considerable methodological heterogeneity between studies that administered similar dietary interventions, making it difficult to draw any firm conclusions from the existing literature. Adolescents aged between 8 and 19 y were included in this review. The use of age to define adolescence fails to capture the abundance of puberty-induced changes in biology that may interact with the effects of diet on cognition and academic achievement. This is particularly relevant here because the eligible age range was extended to 8–19 y, meaning that some included participants were younger than the conventional adolescent range and may differ developmentally from older adolescents. Although we intended to explore the potential moderating effect of pubertal status, this was not possible due to a lack of literature. Variability in puberty stage within and between studies may have complicated the interpretation of findings. For example, 2 studies excluded females who had attained menarche before or during the study [51,31]. Academic achievement was measured in different ways, including standardized and unstandardized tests, school records, and self-reported academic grades. The latter can be inaccurate, especially in students who are underachieving [141]. There was also significant heterogeneity in cognitive tests used, which is a known issue affecting the comparability of cognitive nutrition studies [142]. The search was restricted to English-language publications for pragmatic reasons, which may have reduced capture of relevant studies, particularly from low- and middle-income countries.

### Recommendations for future research

Future RCTs should stratify by pubertal stage and assess O3I or micronutrient biomarkers to confirm nutrient uptake. Longitudinal studies should follow adolescents across multiple developmental points, with repeated dietary assessments and robust confounding control. More studies are needed on academic achievement as a primary outcome, given its relevance to long-term life chances. Finally, the possibility that adolescent nutrition influences cognitive aging remains largely unexplored and warrants investigation.

**FIGURE 2:**
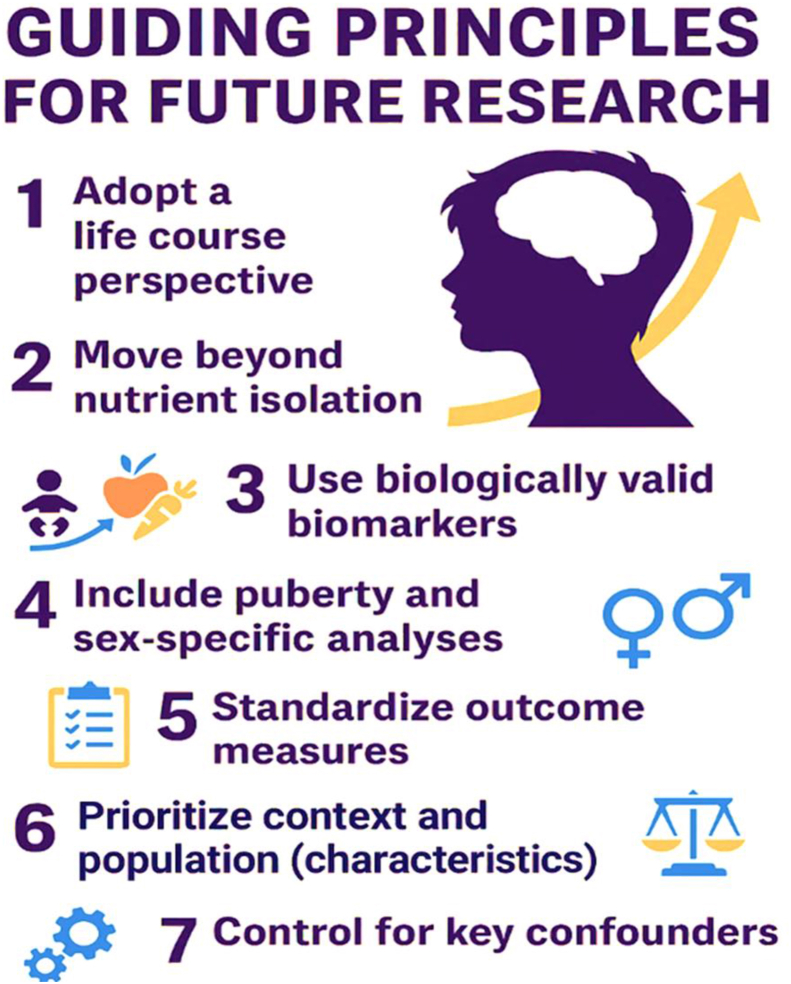
Seven guiding principles derived from the current review to inform future research on adolescent diet and cognition. These include life-course considerations, dietary complexity, biomarker validation, sex/puberty differentiation, standardized outcomes, equity/context sensitivity, and rigorous confounder control.

To advance the field, we propose the following guiding principles for future research at the intersection of diet, cognition, and academic performance during adolescence (Figure 2):

1. *Adopt a life-course perspective.* Research should recognize that cognitive outcomes in adolescence may reflect cumulative exposures starting in infancy. Future studies should track diet across multiple developmental windows and assess its long-term effects.
2. *Move beyond nutrient isolation.* Although single-nutrient trials are mechanistically valuable, real-world diets are complex. Studies should prioritize whole-diet or dietary pattern approaches that capture nutrient interactions and synergies.
3. *Use biologically valid biomarkers.* Intervention trials should include biomarkers (e.g., O3I, ferritin) to confirm physiological uptake, enabling a mechanistic understanding of null or positive effects.
4. *Include puberty and sex-specific analyses.* Given the profound hormonal and neural changes during adolescence, studies should assess pubertal status and explore sex-specific responses to dietary interventions.
5. *Standardize outcome measures.* Cognitive and academic assessments should be harmonized across studies to improve comparability. Validated and age-appropriate tools should be used, ideally aligned with international standards.
6. *Prioritize context and population characteristics.* Research should consider SES, food access, school food policy, and cultural eating patterns. Greater inclusion of diverse and underserved populations is essential for generalizable findings.
7. *Control for key confounders.* Maternal IQ, home learning environment, substance use, and ongoing diet must be routinely controlled for in longitudinal designs to minimize residual confounding.

In parallel with methodological improvements, there is a need to align research outputs more closely with policy and practice. Standardized evaluation frameworks for school feeding and snack programs, including agreed core cognitive and academic outcomes and minimum follow-up periods, would facilitate comparison across interventions. Targeted micronutrient supplementation strategies informed by baseline status, rather than universal supplementation in largely replete populations, may also yield clearer signals of benefit while avoiding unnecessary exposure. Finally, greater use of harmonized definitions of dietary patterns (e.g., Mediterranean-type diets or ultraprocessed “Western” patterns) would improve both synthesis and translation into dietary guidelines and school-based nutrition policies.

In conclusion, more research is needed to determine whether adolescence represents a window of opportunity for optimizing cognitive development via nutritional interventions. The lack of methodological consistency between studies administering similar dietary interventions makes it difficult to discern the effect of specific nutrients on cognition and academic achievement. RCTs investigating the effects of vitamin D, choline, polyphenols, and whole grains were scarce, and although some positive findings were reported, the evidence base remains limited. Prospective studies provided evidence that unhealthy dietary patterns during infancy, especially the first year of life, may negatively affect intelligence during adolescence. Future studies would benefit from specifically recruiting from and separately analyzing early and late adolescent populations and should implement more rigorous control of confounding factors. If replicated in well-controlled studies, these findings would have important implications for the development of dietary guidelines for adolescents globally.

## Author contributions

The authors’ responsibilities were as follows – HAY, CMG: contributed to conceptualization and project administration; HAY, AM, CMG: contributed to methodology; CMG: contributed to writing – original draft; HAY, ARG, CMG: contributed to writing – review and editing; AB, HAY: contributed to supervision and funding acquisition; and all authors: read and approved the final article for submission.

## Data availability

Resources used in the production of this manuscript will be made available on the Open Science Framework registry (https://osf.io/c6xze).

## Funding

HAY received support for this work via grant IAFNS-SWANSEAU-20230111.

## Declaration of generative AI and AI-assisted technologies in the writing process

During the preparation of this work the authors used ChatGPT (GPT‑5.1; OpenAI) to produce the initial draft of Figure 2. After using this tool, the authors reviewed and verified the figure, and take full responsibility for the content of the publication.

## Conflict of interest

This work was supported by the Institute for the Advancement of Food and Nutrition Sciences (IAFNS) via grant IAFNS-SWANSEAU-20230111. IAFNS is a nonprofit science organization that pools funding from industry and advances science through the in-kind and financial contributions from private and public sector members.

## References

[1] Young H.A., Cousins A.L., Byrd-Bredbenner C., Benton D., Gershon R.C., Ghirardelli A. (2024). Alignment of consumers' expected brain benefits from food and supplements with measurable cognitive performance tests. Nutrients.

[2] Cusick S.E., Georgieff M.K. (2016). The role of nutrition in brain development: the golden opportunity of the “first 1000 days”. J. Pediatr..

[3] Morris S.S., Cogill B., Uauy R. (2008). Maternal and Child Undernutrition Study Group, Effective international action against undernutrition: why has it proven so difficult and what can be done to accelerate progress?. Lancet.

[4] Blakemore S.-J., Burnett S., Dahl R.E. (2010). The role of puberty in the developing adolescent brain. Hum. Brain Mapp..

[5] Lenroot R.K., Giedd J.N. (2006). Brain development in children and adolescents: insights from anatomical magnetic resonance imaging. Neurosci. Biobehav. Rev..

[6] López-Gil J.F., Martínez-Vizcaíno V., Amaro-Gahete F.J., Medrano M., Pascual-Morena C., Álvarez-Bueno C. (2023). Nut consumption and academic performance among adolescents: the EHDLA study. Eur. J. Nutr..

[7] Peng P., Kievit R.A. (2020). The development of academic achievement and cognitive abilities: a bidirectional perspective. Child Dev. Perspect..

[8] Moore Heslin A., McNulty B. (2023). Adolescent nutrition and health: characteristics, risk factors and opportunities of an overlooked life stage. Proc. Nutr. Soc..

[9] Norris S.A., Frongillo E.A., Black M.M., Dong Y., Fall C., Lampl M. (2022). Nutrition in adolescent growth and development. Lancet.

[10] Bruner A.B., Joffe A., Duggan A.K., Casella J.F., Brandt J. (1996). Randomised study of cognitive effects of iron supplementation in non-anaemic iron-deficient adolescent girls. Lancet.

[11] Chellappa A.R., Karunanidhi S. (2012). Paper presented at the International Conference on Nutrition and Food Sciences IPCBEE.

[12] Handeland K., Oyen J., Skotheim S., Graff I.E., Baste V., Kjellevold M. (2017). Fatty fish intake and attention performance in 14-15 year old adolescents: FINS-TEENS—a randomized controlled trial. Nutr. J..

[13] Cueto S., Chinen M. (2008). Educational impact of a school breakfast programme in rural Peru. Int. J. Educ. Dev..

[14] Georgieff M.K. (2017). Iron assessment to protect the developing brain. Am. J. Clin. Nutr..

[15] Page M.J., McKenzie J.E., Bossuyt P.M., Boutron I., Hoffmann T.C., Mulrow C.D. (2021). The PRISMA 2020 statement: an updated guideline for reporting systematic reviews. Syst. Rev..

[16] Tia A., Konan A.G., Hauser J., Ndri K.Y., Ciclet O., Esso L.E. (2025). A cross-sectional study of the relationship between dietary micronutrient intake, cognition and academic performance among school-aged children in Taabo, Côte d’Ivoire. Nutrients.

[17] García-Pérez-de-Sevilla G., Zapata-Lamana R. (2025). Adherence to the Mediterranean diet and its association with cognitive function in children and adolescents: a systematic review of observational studies. Children (Basel).

[18] Coombes J.P., Murphy M., Russell A., Turner A., Pallan M. (2025). Effect of diet on cognition, mental health and wellbeing among adolescents: protocol for a systematic review. BMJ Open.

[19] Nikalansooriya A., Waidyarathna G., Kaththiriarachchi L.S., Chandrasekara A. (2025). Role of nutrition in cognitive development and academic performance during adolescence: a comprehensive review. Cureus.

[20] Manidis A., Ayala-Aldana N., Bernardo-Castro S., Pinar-Martí A., Galkina P., Fernández-Barrés S., Ramirez-Carrasco P., Lamuela-Raventós R.M., Papandreou C., Julvez J. (2026). Dietary patterns and neuropsychological function in adolescents: a cross-sectional and longitudinal study. BMC Med.

[21] Fiani D., Chahine S., Zaboube M., Solmi M., Powers J.M., Calarge C. (2025). Psychiatric and cognitive outcomes of iron supplementation in non-anemic children, adolescents, and menstruating adults: a meta-analysis and systematic review. Neurosci. Biobehav. Rev..

[22] Tucker J.E., Brennan A.M., Benton D., Young H.A. (2025). A recipe for resilience: a systematic review of diet and adolescent mental health. Nutrients.

[23] Singh J.A., Siddiqi M., Parameshwar P., Chandra-Mouli V. (2019). World Health Organization guidance on ethical considerations in planning and reviewing research studies on sexual and reproductive health in adolescents. J. Adolesc. Health.

[24] Jager C.A., Dye L., Bruin E.A., Butler L., Fletcher J., Lamport D.J. (2014). Criteria for validation and selection of cognitive tests for investigating the effects of foods and nutrients. Nutr. Rev..

[25] United Nations (2023). World Economic Situation and Prospects 2023.

[26] Sterne J.A.C., Savović J., Page M.J., Elbers R.G., Blencowe N.S., Boutron I. (2019). RoB 2: a revised tool for assessing risk of bias in randomised trials. BMJ.

[27] Benton D., Buts J. (1990). Vitamin/mineral supplementation and non-verbal intelligence. Lancet.

[28] Benton D., Roberts G. (1988). Effect of vitamin and mineral supplementation on intelligence of a sample of schoolchildren. Lancet.

[29] Jinabhai C.C., Taylor M., Coutsoudis A., Coovadia H.M., Tomkins A.M., Sullivan K.R. (2001). A randomized controlled trial of the effect of antihelminthic treatment and micronutrient fortification on health status and school performance of rural primary school children. Ann. Trop. Paediatr..

[30] Crombie I., Todman J., Kennedy R., McNeill G., Menzies I. (1990). Effect of vitamin and mineral supplementation on verbal and non-verbal reasoning of schoolchildren. Lancet.

[31] O’Connor P.J., Chen X., Coheley L.M., Yu M., Laing E.M., Oshri A. (2022). The effects of 9 months of formulated whole-egg or milk powder food products as meal or snack replacements on executive function in preadolescents: a randomized, placebo-controlled trial. Am. J. Clin. Nutr..

[32] Handeland K., Skotheim S., Baste V., Graff I.E., Frøyland L., Lie Ø. (2018). The effects of fatty fish intake on adolescents’ nutritional status and associations with attention performance: results from the FINS-TEENS randomized controlled trial. Nutr. J..

[33] Kennedy D.O., Jackson P.A., Elliott J.M., Scholey A.B., Robertson B.C., Greer J. (2009). Cognitive and mood effects of 8 weeks' supplementation with 400 mg or 1000 mg of the omega-3 essential fatty acid docosahexaenoic acid (DHA) in healthy children aged 10-12 years, Nutr. Neurosci.

[34] Kirby A., Woodward A., Jackson S., Wang Y., Crawford M.A. (2010). A double-blind, placebo-controlled study investigating the effects of omega-3 supplementation in children aged 8–10 years from a mainstream school population. Res. Dev. Disabil..

[35] McNamara R.K., Able J., Jandacek R., Rider T., Tso P., Eliassen J.C. (2010). Docosahexaenoic acid supplementation increases prefrontal cortex activation during sustained attention in healthy boys: a placebo-controlled, dose-ranging, functional magnetic resonance imaging study. Am. J. Clin. Nutr..

[36] Pinar-Martí A., Gignac F., Fernández-Barrés S., Romaguera D., Sala-Vila A., Lázaro I. (2023). Effect of walnut consumption on neuropsychological development in healthy adolescents: a multi-school randomised controlled trial. EClinicalMedicine.

[37] Portillo-Reyes V., Pérez-García M., Loya-Méndez Y., Puente A.E. (2014). Clinical significance of neuropsychological improvement after supplementation with omega-3 in 8–12 years old malnourished Mexican children: a randomized, double-blind, placebo and treatment clinical trial. Res. Dev. Disabil..

[38] Teisen M.N., Vuholm S., Niclasen J., Aristizabal-Henao J.J., Stark K.D., Geertsen S.S. (2020). Effects of oily fish intake on cognitive and socioemotional function in healthy 8–9-year-old children: the FiSK Junior randomized trial. Am. J. Clin. Nutr..

[39] van der Wurff I.S.M., Von Schacky C., Bergeland T., Leontjevas R., Zeegers M.P., Jolles J. (2019). Effect of 1 year krill oil supplementation on cognitive achievement of Dutch adolescents: a double-blind randomized controlled trial. Nutrients.

[40] van der Wurff I.S.M., von Schacky C., Bergeland T., Zeegers M.P., Kirschner P.A., de Groot R.H.M. (2023). Krill oil supplementation's effect on school grades in typically developing adolescents. Prostaglandins Leukot. Essent. Fatty Acids.

[41] Chung Y.C., Park C.H., Kwon H.K., Park Y.M., Kim Y.S., Doo J.K. (2012). Improved cognitive performance following supplementation with a mixed-grain diet in high school students: a randomized controlled trial. Nutrition.

[42] Sørensen L.B., Dyssegaard C.B., Damsgaard C.T., Petersen R.A., Dalskov S.-M., Hjorth M.F. (2015). The effects of Nordic school meals on concentration and school performance in 8- to 11-year-old children in the OPUS School Meal Study: a cluster-randomised, controlled, cross-over trial. Br. J. Nutr..

[43] Sørensen L.B., Damsgaard C.T., Dalskov S.-M., Petersen R.A., Egelund N., Dyssegaard C.B. (2015). Diet-induced changes in iron and n-3 fatty acid status and associations with cognitive performance in 8–11-year-old Danish children: secondary analyses of the Optimal Well-Being, Development and Health for Danish Children through a Healthy New Nordic Diet School Meal Study. Br. J. Nutr..

[44] Sørensen L.B., Damsgaard C.T., Petersen R.A., Dalskov S.M., Hjorth M.F., Dyssegaard C.B. (2016). Differences in the effects of school meals on children’s cognitive performance according to gender, household education and baseline reading skills. Eur. J. Clin. Nutr..

[45] Haskell C.F., Scholey A.B., Jackson P.A., Elliott J.M., Defeyter M.A., Greer J. (2008). Cognitive and mood effects in healthy children during 12 weeks' supplementation with multi-vitamin/minerals. Br. J. Nutr..

[46] Kalaichelvi D., Santha N.J. (2021). Effectiveness of nutritional intervention in improving intelligence among adolescent girls. Int. J. Curr. Res..

[47] Lynn R., Harland E.P. (1998). A positive effect of iron supplementation on the IQS of iron deficient children. Pers. Individ. Differ..

[48] Perlman A.I., Worobey J., O'Sullivan Maillet J., Touger-Decker R., Hom D.L., Smith J.K. (2010). Multivitamin/mineral supplementation does not affect standardized assessment of academic performance in elementary school children. J. Am. Diet. Assoc..

[49] Petrova D., Bernabeu Litrán M.A., García-Mármol E., Rodríguez-Rodríguez M., Cueto-Martín B., López-Huertas E. (2019). Еffects of fortified milk on cognitive abilities in school-aged children: results from a randomized-controlled trial. Eur. J. Nutr..

[50] Schoenthaler S.J., Amos S.P., Eysenck H.J., Peritz E., Yudkin J. (1991). Controlled trial of vitamin-mineral supplementation: effects of intelligence and performance. Pers. Individ. Differ..

[51] Sen A., Kanani S.J. (2009). Impact of iron-folic acid supplementation on cognitive abilities of school girls in Vadodara. Indian Pediatr.

[52] Southon S., Wright A.J., Finglas P.M., Bailey A.L., Loughridge J.M., Walker A.D. (1994). Dietary intake and micronutrient status of adolescents: effect of vitamin and trace element supplementation on indices of status and performance in tests of verbal and non-verbal intelligence. Br. J. Nutr..

[53] Snowden W. (1997). Evidence from an analysis of 2000 errors and omissions made in IQ tests by a small sample of schoolchildren, undergoing vitamin and mineral supplementation, that speed of processing is an important factor in IQ performance. Pers. Individ. Differ..

[54] Wang X., Hui Z., Dai X., Terry P.D., Zhang Y., Ma M. (2017). Micronutrient-fortified milk and academic performance among Chinese middle school students: a cluster-randomized controlled trial. Nutrients.

[55] Buzina-Suboticanec K., Buzina R., Stavljenic A., Tadinac-Babic M., Juhovic-Markus V. (1998). Effects of iron supplementation on iron nutrition status and cognitive functions in children. Food Nutr. Bull..

[56] Devaki PB, Chandra RK, Geisser P (2009). Effects of oral iron(III) hydroxide polymaltose complex supplementation on hemoglobin increase, cognitive function, affective behavior and scholastic performance of adolescents with varying iron status: a single centre prospective placebo controlled study. Arzneimittel-Forschung.

[57] Kashyap P., Gopaldas T. (1987). Impact of hematinic supplementation on cognitive function in underprivileged school girls (8–15 yrs of age). Nutr. Res..

[58] Karkada S., Upadhya S., Upadhya S., Bhat G. (2019). Beneficial effects of ragi (finger millet) on hematological parameters, body mass index, and scholastic performance among anemic adolescent high-school girls (AHSG). Compr. Child Adolesc. Nurs..

[59] Lambert A.K., Knaggs K., Scragg R., Metcalf P., Schaaf D. (2002). Effects of iron treatment on cognitive performance and working memory in non-anaemic, iron-deficient girls. N. Z. J. Psychol..

[60] Pollitt E., Soemantri A.G., Yunis F., Scrimshaw N.S. (1985). Cognitive effects of iron-deficiency anaemia. Lancet.

[61] Pollitt E., Hathiral P., Kotchabhakdi N.J., Missell L., Valyasevi A. (1989). Iron deficiency and educational achievement in Thailand. Am. J. Clin. Nutr..

[62] Rezaeian A., Ghayour-Mobarhan M., Mazloum S.R., Yavari M., Jafari S.-A. (2014). Effects of iron supplementation twice a week on attention score and haematologic measures in female high school students. Singapore Med. J..

[63] Scott S.P., Murray-Kolb L.E., Wenger M.J., Udipi S.A., Ghugre P.S., Boy E. (2018). Cognitive performance in indian school-going adolescents is positively affected by consumption of iron-biofortified pearl millet: a 6-month randomized controlled efficacy trial. J. Nutr..

[64] Soemantri A.G., Pollitt E., Kim I. (1985). Iron deficiency anemia and educational achievement. Am. J. Clin. Nutr..

[65] Soemantri A.G. (1989). Preliminary findings on iron supplementation and learning achievement of rural Indonesian children. Am. J. Clin. Nutr..

[66] Gordon R.C., Rose M.C., Skeaff S.A., Gray A.R., Morgan K.M.D., Ruffman T. (2009). Iodine supplementation improves cognition in mildly iodine-deficient children. Am. J. Clin. Nutr..

[67] Huda S.N., Grantham-McGregor S.M., Tomkins A. (2001). Cognitive and motor functions of iodine-deficient but euthyroid children in Bangladesh do not benefit from iodized poppy seed oil (Lipiodol). J. Nutr..

[68] Isa Z.M., Alias I.Z., Kadir K.A., Ali O. (2000). Effect of iodized oil supplementation on thyroid hormone levels and mental performance among Orang Asli schoolchildren and pregnant mothers in an endemic goitre area in Peninsular Malaysia, Asia Pac. J. Clin. Nutr..

[69] Zimmermann M.B., Connolly K., Bozo M., Bridson J., Rohner F., Grimci L. (2006). Iodine supplementation improves cognition in iodine-deficient schoolchildren in Albania: a randomized, controlled, double-blind study. Am. J. Clin. Nutr..

[70] Grung B., Sandvik A.M., Hjelle K., Dahl L., Frøyland L., Nygård I. (2017). Linking vitamin D status, executive functioning and self-perceived mental health in adolescents through multivariate analysis: a randomized double-blind placebo control trial. Scand. J. Psychol..

[71] Nidich S.I., Morehead P., Nidich R.J., Sands D., Sharma H. (1993). The effect of the Maharishi Student Rasayana food supplement on non-verbal intelligence. Pers. Individ. Differ..

[72] Tefagh S., Mokaberinejad R., Shakiba M., Jafari M., Salehi M., Khayatkashani M. (2022). Effect of Ustukhuddus Alavi, a multi-herbal product, on the cognitive performance of adolescent female students. J. Ethnopharmacol..

[73] Murphy S., Moore G.F., Tapper K., Lynch R., Clarke R., Raisanen L. (2011). Free healthy breakfasts in primary schools: a cluster randomised controlled trial of a policy intervention in Wales, UK. Public Health Nutr.

[74] Shemilt I., Harvey I., Shepstone L., Swift L., Reading R., Mugford M. (2004). A national evaluation of school breakfast clubs: evidence from a cluster randomized controlled trial and an observational analysis. Child Care Health Dev.

[75] Feinstein L., Sabates R., Sorhaindo A., Rogers I., Herrick D., Northstone K. (2008). Dietary patterns related to attainment in school: the importance of early eating patterns. J. Epidemiol. Commun. Health..

[76] Golley R.K., Smithers L.G., Mittinty M.N., Emmett P., Northstone K., Lynch J.W. (2013). Diet quality of UK infants is associated with dietary, adiposity, cardiovascular, and cognitive outcomes measured at 7-8 years of age. J. Nutr..

[77] Mou Y., Blok E., Barroso M., Jansen P.W., White T., Voortman T. (2023). Dietary patterns, brain morphology and cognitive performance in children: results from a prospective population-based study. Eur. J. Epidemiol..

[78] Northstone K., Joinson C., Emmett P., Ness A., Paus T. (2012). Are dietary patterns in childhood associated with IQ at 8 years of age? A population-based cohort study. J. Epidemiol. Community Health..

[79] Nyaradi A., Li J., Hickling S., Whitehouse A.J.O., Foster J.K., Oddy W.H. (2013). Diet in the early years of life influences cognitive outcomes at 10 years: a prospective cohort study. Acta Paediatr.

[80] Nyaradi A., Oddy W.H., Hickling S., Li J., Foster J.K. (2015). The relationship between nutrition in infancy and cognitive performance during adolescence. Front. Nutr..

[81] Nyaradi A., Li J., Foster J.K., Hickling S., Jacques A., O'Sullivan T.A. (2016). Good-quality diet in the early years may have a positive effect on academic achievement. Acta Paediatr.

[82] Smithers L.G., Golley R.K., Mittinty M.N., Brazionis L., Northstone K., Emmett P. (2012). Dietary patterns at 6, 15 and 24 months of age are associated with IQ at 8 years of age. Eur. J. Epidemiol..

[83] Smithers L.G., Golley R.K., Mittinty M.N., Brazionis L., Northstone K., Emmett P. (2013). Do dietary trajectories between infancy and toddlerhood influence IQ in childhood and adolescence? Results from a prospective birth cohort study. PLoS One.

[84] Zhu Z., Cheng Y., Qi Q., Lu Y., Ma S., Li S. (2020). Association of infant and young child feeding practices with cognitive development at 10-12 years: a birth cohort in rural Western China. Br. J. Nutr..

[85] Algarín C., Nelson C.A., Peirano P., Westerlund A., Reyes S., Lozoff B. (2013). Iron-deficiency anemia in infancy and poorer cognitive inhibitory control at age 10 years. Dev. Med. Child Neurol..

[86] Lozoff B., Jimenez E., Hagen J., Mollen E., Wolf A.W. (2000). Poorer behavioral and developmental outcome more than 10 years after treatment for iron deficiency in infancy. Pediatrics.

[87] Lukowski A.F., Koss M., Burden M.J., Jonides J., Nelson C.A., Kaciroti N. (2010). Iron deficiency in infancy and neurocognitive functioning at 19 years: evidence of long-term deficits in executive function and recognition memory. Nutr. Neurosci..

[88] Åberg M.A.L., Åberg N., Brisman J., Sundberg R., Winkvist A., Torén K. (2009). Fish intake of Swedish male adolescents is a predictor of cognitive performance. Acta Paediatr.

[89] Kim J.L., Winkvist A., Åberg M.A., Åberg N., Sundberg R., Torén K. (2010). Fish consumption and school grades in Swedish adolescents: a study of the large general population. Acta Paediatr.

[90] Dubuc M.-M., Aubertin-Leheudre M., Karelis A.D. (2019). Lifestyle habits predict academic performance in high school students: the adolescent student academic performance longitudinal study (ASAP). Int. J. Environ. Res. Public Health..

[91] Faught E.L., Ekwaru J.P., Gleddie D., Storey K.E., Asbridge M., Veugelers P.J. (2017). The combined impact of diet, physical activity, sleep and screen time on academic achievement: a prospective study of elementary school students in Nova Scotia, Canada. Int. J. Behav. Nutr. Phys. Act..

[92] Faught E.L., Qian W., Carson V.L., Storey K.E., Faulkner G., Veugelers P.J. (2019). The longitudinal impact of diet, physical activity, sleep, and screen time on Canadian adolescents' academic achievement: an analysis from the COMPASS study. Prev. Med..

[93] Nigg C.R., Amato K. (2015). The influence of health behaviors during childhood on adolescent health behaviors, health indicators, and academic outcomes among participants from Hawaii. Int. J. Behav. Med..

[94] Nyaradi A., Foster J.K., Hickling S., Li J., Ambrosini G.L., Jacques A. (2014). Prospective associations between dietary patterns and cognitive performance during adolescence. J. Child Psychol. Psychiatry.

[95] Purtell K.M., Gershoff E.T. (2015). Fast food consumption and academic growth in late childhood. Clin. Pediatr. (Phila)..

[96] Hayek J., de Vries H., Tueni M., Lahoud N., Winkens B., Schneider F. (2021). Increased adherence to the Mediterranean diet and higher efficacy beliefs are associated with better academic achievement: a longitudinal study of high school adolescents in Lebanon. Int. J. Environ. Res. Public Health..

[97] Murphy J.M., Pagano M.E., Nachmani J., Sperling P., Kane S., Kleinman R.E. (1998). The relationship of school breakfast to psychosocial and academic functioning: cross-sectional and longitudinal observations in an inner-city school sample. Arch. Pediatr. Adolesc. Med..

[98] Powell C., Grantham-McGregor S., Elston M. (1983). An evaluation of giving the Jamaican government school meal to a class of children. Hum Nutr Clin Nutr..

[99] Samson K.L., Fischer J.A., Roche M.L. (2022). Iron status, anemia, and iron interventions and their associations with cognitive and academic performance in adolescents: a systematic review. Nutrients.

[100] Ahmed F., Khan M., Banu C., Qazi M., Akhtaruzzaman M. (2008). The coexistence of other micronutrient deficiencies in anaemic adolescent schoolgirls in rural Bangladesh. Eur. J. Clin. Nutr..

[101] Houghton L.A., Trilok-Kumar G., McIntosh D., Haszard J.J., Harper M.J., Reid M. (2019). Multiple micronutrient status and predictors of anemia in young children aged 12-23 months living in New Delhi, India. PLoS One.

[102] Karakaş N.M. (2021). The prevalence of low serum levels of Vitamin D, Vitamin B12, folate and ferritin in adolescents: single center experience. Sci. Prog..

[103] Smith E.M., Tangpricha V. (2015). Vitamin D and anemia: insights into an emerging association, Curr. Opin. Endocrinol. Diabetes Obes..

[104] Lane D.J., Richardson D.R. (2014). The active role of vitamin C in mammalian iron metabolism: much more than just enhanced iron absorption. Free Radic. Biol. Med..

[105] Bernal J., Feingold K.R., Adler R.A., Ahmed S.F. (2000). Endotext [Internet].

[106] Eastman C.J., Zimmermann M.B. (2015).

[107] Zimmermann M.B., Andersson M. (2021). Global endocrinology: global perspectives in endocrinology: coverage of iodized salt programs and iodine status in 2020. Eur. J. Endocrinol..

[108] Van den Briel T., West C.E., Bleichrodt N., van de Vijver F.J., Ategbo E.A., Hautvast J.G. (2000). Improved iodine status is associated with improved mental performance of schoolchildren in Benin. Am. J. Clin. Nutr..

[109] Woodside J.V., Mullan K.R. (2021). Iodine status in UK–an accidental public health triumph gone sour. Clin. Endocrinol..

[110] Li M., Eastman C.J. (2012). The changing epidemiology of iodine deficiency. Nat. Rev. Endocrinol..

[111] Van Der Wurff I.S.M., Meyer B.J., De Groot R.H.M. (2020). Effect of omega-3 long chain polyunsaturated fatty acids (N-3 LCPUFA) supplementation on cognition in children and adolescents: a systematic literature review with a focus on n-3 LCPUFA blood values and dose of DHA and EPA. Nutrients.

[112] Eilander A., Gera T., Sachdev H.S., Transler C., van der Knaap H.C., Kok F.J. (2010). Multiple micronutrient supplementation for improving cognitive performance in children: systematic review of randomized controlled trials. Am. J. Clin. Nutr..

[113] Bailey K.R.F., Pettersen J.A. (2024). Vitamin D is associated with visual memory in young northern adolescents. Nutr. Neurosci.

[114] Ford E.S., Zhao G., Tsai J., Li C. (2011). Associations between concentrations of vitamin D and concentrations of insulin, glucose, and HbA1c among adolescents in the United States. Diabetes Care.

[115] Kelishadi R., Ardalan G., Motlagh M.E., Shariatinejad K., Heshmat R., Poursafa P. (2014). National report on the association of serum vitamin D with cardiometabolic risk factors in the pediatric population of the Middle East and North Africa (MENA): the CASPIAN-III Study. Nutrition.

[116] Adolphus K., Lawton C.L., Champ C.L., Dye L. (2016). The effects of breakfast and breakfast composition on cognition in children and adolescents: a systematic review. Adv. Nutr..

[117] Adolphus K., Lawton C.L., Dye L. (2013). The effects of breakfast on behavior and academic performance in children and adolescents. Front. Hum. Neurosci..

[118] Edefonti V., Rosato V., Parpinel M., Nebbia G., Fiorica L., Fossali E. (2014). The effect of breakfast composition and energy contribution on cognitive and academic performance: a systematic review. Am. J. Clin. Nutr..

[119] Hoyland A., Dye L., Lawton C.L. (2009). A systematic review of the effect of breakfast on the cognitive performance of children and adolescents. Nutr. Res. Rev..

[120] Gaylor C., Benton D., Brennan A., Young H. (2022). The impact of glycaemic load on cognitive performance: a meta-analysis and guiding principles for future research. Neurosci. Biobehav. Rev..

[121] Bánáti D., Hellman-Regen J., Mack I., Young H.A., Benton D., Eggersdorfer M. (2024). Defining a vitamin A5/X specific deficiency–vitamin A5/X as a critical dietary factor for mental health. Int. J. Vitam. Nutr. Res..

[122] Young H.A., Geurts L., Scarmeas N., Benton D., Brennan L., Farrimond J. (2023). Multi-nutrient interventions and cognitive ageing: are we barking up the right tree?. Nutr. Res. Rev..

[123] Chugani H.T. (1998). A critical period of brain development: studies of cerebral glucose utilization with PET. Prev. Med..

[124] Prado E.L., Dewey K.G. (2014). Nutrition and brain development in early life. Nutr. Rev..

[125] Knickmeyer R.C., Gouttard S., Kang C., Evans D., Wilber K., Smith J.K. (2008). A structural MRI study of human brain development from birth to 2 years. J. Neurosci..

[126] Benton D. (2001). Micro-nutrient supplementation and the intelligence of children. Neurosci. Biobehav. Rev..

[127] Benton D. (2010). The influence of dietary status on the cognitive performance of children. Mol. Nutr. Food Res..

[128] Goodman A., Gregg P., Washbrook E. (2011). Children’s educational attainment and the aspirations, attitudes and behaviours of parents and children through childhood in the UK, Longitud. Life Course Stud.

[129] Stråvik M., Jonsson K., Hartvigsson O., Sandin A., Wold A.E., Sandberg A.-S. (2019). Food and nutrient intake during pregnancy in relation to maternal characteristics: results from the NICE Birth Cohort in Northern Sweden. Nutrients.

[130] Banerjee P.A. (2016). A systematic review of factors linked to poor academic performance of disadvantaged students in science and maths in schools. Cogent Educ.

[131] English L.K., Raghavan R., Obbagy J.E., Callahan E.H., Fultz A.K., Nevins J.E. (2024). Dietary patterns and health: insights from NESR systematic reviews to inform the Dietary Guidelines for Americans. J. Nutr. Educ. Behav..

[132] Benton D., Young H.A. (2024). Early exposure to sugar sweetened beverages or fruit juice differentially influences adult adiposity. Eur. J. Clin. Nutr..

[133] Der G., Batty G.D., Deary I.J. (2006). Effect of breast feeding on intelligence in children: prospective study, sibling pairs analysis, and meta-analysis. BMJ.

[134] Walfisch A., Sermer C., Cressman A., Koren G. (2013). Breast milk and cognitive development—the role of confounders: a systematic review. BMJ Open.

[135] Barros F.C., Victora C.G., Scherpbier R., Gwatkin D. (2010). Socioeconomic inequities in the health and nutrition of children in low/middle income countries. Rev. Saúde Pública..

[136] Glewwe P., Miguel E.A. (2007). The impact of child health and nutrition on education in less developed countries. Handb. Dev. Econ..

[137] Taylor R.M.J., Moore J.A., Griffiths A.R., Cousins A.L., Young H.A. (2025). Unveiling dietary complexity: a scoping review and reporting guidance for network analysis in dietary pattern research. Nutrients.

[138] Bugbee B.A., Beck K.H., Fryer C.S., Arria A.M. (2019). Substance use, academic performance, and academic engagement among high school seniors. J. Sch. Health.

[139] Cox R.G., Zhang L., Johnson W.D., Bender D.R. (2007). Academic performance and substance use: findings from a state survey of public high school students. J. Sch. Health.

[140] Henry K.L. (2010). Academic achievement and adolescent drug use: an examination of reciprocal effects and correlated growth trajectories. J. Sch. Health.

[141] Rosen J.A., Porter S.R., Rogers J. (2017). Understanding student self-reports of academic performance and course-taking behavior. AERA Open.

[142] Romijn A.R., Latulippe M.E., Snetselaar L., Willatts P., Melanson L., Gershon R. (2023). Perspective: advancing dietary guidance for cognitive health-focus on solutions to harmonization 3 of test selection, implementation, and evaluation. Adv. Nutr..

