# Supplementary material for: Diet and the Developing Brain: A Systematic Review of Nutritional Influences on Adolescent Cognitive and Academic Outcomes
Source: Adv Nutr. 2026 May 7;17(8):100648. doi: 10.1016/j.advnut.2026.100648 (PMC13430195; doi:10.1016/j.advnut.2026.100648)
Supplement: multimedia component 1 [file mmc1.docx]

**Diet and the Developing Brain: A Systematic Review of Nutritional Influences on Adolescent Cognitive and Academic Outcomes**

**Supporting Information**

Hayley A Young¹, Chantelle M Gaylor¹, Anthony Brennan¹, Abigail McIntosh¹, and Amy R Griffiths¹

¹ Swansea University, Wales, SA2 8PP, UK

**Supplementary Document 1**

- Table S1. Prisma Abstract Checklist
- Table S2. Prisma Checklist
- Table S3: Preliminary eligibility criteria
- Table S4: Cochrane Risk of Bias 2 assessment of cluster-randomised controlled trials.
- Table S5: Cochrane Risk of Bias 2 assessment of randomised controlled trials.
- Table S6: Joanna Briggs Institute Critical Appraisal Tool for non-randomised controlled trials.
- Table S7. Joanna Briggs Institute Critical Appraisal Tool for prospective studies (beginning in infancy).
- Table S8. Joanna Briggs Institute Critical Appraisal Tool for prospective studies (beginning in early adolescence).

**Table S1. Prisma Abstract Checklist.**

| - **Section and Topic** | **Item #** | **Checklist item** | **Reported (Yes/No)** |
| --- | --- | --- | --- |
| **TITLE** | | |  |
| Title | 1 | Identify the report as a systematic review. | yes |
| **BACKGROUND** | | |  |
| Objectives | 2 | Provide an explicit statement of the main objective(s) or question(s) the review addresses. | yes |
| **METHODS** | | |  |
| Eligibility criteria | 3 | Specify the inclusion and exclusion criteria for the review. | yes |
| Information sources | 4 | Specify the information sources (e.g. databases, registers) used to identify studies and the date when each was last searched. | yes |
| Risk of bias | 5 | Specify the methods used to assess risk of bias in the included studies. | yes |
| Synthesis of results | 6 | Specify the methods used to present and synthesise results. | yes |
| **RESULTS** | | |  |
| Included studies | 7 | Give the total number of included studies and participants and summarise relevant characteristics of studies. | yes |
| Synthesis of results | 8 | Present results for main outcomes, preferably indicating the number of included studies and participants for each. If meta-analysis was done, report the summary estimate and confidence/credible interval. If comparing groups, indicate the direction of the effect (i.e. which group is favoured). | yes |
| **DISCUSSION** | | |  |
| Limitations of evidence | 9 | Provide a brief summary of the limitations of the evidence included in the review (e.g. study risk of bias, inconsistency and imprecision). | yes |
| Interpretation | 10 | Provide a general interpretation of the results and important implications. | yes |
| **OTHER** | | |  |
| Funding | 11 | Specify the primary source of funding for the review. | yes |
| Registration | 12 | Provide the register name and registration number. | yes |

*From:*  Page MJ, McKenzie JE, Bossuyt PM, Boutron I, Hoffmann TC, Mulrow CD, et al. The PRISMA 2020 statement: an updated guideline for reporting systematic reviews. BMJ 2021;372:n71. doi: 10.1136/bmj.n71.

**Table S2. Prisma Checklist**

| **Section and Topic** | **Item #** | **Checklist item** | **Location where item is reported** |
| --- | --- | --- | --- |
| **TITLE** | | |  |
| Title | 1 | Identify the report as a systematic review. | Page 1 |
| **ABSTRACT** | | |  |
| Abstract | 2 | See the PRISMA 2020 for Abstracts checklist. | Page 1 |
| **INTRODUCTION** | | |  |
| Rationale | 3 | Describe the rationale for the review in the context of existing knowledge. | Section 1 line numbers (41-55) |
| Objectives | 4 | Provide an explicit statement of the objective(s) or question(s) the review addresses. | Section 1 line numbers (56-67) |
| **METHODS** | | |  |
| Eligibility criteria | 5 | Specify the inclusion and exclusion criteria for the review and how studies were grouped for the syntheses. | Sections 2.2 line numbers (98-130) and 2.4 line numbers (139-143), and Table S3. |
| Information sources | 6 | Specify all databases, registers, websites, organisations, reference lists and other sources searched or consulted to identify studies. Specify the date when each source was last searched or consulted. | Section 2.1 line numbers (77-80) |
| Search strategy | 7 | Present the full search strategies for all databases, registers and websites, including any filters and limits used. | Section 2.1 line numbers (77-96) |
| Selection process | 8 | Specify the methods used to decide whether a study met the inclusion criteria of the review, including how many reviewers screened each record and each report retrieved, whether they worked independently, and if applicable, details of automation tools used in the process. | Section 2.1 line numbers (89-96) |
| Data collection process | 9 | Specify the methods used to collect data from reports, including how many reviewers collected data from each report, whether they worked independently, any processes for obtaining or confirming data from study investigators, and if applicable, details of automation tools used in the process. | Section 2.3 line numbers (132-137) |
| Data items | 10a | List and define all outcomes for which data were sought. Specify whether all results that were compatible with each outcome domain in each study were sought (e.g. for all measures, time points, analyses), and if not, the methods used to decide which results to collect. | Section 2.4 line numbers (139 - 143) |
|  | 10b | List and define all other variables for which data were sought (e.g. participant and intervention characteristics, funding sources). Describe any assumptions made about any missing or unclear information. | Section 2.3 line numbers (132-137) |
| Study risk of bias assessment | 11 | Specify the methods used to assess risk of bias in the included studies, including details of the tool(s) used, how many reviewers assessed each study and whether they worked independently, and if applicable, details of automation tools used in the process. | Section 2.6 line numbers (156-164) |
| Effect measures | 12 | Specify for each outcome the effect measure(s) (e.g. risk ratio, mean difference) used in the synthesis or presentation of results. | N/A |
| Synthesis methods | 13a | Describe the processes used to decide which studies were eligible for each synthesis (e.g. tabulating the study intervention characteristics and comparing against the planned groups for each synthesis (item #5)). | Section 2.2 line numbers (98-130) |
|  | 13b | Describe any methods required to prepare the data for presentation or synthesis, such as handling of missing summary statistics, or data conversions. | N/A |
|  | 13c | Describe any methods used to tabulate or visually display results of individual studies and syntheses. | Tables 1–3  Pages 26-66 |
|  | 13d | Describe any methods used to synthesize results and provide a rationale for the choice(s). If meta-analysis was performed, describe the model(s), method(s) to identify the presence and extent of statistical heterogeneity, and software package(s) used. | Section 2.5 line numbers (145-155) |
|  | 13e | Describe any methods used to explore possible causes of heterogeneity among study results (e.g. subgroup analysis, meta-regression). | N/A |
|  | 13f | Describe any sensitivity analyses conducted to assess robustness of the synthesized results. | N/A |
| Reporting bias assessment | 14 | Describe any methods used to assess risk of bias due to missing results in a synthesis (arising from reporting biases). | N/A |
| Certainty assessment | 15 | Describe any methods used to assess certainty (or confidence) in the body of evidence for an outcome. | N/A |
| **RESULTS** | | |  |
| Study selection | 16a | Describe the results of the search and selection process, from the number of records identified in the search to the number of studies included in the review, ideally using a flow diagram. | Section 3.1 line numbers (167-174) See also Figure 1 |
|  | 16b | Cite studies that might appear to meet the inclusion criteria, but which were excluded, and explain why they were excluded. | Section 3.1 line numbers (168) See also Figure 1 |
| Study characteristics | 17 | Cite each included study and present its characteristics. | Sections 3.2.1, 3.3.1, and 3.4.1. |
| Risk of bias in studies | 18 | Present assessments of risk of bias for each included study. | Sections 3.2.2, 3.3.2, and 3.4.2. |
| Results of individual studies | 19 | For all outcomes, present, for each study: (a) summary statistics for each group (where appropriate) and (b) an effect estimate and its precision (e.g. confidence/credible interval), ideally using structured tables or plots. | N/A |
| Results of syntheses | 20a | For each synthesis, briefly summarise the characteristics and risk of bias among contributing studies. | Sections 3.2.2 line numbers (191-201); 3.3.2 line numbers(378-382); 3.4.2 line numbers (441 – 447) |
|  | 20b | Present results of all statistical syntheses conducted. If meta-analysis was done, present for each the summary estimate and its precision (e.g. confidence/credible interval) and measures of statistical heterogeneity. If comparing groups, describe the direction of the effect. | N/A |
|  | 20c | Present results of all investigations of possible causes of heterogeneity among study results. | N/A |
|  | 20d | Present results of all sensitivity analyses conducted to assess the robustness of the synthesized results. | N/A |
| Reporting biases | 21 | Present assessments of risk of bias due to missing results (arising from reporting biases) for each synthesis assessed. | Sections 3.3.2 line numbers (380-382), and 3.4.2. (444 – 447) |
| Certainty of evidence | 22 | Present assessments of certainty (or confidence) in the body of evidence for each outcome assessed. | N/A |
| **DISCUSSION** | | |  |
| Discussion | 23a | Provide a general interpretation of the results in the context of other evidence. | Sections 4.1 – 4.3 line numbers (494-546) |
|  | 23b | Discuss any limitations of the evidence included in the review. | Sections 4.1 line numbers (514-518); 4.4 line numbers (548 -553) |
|  | 23c | Discuss any limitations of the review processes used. | N/A |
|  | 23d | Discuss implications of the results for practice, policy, and future research. | Sections 4.5 line numbers (555 – 590); Section 4.6 line numbers (597 – 601). |
| **OTHER INFORMATION** | | |  |
| Registration and protocol | 24a | Provide registration information for the review, including register name and registration number, or state that the review was not registered. | Section 2 line numbers (70-73) |
|  | 24b | Indicate where the review protocol can be accessed, or state that a protocol was not prepared. | Section 2 line numbers (70-73) |
|  | 24c | Describe and explain any amendments to information provided at registration or in the protocol. | Section 2 line numbers (84-88) |
| Support | 25 | Describe sources of financial or non-financial support for the review, and the role of the funders or sponsors in the review. | Page 1 |
| Competing interests | 26 | Declare any competing interests of review authors. | Page 1 |
| Availability of data, code and other materials | 27 | Report which of the following are publicly available and where they can be found: template data collection forms; data extracted from included studies; data used for all analyses; analytic code; any other materials used in the review. | Page 1 |

*From:*  Page MJ, McKenzie JE, Bossuyt PM, Boutron I, Hoffmann TC, Mulrow CD, et al. The PRISMA 2020 statement: an updated guideline for reporting systematic reviews. BMJ 2021;372:n71. doi: 10.1136/bmj.n71

**Table S3: Preliminary eligibility criteria**

| **Inclusion criteria** |
| --- |
| 1. Studies involving males and/or female adolescents (10 – 19 years_1_) from the general population_2_ or with pre-existing nutritional deficiencies. 2. Acute_3_ or chronic_4_ RCTs or non-RCTs or cross-sectional studies examining the effects of diet on cognitive and/or academic performance during adolescence. 3. Prospective studies examining the association between diet during infancy (<3 years) and cognitive or academic performance during adolescence. 4. Prospective studies examining the association between diet during early adolescence and cognitive or academic performance during late adolescence. 5. Full-text articles. |
| **Exclusion criteria** |
| 1. Animal studies. 2. Protocols, reviews, or meta-analyses. 3. Studies involving undergraduate samples or children <10 years of age. 4. Studies involving adolescents with a pre-existing mental (e.g., ADHD) or physical disorder (e.g., diabetes or epilepsy). 5. Studies examining the effect of diet on mood, mental health, behaviour, or clinical outcomes (e.g., risk of ADHD or autism). 6. Studies examining eating behaviour. 7. Studies examining the effects of nutritive or non-nutritive sweeteners, alcohol, or caffeine on cognitive and/or academic performance. 8. Studies that examined the effect of diet on brain function/structure or nutritional status but did not relate these measures to cognitive or academic performance. 9. Prospective studies examining the impact of diet during adolescence on cognition during adulthood. 10. Prospective studies that exclusively examined the effect of breastfeeding or malnutrition during infancy on cognitive and/or academic performance during adolescence. 11. Retrospective studies. |

1 = World Health Organisation’s definition of adolescence, 2 = The NASEMs definition of general population includes individuals with or at risk for chronic disease including overweight or obesity, unless there was a reason for exception that applied specifically to the nutrient(s) being studied, 3 = single consumption, and 4 = intervention period lasting >1 month.

**Table S4. Cochrane Risk of Bias 2 assessment of cluster-randomised controlled trials.**

| **Author (year)** | **Randomisation process** | **Timing of identification or recruitment** | **Deviations from intended interventions** | **Missing outcome data** | **Measurement of the outcome** | **Selection of the reported result** | **Overall** |
| --- | --- | --- | --- | --- | --- | --- | --- |
| Buzina-Suboticanec et al. (1998) |  |  |  |  |  |  |  |
| Handeland et al. (2017) |  |  |  |  |  |  |  |
| Handeland et al. (2018) |  |  |  |  |  |  |  |
| Kalaichelvi (2016) |  |  |  |  |  |  |  |
| Murphy et al. (2011) |  |  |  |  |  |  |  |
| Rezaeian et al. (2014) |  |  |  |  |  |  |  |
| Schoenthaler et al. (1991) |  |  |  |  |  |  |  |
| Sen & Kanani (2009) |  |  |  |  |  |  |  |
| Shemilt et al. (2004) |  |  |  |  |  |  |  |
| Sorensen et al. (2015a) |  |  |  |  |  |  |  |
| Sorensen et al. (2015b) |  |  |  |  |  |  |  |
| Sorensen et al. (2016) |  |  |  |  |  |  |  |
| Wang et al. (2017) |  |  |  |  |  |  |  |

*Note.* ‘+’ = low risk of bias, ‘-‘= high risk of bias, and ‘!’ = some concerns of bias.

**Table S5. Cochrane Risk of Bias 2 assessment of randomised controlled trials.**

| **Author (year)** | **Randomisation process** | **Deviations from intended interventions** | **Missing outcome data** | **Measurement of the outcome** | **Selection of the reported result** | **Overall** |
| --- | --- | --- | --- | --- | --- | --- |
| Bruner et al. (1996) |  |  |  |  |  |  |
| Chellappa and Karunanidhi (2012) |  |  |  |  |  |  |
| Chung et al. (2012) |  |  |  |  |  |  |
| Gordon et al. (2009) |  |  |  |  |  |  |
| Grung et al. (2017) |  |  |  |  |  |  |
| Haskell et al. (2008) |  |  |  |  |  |  |
| Huda et al. (2001) |  |  |  |  |  |  |
| Kashyap and Gopaldas (1987) |  |  |  |  |  |  |
| Kennedy et al. (2009) |  |  |  |  |  |  |
| Kirby et al. (2010) |  |  |  |  |  |  |
| Lambert et al. (2002) |  |  |  |  |  |  |
| Lynn and Harland (1998) |  |  |  |  |  |  |
| McNamara et al. (2010) |  |  |  |  |  |  |
| Nidich et al. (1993) |  |  |  |  |  |  |
| O’Connor et al. (2022) |  |  |  |  |  |  |
| Perlman et al. (2010) |  |  |  |  |  |  |
| Petrova et al. (2019) |  |  |  |  |  |  |
| Pinar-Marti et al. (2023) |  |  |  |  |  |  |
| Pollitt, Soemantri et al. (1985); Pollitt (1997) |  |  |  |  |  |  |
| Pollitt et al. (1989) |  |  |  |  |  |  |
| Portillo-Reyes et al. (2014) |  |  |  |  |  |  |
| Scott et al. (2018) |  |  |  |  |  |  |
| Snowden (1997) |  |  |  |  |  |  |
| Soemantri et al. (1985) |  |  |  |  |  |  |
| Soemantri (1989) |  |  |  |  |  |  |
| Tefagh et al. (2022) |  |  |  |  |  |  |
| Teisen et al. (2020) |  |  |  |  |  |  |
| Van der Wurff et al. (2019) |  |  |  |  |  |  |
| Van der Wurff et al. (2023) |  |  |  |  |  |  |
| Zimmerman et al. (2006) |  |  |  |  |  |  |

*Note.* ‘+’ = low risk of bias, ‘-‘= high risk of bias, and ‘!’ = some concerns of bias.

**Table S6. Joanna Briggs Institute Critical Appraisal Tool for non-randomised controlled trials.**

| **Author (year)** | **Is it clear in the study what is the ‘cause’ and what is the ‘effect’?** | **Were the participants included in any comparisons similar?** | **Were the participants included in any comparisons receiving similar treatment/**  **care, other than the exposure or intervention of interest?** | **Was there a control group?** | **Were there multiple measurements of the outcome both pre and post the intervention/**  **exposure?** | **Was follow up complete and if not, were differences between groups in terms of their follow up adequately described and analysed?** | **Were the outcomes of participants included in any comparisons measured in the same way?** | **Were outcomes measured in a reliable way?** | **Was appropriate statistical analysis used?** | **Overall risk of bias** |
| --- | --- | --- | --- | --- | --- | --- | --- | --- | --- | --- |
| Cueto and Chinen (2008) | Yes | No | Yes | Yes | No | Unclear | Yes | Yes (cognitive tests). No (academic tests) | Unclear | **Moderate** |
| Devaki et al. (2009) | Yes | Yes | Yes | Yes | Yes | Yes | Yes | Yes | Unclear | **Low** |
| Isa et al. (2000) | Yes | No | Unclear | Yes | Yes | No | Yes | Yes | Unclear | **Moderate** |
| Karkada et al. (2019) | Yes | Unclear | Yes | Yes | Yes | Yes | Yes | Unclear | Unclear | **Moderate** |
| Southon et al. (1994) | Yes | Yes | Yes | Yes | Yes | No | Yes | Yes | Unclear | **Low** |

**Table S7. Joanna Briggs Institute Critical Appraisal Tool for prospective studies (beginning in infancy).**

| **Author (year)** | Were the two groups similar and recruited from the same population? | Were the exposures measured similarly to assign people to both exposed and unexposed groups? | Was the exposure measured in a valid and reliable way? | Were confounding factors identified? | Were strategies to deal with confounding factors stated? | Were the groups/ participants free of the outcome at the start of the study (or at the moment of exposure)? | Were the outcomes measured in a valid and reliable way? | Was the follow up time reported and sufficient to be long enough for outcomes to occur? | Was (A) follow up complete, and if not, were (B) the reasons to loss to follow up described and explored? | Were strategies to address incomplete follow up utilized? | Was an appropriate statistical analysis used? | Overall risk of bias |
| --- | --- | --- | --- | --- | --- | --- | --- | --- | --- | --- | --- | --- |
| Algarin et al. (2013) | Yes | Yes | Yes | Yes_2_ | Yes | N/A | Yes | Yes | (A) No, (B) No | No | **Yes** | **Low** |
| Feinstein et al. (2008) | Yes | Yes | Yes | No_1_ | Yes | N/A | Yes | Yes | (A) No, (B) Yes | No | **Yes** | **Low** |
| Golley et al. (2013) | Yes | Yes | Yes | No_1_ | Yes | N/A | Yes | Yes | (A) No, (B) Yes | No | **Yes** | **Low** |
| Lozoff et al. (2000) | Yes | Yes | Yes | Yes_2_ | Yes | N/A | Yes | Yes | (A) No, (B) Yes | No | **Yes** | **Low** |
| Lukowski et al. (2010) | Yes | Yes | Yes | Yes_2_ | Yes | N/A | Yes | Yes | (A) No, (B) Yes | No | **Yes** | **Low** |
| Northstone et al. (2012) | Yes | Yes | Yes | No_1_ | Yes | N/A | Yes | Yes | (A) No, (B) Yes | No | **Yes** | **Low** |
| Nyaradi et al. (2013) | Yes | Yes | No | No_1_ | Yes | N/A | Yes | Yes | (A) No, (B) Yes | No | **Yes** | **Moderate** |
| Nyaradi et al. (2015) | Yes | Yes | No | No_1_ | Yes | N/A | Yes | Yes | (A) No, (B) Yes | No | **Yes** | **Moderate** |
| Nyaradi et al. (2016) | Yes | Yes | No | No_1_ | Yes | N/A | Yes | Yes | (A) No, (B) Yes | No | **Yes** | **Moderate** |
| Mou et al. (2023) | Yes | Yes | Yes | No_1_ | Yes | N/A | Yes | Yes | (A) No, (B) Yes | Yes | **Yes** | **Low** |
| Smithers et al. (2012) | Yes | Yes | Yes | No_1_ | Yes | N/A | Yes | Yes | (A) No, (B) Yes | Yes | **Yes** | **Low** |
| Smithers et al. (2013) | Yes | Yes | Yes | No_1_ | Yes | N/A | Yes | Yes | (A) No, (B) Yes | Yes | **Yes** | **Low** |
| Zhu et al. (2020) | Yes | Yes | Yes | No_1_ | Yes | N/A | Yes | Yes | (A) No, (B) Yes | Yes | **Yes** | **Low** |

Low = ‘yes’ to <50% of questions, moderate = 51-70% of questions, and high = >71% of questions.

1 = several key confounding factors were identified but other key confounders were missing (e.g. current/previous diet or maternal IQ), and 2 = infants took part in a randomised controlled trial.

**Table S8. Joanna Briggs Institute Critical Appraisal Tool for prospective studies (beginning in early adolescence).**

| **Author (year)** | Were the two groups similar and recruited from the same population? | Were the exposures measured similarly to assign people to both exposed and unexposed groups? | Was the exposure measured in a valid and reliable way? | Were confounding factors identified? | Were strategies to deal with confounding factors stated? | Were the groups/ participants free of the outcome at the start of the study (or at the moment of exposure)? | Were the outcomes measured in a valid and reliable way? | Was the follow up time reported and sufficiently long enough for outcomes to occur? | Was (A) follow up complete, and if not, were (B) the reasons to loss to follow up described and explored? | Were strategies to address incomplete follow up utilized? | Was an appropriate statistical analysis used? | Overall risk of bias |
| --- | --- | --- | --- | --- | --- | --- | --- | --- | --- | --- | --- | --- |
| Aberg et al. (2009) | Yes | Yes | No | No_3_ | Yes | N/A | Yes | Yes | (A) No, (B) Yes | No | Yes | **Moderate** |
| Kim et al. (2010) | Yes | Yes | No | No_3_ | Yes | N/A | Yes | No_6_ | (A) No, (B) No | No | Yes | **High** |
| Dubuc et al. (2020) | Yes | Yes | No | No_3_ | Yes | N/A | Yes | Yes | (A) No, (B) Yes | No | Yes | **Moderate** |
| Faught et al. (2017) | Yes | Yes | No | No_3_ | Yes | N/A | Yes | No_6_ | (A) No, (B) Yes | Yes | Yes | **Moderate** |
| Faught et al. (2019) | Yes | Yes | No | No_3_ | Yes | N/A | No_5_ | No_6_ | (A) No, (B) Yes | No | Yes | **High** |
| Mou et al. (2023) | Yes | Yes | Yes | No_3_ | Yes | N/A | Yes | Yes | (A) No, (B) Yes | Yes | Yes | **Low** |
| Nigg and Amato (2015) | Yes | Yes | No | No_3_ | Yes | N/A | No_5_ | Yes | (A) No, (B) Yes | No | Yes | **Moderate** |
| Nyaradi et al. (2014) | Yes | Yes | Yes | No_3_ | Yes | N/A | Yes | Yes | (A) No, (B) Yes | No | Yes | **Low** |
| Purtell and Gershoff (2015) | Yes | Yes | No | No_3_ | Yes | N/A | Yes | Yes | (A) No, (B) Yes | No | Yes | **Moderate** |
| Hayek et al. (2021) | Yes | Yes | Yes | No_3_ | Yes | N/A | No_5_ | No_6_ | (A) No, (B) No | Yes | Yes | **Moderate** |
| Murphy et al. (1998) | Yes | Yes | No | No_4_ | No | N/A | Yes | Yes* | (A) No, (B) Yes | No | No_4_ | **High** |
| Powell (1983) | Unclear_1_ | Yes | Unclear_2_ | No_3_ | Yes | N/A | Yes | Yes* | Unclear | No | Yes | **High** |

Low = ‘yes’ to <50% of questions, moderate = 51-70% of questions, and high = >71% of questions.

1 = unclear if groups were similar at baseline, 2 = unclear if participants complied with the intervention, 3 = key confounding factors were identified, but the number of included covariates was small, measures of socioeconomic status were unsatisfactory (spending money), diet was only assessed at baseline, outcomes were only assessed at follow-up, diet was assessed using <3 questions, and/or other relevant aspects of diet were not measured, 4 = confounding factors were not identified, 5 = self-report measures of academic achievement were obtained, and 6 = 1-year follow-up period. * Participants were followed-up after 3 to 4 months, which was considered sufficient given that school breakfast programmes could potentially improve academic achievement via multiple short-term and long-term mechanisms.
