# Supplementary material for: Diet and the Developing Brain: A Systematic Review of Nutritional Influences on Adolescent Cognitive and Academic Outcomes
Source: Adv Nutr. 2026 May 7;17(8):100648. doi: 10.1016/j.advnut.2026.100648 (PMC13430195; doi:10.1016/j.advnut.2026.100648)
Supplement: multimedia component 2 [file mmc2.docx]

**Diet and the Developing Brain: A Systematic Review of Nutritional Influences on Adolescent Cognitive and Academic Outcomes**

**Supporting Information**

Hayley A Young¹, Chantelle M Gaylor¹, Anthony Brennan¹, Abigail McIntosh¹, and Amy R Griffiths¹

¹ Swansea University, Wales, SA2 8PP, UK

## Supplementary Tables S9-S11

**Table S9.** Randomized and non-randomized controlled trials.

| **Author (year)** | **Sample** | **Dietary intervention** | **Outcome measures** | | | **Results** |
| --- | --- | --- | --- | --- | --- | --- |
|  |  |  | **Cognition** | **School performance** | **Dietary intake and biochemical measures** |  |
| **LCPUFA interventions (n = 10)** | | | | | | |
| Handeland et al. (2017) | N = 426 (204 males, 222 females).  Mean age = 14.6 years (age range = 14-15 years).  8 schools.  Norway.  Mixed SES.  40% and 92% were iodine or vitamin D deficient at baseline, respectively. 95% had an omega-3 index ≤8% at baseline.  FINS-TEENS study. | 3-month, BS, cluster-RCT.  Clustered by class.    1) Active (80-100g fatty fish 3 times p/w, providing  152mg/100g EPA, 262mg/100g DHA, and 40mg/100g DPA per serving)  2) Control (80-100g meat 3 times p/w, providing  3.2mg/100g EPA, 5mg/100g DHA, and 6mg/100g DPA per serving)  3) Control (habitual lunchtime meal plus 7 fish oil capsules [corresponding to 90g fatty fish] 3 times p/w, providing 158mg EPA, 105mg DHA, and 13mg DPA per capsule). | Administered pre- and post-intervention:  D2 Test of Attention (attention and information processing speed). | Administered pre- and post-intervention:  Kartleggeren (Norwegian spelling and reading test. Data not reported due to ceiling effects). | N/A | The fish group showed a significantly larger improvement in information processing speed compared to both control groups. Attention also improved to a significantly larger extent in the fish group compared to the supplement group. Omission errors (inattention) decreased in the meat group compared to the fish group after adjusting for baseline performance, but not when additionally adjusting for dietary compliance. |
| Handeland et al. (2018)  FINS-TEENS study. | See Handeland et al. (2017) | See Handeland et al. (2017) | See Handeland et al. (2017) | N/A | Measured pre- and post-intervention:  Fatty acid composition, vitamin D, ferritin, and UIC. | DHA levels and omega-3 index increased to a significantly larger extent in the supplement group compared to the fish and meat group, and fish group compared to the meat group. No changes in vitamin D, ferritin, or UIC were reported.  A mediation analysis was not performed as biochemical measures were not associated with post-intervention cognitive scores, |
| Kennedy et al. (2009) | N = 86 (44 males, 42 females)  Mean age = 10.83 years (age range = 10-12 years).  Number of schools not reported.  UK. | 2-month, double-blind, BS, RCT.  1) Active low dose (algae oil and high oleic sunflower oil supplements, providing 400mg DHA and 8mg EPA p/d)  2) Active high dose (algae oil and high oleic sunflower oil supplements, providing 1000mg DHA and 20mg of EPA p/d)  3) Control (placebo supplements, providing 0mg DHA, 15mg ALA, and 250mg LA p/d). | Administered pre- and post-intervention (before and after a standard breakfast):  Internet Battery (Word List Recognition test [verbal memory], Picture Recognition test [visual memory], Arrow RT test [information processing speed], Arrow Flanker test [selective attention and inhibition], PAL [spatial memory], and Sentence Verification test [semantic memory]); Cognitive Drug Research battery (Picture Recognition test [visual memory], Word List Recall/Recognition test [verbal memory], Simple and Choice RT test [information processing speed], Digit Vigilance test [attention], Numeric Working Memory test [working memory], and Spatial Working Memory test [spatial working memory]). | N/A | N/A | Delayed verbal memory (Cognitive Drug Research battery) significantly improved in the low dose group versus control group when measured pre- and post-breakfast. Conversely, delayed verbal memory declined in the high dose group versus control group when measured pre-breakfast. |
| Kirby et al. (2010) | N = 348 (168 males, 180 females).  Age range = 8-9 years.  1 school.  UK.  Mixed SES. | 17-week, BS, double-blind, RCT.  1) Active (fish oil supplements, providing 200mg DHA, 28mg EPA, 400ug vitamin A, 30mg vitamin C, 2.5ug vitamin D, and 1.5mg vitamin E p/d)  2) Control (placebo). | Administered pre- and post-intervention:  Kaufman Brief Intelligence Test-2 (verbal, non-verbal, and overall IQ); Working Memory Test Battery for Children (working memory); Creature Counting (attention); Matching Familiar Figure Test (impulsivity). | Administered pre- and post-intervention:  Wechsler Individual Achievement Test-II (reading and spelling). | Measured pre- and post-intervention:  Cheek cell fatty acid composition. | EPA levels significantly increased from pre- to post-intervention in the active group only. DHA levels increased in both groups, but to a larger extent in the active group.  Intention-to-treat analysis did not reveal any significant changes. Per-protocol analysis showed that problem solving significantly improved from pre- to post-intervention in the active group only. |
| McNamara et al. (2010) | N = 33 males.  Mean age = 9.2 years (age range = 8-10 years).  USA. | 2-month, double-blind, BS, RCT.  1) Active low dose (algae oil supplements, providing 400mg DHA and 0mg EPA p/d)  2) Active high dose (algae oil supplements, providing 1200mg DHA and 0mg EPA p/d)  3) Control (placebo). | Administered pre- and post-intervention:  CPT-Identical Pairs (attention). | N/A | Measured pre- and post-intervention:  Brain functioning (fMRI scan).  DHA, AA, and LA. | DHA levels significantly increased by 47% in the low dose group and 70% in the high dose group after 2 months, whereas levels declined by 11% in the control group.  Although no changes in attention were reported, both the low and high dose groups exhibited significantly increased functional activation in the DLPFC during the attention task compared to the control group. Lower activation was also observed in the occipital cortex (low dose group) and cerebellar cortex (high dose group) than the control group. |
| Pinar-Martí et al. (2023) | N = 771 (237 males, 216 females).  Mean age = 13.9 years (age range = 11-16 years).  12 schools.  Spain.  Mixed SES. | 6-month, BS, RCT.  1) Active (habitual diet plus 30g walnuts p/d, providing 2.7g of ALA p/d)  2) Control (habitual diet). | Administered pre- and post-intervention:  Attention Network test (attention); n-back test (working memory); inductive reasoning subtest of the Primary Mental Abilities Test (non-verbal IQ - inductive reasoning). | N/A | Measured pre- and post-intervention:  Fatty acid composition. | Intention-to-treat analysis revealed no changes in cognition. ALA levels increased to a larger extent from pre- to post-intervention in the active vs control group.  Per-protocol analysis (restricted to those who consumed walnuts for >100 days) revealed a larger improvement in attention and non-verbal IQ in the active vs control group. |
| Portillo-Reyes et al. (2014) | N = 59 (25 male, 34 females).  Mean age = 9.22 years (age range = 8-12 years).  2 schools of low SES (purposively selected).  Mexico.  Participants were mild to moderately malnourished at baseline (according to anthropometric measures). | 3-month, double-blind, BS, RCT.  1) Active (fish oil supplements, providing 180mg DHA and 270mg EPA p/d)  2) Control (placebo). | Administered pre- and post-intervention:  WISC-IV (Symbol Search test [information processing speed], Letter-Number Sequencing test [working memory], Block Design test and Raven’s Matrices test [non-verbal IQ - perceptual reasoning], Semantic Fluency test [verbal fluency], and Letter-Cancellation test [information processing speed]); Embedded Figures and Visual Closure tests (perceptual integration); Stroop test (inhibition); TMT-A (information processing speed); TMT-B (mental flexibility/shifting); Rey Complex Figure test (visual memory); Word List Recall test (verbal memory). | Measured pre- and post-intervention:  Academic grades (language, mathematics, history, geography, science, and civic education). | N/A | Information processing speed (Symbol Search test only), perceptual integration, inhibition, and non-verbal IQ improved in the active group but not the control group from pre- to post-intervention.  >70% of the active group had a clinically significant improvement (Cohen’s d >0.8) in information processing speed, perceptual integration (Embedded Figures test only), non-verbal IQ, and working memory compared to <25% of the control group. |
| Teisen et al. (2020) | N = 199 (100 males, 99 females).  Age range = 8-9 years.  Denmark.  Mixed SES. | 4-month, BS, RCT.  1) Active (300g oily fish p/w, providing 0.8-1g p/d omega-3 fatty acids)  2) Control (300g poultry p/w). | Administered pre- and post-intervention:  Cambridge Neuropsychological Test Automated Battery (RT test and Rapid Visual Processing test [information processing speed], PAL test [spatial memory], and Spatial Working Memory test [spatial working memory]); D2 Test of Attention (attention and information processing speed); Stroop test (inhibition); Arrow Flanker test (sustained attention and inhibition); Adapted Arrow Flanker test (mental flexibility/shifting). | N/A | Measured pre- and post-intervention:  Fatty acid composition and vitamin D. | Omega-3 levels (EPA+DHA, DHA, and EPA) and vitamin D levels increased to a significantly larger extent in the active group than control group. Omega-6 levels increased in the control group only.  The active group made fewer information processing speed errors (Rapid Visual Processing test only) than the control group post-intervention, as well as faster mental flexibility/shifting. |
| van der Wurff et al. (2019)  Food2Learn | N = 256 (123 males, 133 females).  Mean age = 14.11 years (age range = 13-15 years).  17 schools.  Netherlands.  Participants had a low omega-3 index at baseline (<5%). Aimed to increase to 8-11% after 3 months. | 12-month, double-blind, BS, RCT.  Cohort 1:  1) Active (fish oil supplements, providing 140mg DHA and 260mg EPA p/d for the first 3 months. Dosage increased to 280mg DHA and 520mg EPA p/d for the remaining 9 months)  2) Control (placebo).    Cohort 2:  1) Active (fish oil supplements, providing 280mg DHA and 520mg EPA p/d)  2) Control (placebo). | Administered at baseline and after 6- and 12-months:  Letter Digit Substitution Test (information processing speed); D2 Test of Attention (attention and information processing speed); Digit Span Forwards and Backwards test (working memory); Stroop test (inhibition); Concept Shifting Test (mental flexibility/ shifting). | N/A | Measured at baseline and after 3-, 6- and 12-months:  Fatty acid composition. | Compared to the control group, the active group had significantly higher levels of EPA, DPA, and DHA, significantly lower levels of omega-6 (AA and ObA), and a significantly higher omega-3 index at 3-, 6-, and 12-months. However, participants omega-3 index remained within the suboptimal range post-intervention.  Performance significantly improved on all cognitive tasks throughout the intervention, however there were no significant differences between groups. The improvement in attention from 6 to 12 months was significantly larger in the control group than active group. However, as the effect was small, and the control group scored higher on the D2 Test of Attention at all time points, this was not considered notable.  No association between changes in omega-3 index and cognition. |
| van der Wurff et al. (2023)  Food2Learn | See van der Wurff et al. (2019) | See van der Wurff et al. (2019) | N/A | Measured pre- and post-intervention:  Academic grades (Dutch, English, and mathematics). | See van der Wurff et al. (2019) | No changes in grades from pre- to post-intervention. No association between omega-3 index and academic performance. |
| **Choline (n = 1)** | | | | | | |
| O'Connor et al. (2022) | N = 122 (64 males, 58 females).  Mean age = 10.9 years (age range = 9-13 years).  USA.  SCENE study. | 9-month, double-blind, BS, RCT.    1) Active (whole egg powder)  2) Control (milk powder)  3) Control (placebo - gelatine powder).  Powders were added to the following meals: breakfast (waffles or pancakes), dessert (brownies or ice-cream), and lunch/dinner (macaroni cheese). Ten foods were consumed p/w as a substitute for similar products in their normal diet. | Administered pre- and post-intervention:  NIH Toolbox Cognitive Battery (Dimensional Change Card Sort Test [mental flexibility], List Sorting Working Memory Test [working memory], Arrow Flanker test [selective attention and inhibition], Picture Sequence Memory Test [visual memory], and Pattern Comparison Processing Speed test [information processing speed]. Scores were combined to create a composite non-verbal cognition score). | N/A | N/A | Selective attention and inhibitory control improved from pre- to post-intervention in the milk powder group but not the placebo group. No changes were reported in the egg powder group |
| **Whole grains (n = 1)** | | | | | | |
| Chung et al. (2012) | N = 28 males.  Mean age = 16.21 years (age range = 15-17 years).  Korea.  1 school.  High SES.  Highly educated sample. | 9-week, single-blind (outcome assessors), BS, RCT.  1) Active (120g of germinated and non-germinated brown, polished, and black rice, kidney beans, and walnuts 3X p/d, providing 411 kcal per meal)  2) Control (120g of non-glutinous polished rice 3 times p/d, providing 417 kcal per meal). | Administered pre- and post-intervention:  Rapid Visual Information Processing test and AX-CPT (sustained attention); Computerized Neurocognitive Test-40 (Auditory-CPT [sustained attention], Digit Span Forwards and Backwards test [working memory], Stroop test [inhibition], TMT-A [information processing speed], TMT-B [mental flexibility], VLT [verbal memory], WCST [inhibition], and Thurstone Word-Fluency Test [verbal fluency]). | N/A | Measured pre- and post-intervention:  Haemoglobin, BDNF, and S100B. | Delayed verbal memory improved to a significantly larger extent in the control group than active group.  The number of correct responses on the AX-CPT decreased, and the number of omission errors increased, from pre- to post-intervention in the control group versus active group.  BDNF levels decreased from pre- to post-intervention in the control group and increased slightly in the active group. |
| **Nordic** **diet (n = 3)** | | | | | | |
| Sørensen et al., (2015a) | N = 739 (377 males, 362 females).  Mean age = 10 years (age range = 8-11.6 years).  Denmark.  9 schools (2 year groups per school).  Mixed SES.  OPUS School Meal Study. | 6-month, WS, cluster-RCT (3-months per intervention).  Clustered by year group within each school.  1) Active (an *ad libitum* lunch, a mid-morning snack, and an afternoon snack that followed the NND guidelines, providing 40-45% of recommended daily energy intake)  2) Control (habitual packed lunch and snacks from home). | Administered pre- and post-intervention periods:  D2 Test of Attention (attention and information processing speed). | Administered pre- and post- intervention periods:  Standard Danish tests (reading and mathematics). | Measured pre- and post-intervention periods:  Dietary intake (7-day food diary). | Average daily dietary intake of vegetables, fish, potatoes, fibre, protein, ferritin, folate, iodine, and vitamin D was significantly higher during the active period than control period, whereas intake of bread, whole grains, and fat (particularly saturated fat) was lower during the active period.  Higher percentage of errors of omission (inattention) and commission (impulsivity) after the active period than control period. No differences in attention and information processing speed.  Reading speed and accuracy was better after the active period than control period. However, reading accuracy was only better after the active period in those who received the control intervention first.  Exclusion of participants with ADHD (n = 14) did not affect the results. |
| Sørensen et al. (2015b) | See Sørensen et al., (2015a) | See Sørensen et al., (2015a) | See Sørensen et al., (2015a) | See Sørensen et al., (2015a) | Measured pre- and post-intervention periods:  Haemoglobin, ferritin, and omega-3 fatty acid composition. | Active intervention was associated with higher EPA, DPA, DHA, and EPA+DHA levels, and a lower ratio of *n*-6:*n*-3, compared to the control intervention. No changes in ferritin and haemoglobin were reported.  PCA identified 2 factors: ‘school performance’ and ‘reading comprehension’. The active intervention was associated with better ‘school performance’ and ‘reading comprehension’ versus the control intervention. |
| Sørensen et al. (2016) | See Sørensen et al., (2015a) | See Sørensen et al., (2015a) | See Sørensen et al., (2015a) | Administered pre- and post-intervention periods:  Standard Danish tests (mathematics and reading. Participants were divided into poor readers and normal/good readers at baseline). | N/A | At baseline, males were poorer at reading and more impulsive/inattentive than females. Increases in impulsivity following the active intervention were only present in males, whereas Improvements in reading abilities following the active intervention were more pronounced in males than females.  At baseline, those whose parents had an academic education were less impulsive/inattentive and had better reading scores. Increases in impulsivity and reading accuracy after the active intervention were only present in those whose parents had an academic education.  Increases in D2-error% following the active intervention were greater in those with normal/good baseline reading proficiency versus poor baseline reading proficiency. Improvements in reading accuracy were observed only in those with normal/good baseline reading proficiency. |
| **Multi-nutrient interventions (N = 11)** | | | | | | |
| Chellappa and Karunanidhi (2012) | N = 109 females.  Mean age = 18.44 years (age range = 17-19 years).  1 school.  India.  Mixed SES (low and middle-income families).  79% of the sample were ID at baseline (<12ug/L), of which 35% also had a zinc deficiency (70ug/L). | 4-month, double-blind, BS, RCT.  1) Active iron (184.6mg ferrous fumarate once p/d, providing 60mg elemental iron)  2) Active zinc (82.4mg zinc sulphate once p/d, providing 30mg elemental zinc)  3) Active iron + zinc (184.6mg ferrous fumarate and 82.4mg zinc sulphate, providing 60mg iron and 30mg zinc)  4) Control (placebo). | Administered pre- and post-intervention:  Digit Symbol Substitution Test (information processing speed); Raven’s Matrices test (non-verbal IQ - perceptual reasoning); Digit Vigilance test (attention); Rey Auditory VLT (verbal memory); Rey Complex Figure Test and PGI Memory Scale (visual memory). | N/A | Measured pre- and post-intervention:  Haemoglobin, ferritin, and zinc. | Ferritin levels increased in the iron group and iron + zinc group versus control group. Zinc levels significantly increased in the zinc group versus all other groups.  Information processing speed (accuracy) was better in all active groups compared to the control group.  Immediate and delayed visual memory significantly improved in the iron group and iron + zinc group versus control group. |
| Haskell et al. (2008) | N = 78 (35 males, 43 females).  Mean age = 11.05 years (age range = 8-14 years).  SES not reported.  UK. | 3-month, double-blind, BS, RCT.  1) Active (supplements providing 16 vitamins and minerals, including 1420IU vitamin A, 1g vitamin B1, 1.1g vitamin B2 and B6, 1.2g vitamin B12, 44mg vitamin C, 7.5ug/300IU vitamin D, 100ug folic acid, 5mg iron, 5mg zinc, and 25mg magnesium p/d)  2) Control (placebo). | Administered pre-, mid- (after 1 and 2 months), and post-intervention:  Internet Battery (Arrow Flankers test [selective attention and inhibition], Arrow RT test [information processing speed], PAL test [spatial memory], Sentence Verification test [semantic memory], Word List Recognition test [delayed verbal memory], and Picture Recognition test [delayed visual memory]). | N/A | N/A | Selective attention and information processing speed improved to a significantly larger extent in the active group than control group after 1 and 2 months. The beneficial effect of MVM supplementation on selective attention was also reported after 3 months. However, visual memory improved to a larger extent in the control group than active group. |
| Kalaichelvi (2021) | N = 240 females.    Age range = 12-15 years.  India.  4 schools (randomly selected).  Participants were IDA at baseline. | 2-month, BS, cluster-RCT.  Clustered by school.  1) Active (nutritional ball containing rice flakes, jaggery, and amla fruit powder, providing 13mg iron and 4g vitamin C 5 days p/w)  2) Control (habitual diet). | Administered pre- and post-intervention (immediately post-intervention and 5 to 6 weeks later).  Malin's Intelligence Scale for Indian Children, Indian adaptation of WISC (verbal and performance IQ). | N/A | Measured pre- and post-intervention (immediately post-intervention and 5 to 6 weeks later):  Haemoglobin. | Haemoglobin levels significantly improved in the active group, such that 40% had normal haemoglobin levels post-intervention compared to only 2.5% in the control group.  Verbal IQ scores were significantly higher in the active group than control group at both post-intervention follow-ups. |
| Lynn and Harland (1998) | N = 413.  Mean age = 13.1 years (age range = 12-15 years).  7 schools.  UK.  SES unknown.  3% and 17% of the sample were anaemic or ID at baseline, respectively. | 4-month, double-blind, BS, RCT.  1) Active (17mg elemental iron with 70mg vitamin c p/d).  2) Control (placebo). | Administered pre- and post-intervention:  Raven’s Matrices test (non-verbal IQ - perceptual reasoning). | N/A | Measured pre- and post-intervention:  Ferritin and haemoglobin (participants were divided into 3 groups based on ferritin levels: ID (<12ng/ml), moderate iron levels (12.1 – 20ng/ml), or high iron levels (>20.1ng/ml)). | When the whole sample was analysed together, the active group displayed a small, but non-significant, improvement in non-verbal IQ whilst the placebo group showed a small, but non-significant, decline in non-verbal IQ.  In those with an ID, the active group gained 3.07 non-verbal IQ points whilst the placebo group lost 2.7 IQ points, equating to a significant difference of 5.8 non-verbal IQ points. |
| Perlman et al. (2010) | N = 684 (305 males, 379 females).  Mean age = 10.95 years (age range = 8-12 years).  37 schools.  USA.  Low SES.  25% of sample did not meet the RDA for iron and folate and 93% for calcium. | 8-month, double-blind, BS, RCT.    1) Active (MVM supplement, providing 100% of US RDA for ages 4-12 years, except for calcium (12.5%), magnesium (18%), copper (50%), and iron (50%))  2) Control (placebo).  Supplements were not consumed on weekends, school holidays, or when students were absent from school. | N/A | Administered pre- and mid-intervention (4 months):  TerraNova (standardised test).  Measured pre- and post-intervention:  Academic grades (GPA - reading, language, mathematics, science, and social sciences). | N/A | No changes in TerraNova or GPA scores from pre- to post-intervention. |
| Petrova et al. (2019) | N = 103 participants (52 males, 51 females).  Mean age = 11.28 years (8-14 years).  3 schools.  Spain. | 5-month, double-blind, BS, RCT.  1) Active (0.6L of fortified Puleva Max milk p/d, providing 120mg DHA, 60mg EPA, 720mg vitamin A, 1.26mg vitamin B1, 1.44mg vitamin B2, 16.2mg vitamin B3, 0.9mg vitamin B12, 54mg vitamin C, 4.5mg vitamin D, 840mg calcium, and 13.5mg zinc)  2) Control (0.6L regular full milk p/d). | Administered pre-and post-intervention:  WISC-IV (Digit Span Backwards test and Letter-Number Sequencing test [working memory] and Coding and Symbol/Animal Search test [information processing speed]). | N/A | Measured pre-and post-intervention:  Lipid profiles, iron, ferritin, DHA, calcium, and vitamin D and E. | DHA increased by 28% in the active group from pre- to post-intervention but declined (-6%) in the control group. Vitamin D increased by 41% in the active group versus 21% in the control group.  Working memory (Digit Span test) improved by 32% in the active group versus 13% in the control group.  Vitamin D, but not DHA, levels were included in mediation analysis. Mediation analysis indicated that the small improvement in processing speed in the active group was mediated entirely by increases in vitamin D levels. |
| Schoenthaler et al. (1991) | N = 615.  Two age groups: 12-13 years and 15-16 years.  4 schools.  USA.  Mixed SES (1 low SES, 2 middle SES, and 1 high SES). | 13-week, double-blind, BS, cluster-RCT.  Clustered by school.  1) Active high dose (daily MVM supplements, providing 200% of US RDA for adults, excluding vitamin A and D, folic acid, biotin, and iodine [100%], copper [150%], and calcium and magnesium [20%])  2) Active medium dose (daily MVM supplements, providing 100% of US RDA for adults, excluding calcium and magnesium [20%])  3) Active low dose (daily MVM supplements, providing 50% of US RDA for adults, excluding calcium and magnesium [20%])  4) Control (placebo). | Administered pre- and post-intervention:  WISC-R (verbal and non-verbal IQ); MAT (non-verbal IQ - perceptual reasoning); RT/IT test (information processing speed).  Administered at 1 month:  Raven’s Matrices test (non-verbal IQ - perceptual reasoning). | Administered pre- and post-intervention:  CTBS (vocabulary, spelling, comprehension, reading, language mechanics and expression, mathematics comprehension and application, science, and social studies). | Measured pre- and post-intervention (data not reported):  Biochemical indices of micronutrient status. | Non-verbal IQ (Raven’s Matrices) was significantly better in the high dose group versus control group after 1 month.  Non-verbal IQ (WISC-R) significantly improved from pre- to post-intervention in the medium dose group versus control group. Larger gains when the analysis excluded those who complied for <50 days. |
| Sen and Kanani (2009) | N = 161 females.  Age range = 9 -13 years.  4 schools (randomly selected).  India.  Low SES.  68.3% of females were anaemic at baseline. | 12-month, BS, cluster-RCT.  Clustered by school.  1) Active high dose (daily iron-folic acid supplements, providing 100mg iron and 0.5mg folic acid p/d)  2) Active medium dose (daily iron-folic acid supplements, providing 28.57mg iron and 0.14mg folic acid p/d)  3) Active low dose (daily iron-folic acid supplements, providing 14.29mg iron and 0.07mg folic acid p/d)  4) Control (habitual diet). | Administered pre- and post-intervention:  WISC (Digit Span Forwards and Backwards test [working memory] and Maze test [planning abilities]); Visual Memory test [visual memory]; Clerical test [attention]). | N/A | Measured pre- and post-intervention:  Haemoglobin. | Haemoglobin increased in all active groups compared to the control group, with the medium dose group showing the largest increase in haemoglobin from pre- to post-intervention. Anaemic females showed larger improvements in haemoglobin than non-anaemic females following active supplementation.  Performance on all 4 cognitive tests was better in the high dose group and medium dose group versus the control group, whereas performance on only the Visual Memory test and Maze test was better in the low dose group versus control group.  . |
| Southon et al. (1994) | 51 participants (19 males, 32 females).  Mean age = 13.8 years (age range = 13-14 years).  2 schools.  UK. | 4-month, BS, non-RCT.  4 females were transferred to the control group after experiencing side effects from the active supplement. Researchers and data analysts were unaware of this change.  1) Active (daily MVM supplement, providing 100% of UK RDA or 100% of US RDA when no UK value was available [excluding vitamin A, E, and K])  2) Control (placebo). | Administered pre- and post-intervention:  WISC-R (verbal and non-verbal IQ). | N/A | Measured pre- and post-intervention:  Haemoglobin, iron, ferritin, zinc, copper, selenium, folic acid, and vitamin B1, B2, B6, B12, C, and D3. | The active supplement was associated with a larger increase in vitamin B12 and folic acid from pre- to post-intervention.  No changes in verbal and non-verbal IQ from pre- to post-intervention. |
| Snowden (1997) | N = 30.  Age range = 9-10 years.  1 school.  UK.  Mixed SES. | 10-week, triple-blind, BS, RCT.  1) Active (daily MVM supplement providing 25 vitamins and minerals, including 10ug vitamin B12, 3ug vitamin D, 50ug iodine, and 1.3mg iron)  2) Control (placebo). | Administered pre- and post-intervention:  Cognitive Abilities Test (verbal IQ); Calvert Non-Verbal Test (non-verbal IQ). | N/A | N/A | Non-verbal IQ scores increased to a larger extent from pre- to post-intervention in the active group than control group. |
| Wang et al. (2017) | N = 296 (81 males, 215 females).  Mean age = 13.3 years (age range = 12 - 14 years).  1 school.  China.  Low SES.  91% and 48% were vitamin B_2_ or selenium deficient at baseline, respectively. | 6-month, BS, cluster-RCT.  Clustered by class.  1) Active (250mL fortified milk containing vitamin A, D, E, B_2_, and B_5_, phosphorus, and zinc p/d)  2) Control (250mL unfortified pure milk p/d). | N/A | Administered pre- and post-intervention:    Academic grades (Chinese, mathematics, English, physics, social science, and ethics). | Measured pre- and post-intervention:  Ferritin, selenium, and vitamin D, B2, and B12 levels. | The prevalence of B2 and iron deficiency declined to a significantly larger extent after 6 months in the active group than control group.  The active group showed a larger improvement in Chinese, mathematics, English, and ethics scores from pre- to post-intervention than the control group. |
| **Iron (n = 12)** | | | | | | |
| Buzina-Suboticanec et al. (1998) | N = 50 (35 males, 25 females).  Mean age = 9.2 years (age range = 8.7-9.6 years).  Rural Croatia.  1 school (purposively selected due to high prevalence of mild anaemia).  40% and 31% of the sample were ID or vitamin B2 deficient, respectively, at baseline. | 10-week, double-blind, BS, cluster-RCT.  Clustered by class.  1) Active (100mg ferri-glycine sulphate 6 days p/w)  2) Control (placebo). | Administered pre- and post-intervention:  WISC-R (coding test [information processing speed], Block Design test and Picture Completion test [non-verbal IQ – perceptual reasoning], Digit Span test [working memory], Similarities test [verbal comprehension], and Arithmetic test [working memory]. Scores were combined to determine verbal, non-verbal, and full-scale IQ). | N/A | Measured pre- and post-intervention:  Transferrin saturation, iron concentration, zinc, and vitamin A, B2, E, and C. | Haemoglobin and transferrin saturation significantly improved in the active group and remained the same in the control group. Vitamin C and E significantly increased in both groups after 10 weeks, which was attributed to the seasonal change in dietary intake of vitamin C and E at the end of spring.  Information processing speed, non-verbal IQ, and full-scale IQ significantly improved in the active group after 10 weeks. No changes were reported in the control group.  When baseline haemoglobin status was considered, iron supplementation only improved full-scale and non-verbal IQ scores in those with lower haemoglobin levels at baseline. No changes were observed in those with higher baseline haemoglobin levels. |
| Bruner et al. (1996) | N = 81 females.  Mean age = 16 years (age range = 13-18 years).  4 schools.  USA.  Mixed SES.  Participants were ID at baseline. | 2-month, double-blind, BS, RCT.  1) Active (1300mg ferrous sulphate p/d)  2) Control (placebo). | Administered pre- and post-intervention:  Brief Test of Attention (attention); Symbol Digit Modalities Test (information processing speed); Visual Search and Attention Test (attention); Hopkins VLT (verbal memory). | N/A | Measured pre- and post-intervention:  Haemoglobin and ferritin. | The active group had significantly higher haemoglobin and ferritin levels post-intervention than the control group.  Immediate verbal memory improved to a larger extent in the active group than control group. |
| Devaki et al. (2008) | N = 120 (60 males, 60 females).  Age range = 15-18 years.  India.  25% of the sample were IDA, and 25% were ID, at baseline. | 8-months, BS, non-RCT.  IDA and ID group:  1) Active (Iron(III)-hydroxide polymaltose complex, providing 100mg elemental iron 6 days p/w).  Normal iron status group:  1) Active (Iron(III)-hydroxide polymaltose complex, providing 100mg elemental iron 6 days p/w)  2) Control (placebo). | Administered pre-, mid- (4-months), and post-intervention:  Short Term Memory test (immediate verbal memory); Long Term Memory test (delayed verbal memory); Raven’s Matrices test (non-verbal IQ - perceptual reasoning); WAIS (full-scale IQ). | Administered pre-, mid- (4-months), and post-intervention:  Mathematics test. | Measured pre-, mid- (4-months), and post-intervention:  Haemoglobin and ferritin. | Haemoglobin and ferritin levels significantly improved in all 3 active groups after 8 months, whereas the control group remained the same.  All active groups, especially the IDA and ID group, showed significant improvements in verbal memory, non-verbal IQ, full-scale IQ, and mathematics test scores after 4- and 8-months (except for immediate verbal memory in the non-anaemic ID group). No improvements were reported in the control group. |
| Kashyap and Gopaldas (1987) | N = 130 females.  Mean age = 9.76 years (age range = 8-15 years).  4 schools (purposively selected).  India.  Low SES.  90% of the sample were anaemic at baseline (primarily ID type). | 2-month, BS, RCT (repeated twice within a school year i.e. at the start of the first and second school term).  1) Active (324mg ferrous sulphate p/d)  2) Control (placebo). | Administered pre- and post-intervention (immediately after each 2-month intervention period, and 4 months after the second intervention period):  WISC (Digit Span Forwards and Backwards test [working memory] and Maze test [planning]); Clerical test (attention); Visual Memory test (visual memory). | N/A | Measured pre- and post-intervention (immediately post-intervention, and 4 months later):  Haemoglobin. | In the active group, the prevalence of anaemia declined from 75% to 25% during the first school term, and 25% to 1% during the second school term. No differences were observed in the placebo group. However, the prevalence of anaemia in the active group returned almost to baseline levels at the post-intervention assessment.  There was a significantly larger improvement in working memory, planning abilities, and attention at the end of the second school term in the active group compared to the control group. |
| Karkada et al. (2019) | N = 60 females.  Age range = 11-17 years.  India.  Low SES.  50% of the sample were mildly anaemic at baseline (type unspecified). | 3-month, BS, cluster, non-RCT.  Clustered by haemoglobin status.  Mildly anaemic females:  1) Active (1 to 2 tablespoons of *ragi* powder twice p/d, providing an estimated 0.58-2.21mg iron p/d).  Non-anaemic females:  1) Control (habitual diet). | N/A | Measured pre- and post-intervention:  Academic grades (subjects not specified). | Measured pre-, mid- (45 days), and post-intervention:  Haemoglobin. | Haemoglobin levels significantly improved from pre- to post-intervention in the active group but not control group (80% of participants in the active group were no longer classed as anaemic post-intervention, whereas 20% of the control group became mildly anaemic by the end of the intervention).  No changes in academic grades. |
| Lambert et al. (2002) | N = 116 females.  Mean age = 15.2 years (age range = 12.5-17.9 years.  New Zealand.  1 school.  Participants were ID at baseline. | 2-month, double-blind, BS, RCT.  1) Active (325mg ferrous sulphate p/d)  2) Control (placebo). | Administered pre- and post-intervention:  Hopkins VLT (verbal memory); Stroop test (inhibition); Visual Search test (attention); Reading Span test (working memory). | N/A | Measured pre- and post-intervention:  Ferritin and haemoglobin. | Ferritin levels increased to a significantly larger extent in the active group than placebo group. Haemoglobin levels significantly decreased from pre- to post-intervention in the control group and remained unchanged in the active group.  Immediate verbal memory significantly improved from pre- to post-intervention in the active group but not control group. However, this effect was observed only for words recalled from the second half of the list.  Irrespective of treatment group, the change in haemoglobin levels was associated with the improvement in immediate recall of recently heard words, and the change in ferritin levels was associated with improvements in working memory. |
| Pollitt, Soemantri et al. (1985); Pollitt (1997) | N = 68.  Mean age = 9.62 years (age range = 8-11 years).  Egypt.  Low SES.  41% of the sample were IDA at baseline. | 4-month, double-blind, BS, RCT.  1) Active (50mg ferrous sulphate 6 days p/w)  2) Control (placebo). | Administered pre- and post-intervention:  CPT (attention); Peabody Picture Vocabulary Test (verbal IQ); Matching Familiar Figure Test (impulsivity). | N/A | Measured pre- and post-intervention:  Ferritin and haemoglobin. | Haemoglobin levels significantly increased from pre- to post-intervention in the IDA group, irrespective of treatment group. Conversely, haemoglobin levels declined from pre- to post-intervention in those without IDA treated with the placebo.  In those with IDA, impulsivity scores significantly improved in the active group compared to the control group. No differences in those without IDA. |
| Pollitt et al. (1989) | N = 1358.  Age range = 9-12 years.  16 schools.  Thailand.  Low SES.  7.4% and 3.5% of the sample were IDA or ID at baseline, respectively. | 4-month, double-blind, BS, RCT.  1) Active (50mg ferrous sulphate p/d for 2 weeks, followed by 100mg p/d for 14 weeks)  2) Control (placebo).  Simultaneously administered active/placebo supplements with deworming tablets at the start of trial. | Administered pre- and post-intervention:  Raven’s Matrices test (non-verbal IQ -perceptual reasoning). | Measured pre- and post-intervention:  Educational Achievement Test (language and mathematics). | Measured pre- and post-intervention:  Ferritin and haemoglobin. | In the IDA group, haemoglobin levels increased in both the active and control group, but to a larger extent in the active group. Haemoglobin levels also increased slightly in the iron-treated ID group.  No changes in non-verbal IQ and Educational Achievement Test scores. |
| Rezaeian et al. (2014) | N = 200 females.  Mean age = 16.2 years (age range = 14-18 years).  1 school (randomly selected).  Iran.  20% of the sample were anaemic at baseline (type unspecified). | 4-month, single-blind (outcome assessor and data analysts), BS, cluster-RCT.  Clustered by school shift.  1) Active (50mg ferrous sulphate twice p/w)  2) Control (habitual diet). | Administered pre- and post-intervention:  Toulouse-Piéron test (attention). | N/A | Measured pre- and post-intervention:  Haemoglobin. | Haemoglobin levels significantly increased in the active group from pre- to post-intervention and declined in the control group.  There was a significantly larger increase in attention scores from pre- to post-intervention in the active group than control group. |
| Scott et al. (2018) | N = 140 (75 males, 65 females).  Mean age = 13.7 years (age range = 12-16 years).  1 school (purposively selected).  India.  Low SES.  50% and 33% of the sample were ID or anaemic at baseline, respectively. | 6-month, double-blind, BS, RCT.  1) Active (200-300g dried iron-biofortified pearl millet consumed as a midday and evening meal, providing 86ppm of iron)  2) Control (200-300g non-fortified pearl millet consumed as a midday and evening meal, providing 21ppm of iron for the first 4 months, and 52ppm for the remaining 2 months). | Administered pre- and post-intervention:  Simple RT test (information processing speed); Attention Network Test (attention); Cued Recognition test (visual memory); Go/No-Go test (inhibition). | N/A | Measured pre-, mid- (4-months), and post-intervention:    Haemoglobin, ferritin, and body iron. | Ferritin and body iron increased to a significantly larger extent mid-intervention in the active group than control group.  Performance on all tasks significantly improved to a larger extent in the active group than control group. RTs on the Simple RT test, Go/No-Go test, and Attention Network Test decreased two-fold from pre- to post-intervention in the active group versus control group. |
| Soemantri, Pollitt, & Kim,  (1985) | N = 119.  Mean age = 10.9 years.  3 schools (purposively selected).  Indonesia.  Low SES.  66% of the sample were IDA at baseline. | 3-month, double-blind, BS, RCT.  1) Active (10mg ferrous sulphate p/kg of body weight p/d)  2) Control (placebo). | Administered pre- and post-intervention:  Bourden-Wisconsin test (attention); Ravens Matrices test (non-verbal IQ - perceptual reasoning). | Administered pre- and post-intervention:  Educational Achievement Test (biology, mathematics, social science, and language). | Measured pre- and post-intervention:  Haemoglobin. | In those with IDA, haemoglobin levels significantly increased from pre- to post-intervention in the active group and declined slightly in the control group. Haemoglobin levels were unchanged in the non-anaemic group.  In those with IDA, the active group showed a significantly larger improvement in Educational Achievement Test scores from pre- to post-intervention than the control group. However, post-intervention scores in the non-IDA group were still higher than the IDA group treated with iron. |
| Soemantri et al. (1989) | N = 130.  Mean age = 10.4 years.  2 schools (purposively selected).  Indonesia.  Low SES.  45% of the sample were IDA at baseline. | 3-month, double-blind, BS, RCT.  1) Active (10mg ferrous sulphate p/kg of body weight p/d)  2) Control (placebo). | Administered pre- and post-intervention (immediately post-intervention and 3 months later):  Raven Matrices test (non-verbal IQ – perceptual reasoning). | Administered pre- and post-intervention:  Educational Achievement Test (biology, mathematics, social science, and language). | Measured pre- and post-intervention:  Haemoglobin. | Haemoglobin levels in the IDA group treated with iron significantly improved from pre- to post-intervention and were equivalent to those without IDA immediately post-intervention and 3 months later.  No changes in non-verbal IQ were reported. Differences in Education Achievement Test scores between groups were not statistically analysed. |
| **Iodine (n = 4)** | | | | | | |
| Gordon et al. (2009) | N = 184 (101 males, 83 females).  Mean age = 11.2 years (age range = 10-13 years).  2 schools  New Zealand.  Low SES.  Participants were mildly iodine deficient at baseline (UIC = 63 μg/L), but had normal thyroid status. | 6-month, double-blind, BS, RCT.  1) Active (0.15mg iodine p/d)  2) Control (placebo). | Administered pre- and post-intervention:  WISC-IV (Letter-Number Sequencing test [working memory], Picture Concepts and Raven’s Matrices test [non-verbal IQ - perceptual reasoning], and Symbol Search test [information processing speed]). | N/A | Measured pre- and post-intervention:  Total thyroxine and UIC. | UIC increased in the active group post-intervention, reflecting normal iodine status. There was a small improvement in iodine status in the control group, but levels remained within the mildly deficient range.  Non-verbal IQ improved from pre- to post-intervention in the active group versus placebo group. No changes in working memory or information processing speed were reported. |
| Huda et al. (2001) | N = 305.  Mean age = 9.8 (age range = 8-10 years).  2 severely iodine deficient rural areas in Bangladesh.  Participants were moderately iodine deficient at baseline according to UIC (3.2μmol/L) and severely deficient according to goitre prevalence (>95% goitre rate). | 4-month, double-blind, BS, RCT.  1) Active (single dose of 400mg iodized poppy seed oil capsule [Lipiodol])  2) Control (placebo containing poppy seed oil). | Administered pre- and post-intervention:  Verbal Fluency task (verbal fluency); Digit Span Backwards test (working memory); Visual Search task (attention); French PAL task (verbal memory); Coris Blocks test (spatial working memory); Raven’s Matrices test (non-verbal IQ - perceptual reasoning); Symbol Digit Modalities Test (information processing speed); Modified Stroop test (inhibition). | N/A | Measured pre- and post-intervention:  Total thyroxine, TSH, and UIC | Although there was a significantly larger increase in UIC in the active group compared to the control group, 85% of the sample had UIC indicative of a mild deficiency in the active group.  No changes in cognition. |
| Isa et al. (2000) | N = 165 (90 males, 75 females).  Mean age = 11.38 years.  4 moderately to severely iodine deficient villages in Malaysia (purposively selected).  Participants were severely iodine deficient at baseline (UIC = 15.5μg/L). | 12-month, BS, non-RCT.  Clustered by village.  1) Active (single dose of iodized poppyseed oil capsules [Lipiodol]. Dose based on manufacturers recommendations)  2) Control (habitual diet). | Administered pre-, mid- (6 months), and post-intervention:  Test of Non-Verbal Intelligence (non-verbal IQ – percentile and quotient [6 scores, ranging from ‘very poor’ to ‘superior’]). | N/A | Measured pre- and post-intervention:  Thyroid volume and UIC. | UIC increased significantly in both groups after 12 months, with the active group showing a larger improvement. Thyroid volume significantly declined in both groups after 6 months. However, after controlling for the higher thyroid volume in the control group at baseline, the active group showed a larger reduction in thyroid volume.  The number of participants who scored above 3 percentiles significantly increased after 12 months in the active group, and after 6 months in the control group.  The maximum score obtained in the control group after 6 and 12 months was the ‘good’ score and ‘superior’ score, respectively. In contrast, the maximum score obtained in the active group after 6 and 12 months was the ‘average’ score and ‘good’ score, respectively. |
| Zimmermann et al. (2006) | 310 participants (166 males, 144 females).  Mean age = 11.4 years (age range = 10-12 years).  7 schools.  Severely iodine deficient areas in Albania.  Participants were moderately to severely iodine deficient at baseline (goitre rate = 87% and median UIC = 44μg/L). | 6-month, double-blind, BS, RCT.  1) Active (single dose of 400mg iodized poppyseed oil capsules [Lipiodol])  2) Control (placebo containing sunflower oil). | Administered pre- and post-intervention:  WISC-III (Coding test, Symbol Search test, Rapid Object Naming test, and Rapid Target Marking test [information processing speed], Raven’s Matrices test [non-verbal IQ -perceptual reasoning], and Digit Span Forwards and Backwards test [working memory]). | N/A | Measured pre- and post-intervention:  TSH, total thyroxine, thyroid gland volume, and UIC. | UIC, thyroid volume, and total thyroxine levels significantly improved in the active group. No changes in iodine status occurred in the control group.  Information processing speed (Symbol Search test, Rapid Object Naming test, and Rapid Target Marking test) and non-verbal IQ improved to a significantly larger extent in the active group compared to the control group. |
| **Vitamin D (n = 1)** | | | | | | |
| Grung et al.  (2017) | 50 participants (18 males, 32 females).  Age range = 13-14 years.  2 schools.  Norway.  Participants were vitamin D deficient or insufficient at baseline (<50 nmol/L). | 3-month, double-blind, BS, RCT.  1) Active (38μg vitamin D p/d)  2) Control (placebo).  Conducted during winter, when vitamin D levels are typically low. | Administered pre- and post-intervention:  Tower of Hanoi and Tower of London (problem solving). | N/A | Measured pre- and post-intervention:  Vitamin D. | Vitamin D levels increased significantly from pre- to post-intervention in the active group only. Post-intervention vitamin D levels were within the normal range in the active group but remained in the deficient/insufficient range in the control group.  Performance significantly improved on the difficult levels of the Tower of Hanoi test from pre- to post-intervention in the active group but not the control group. No changes in performance on the easy levels of the Tower of Hanoi or both levels of the Tower of London. |
| **Polyphenols (n = 2)** | | | | | | |
| Nidich et al. (1993) | N = 34  Mean age = 8.72 years (age range = 8-9 years).  USA. | 5-month, BS, double-blind, RCT.  A) Active (Maharishi Ayur-Ved Student Rasayana [dose not reported])  B) Control (placebo). | Administered pre- and post-intervention:  Form A of the Cattell Culture Fair Intelligence Test (non-verbal IQ). | N/A | N/A | The active group exhibited a significantly larger improvement in non-verbal IQ points after 5 months than the placebo group. |
| Tefagh et al. (2022) | N = 86 females.  Mean age = 16.3 years (age range = 15-17 years).  4 schools (randomly selected).  Iran.  Average SES. | 6-week, double-blind, BS, RCT.  1) Active (250mg *Ustukhuddus Alavi* p/d, containing fern, lavender, grapes, roots of pyrethrum and peony, and fruits of Cuscuta epithymum)  2) Control (placebo). | Administered pre-, mid- (3 weeks), and post-intervention (immediately post-intervention, and 1 month later):  Paced Auditory Serial Addition Test (working memory, sustained attention, and information processing speed). | N/A | N/A | Working memory was better in the active group than control group after 3 weeks, whereas sustained attention was better in the active group after 3 and 6 weeks. |
| **School breakfast programs (n = 3)** | | | | | | |
| Cueto and Chinen (2008) | N = 590 (302 male, 288 female).  Mean age = 11.87 years.  Peru.  20 schools.  Low SES.  Full-grade and mixed-grade schools. | 3-year, BS, clustered, non-RCT.  Clustered by school.  Government funded SBP.    1) Active (breakfast at home followed by a mid-morning school breakfast consisting of 1 cup of lactose-free milk and 6 biscuits, providing 600 kcal, 22.5g protein, 20g fat, 100% RDA iron, and 60% RDA of several vitamins and minerals)  2) Control (breakfast at home). | Administered approximately 3 years after the SBP was implemented:  Picture Recognition test (visual memory); WISC-R (Coding test [information processing speed]). | Administered post-intervention only:  Unstandardised mathematics and Spanish reading comprehension tests. | N/A | Picture recognition, mathematics, and reading comprehension scores were significantly better in multiple-grade active schools than multiple-grade control schools at post-intervention. Mathematics and reading comprehension scores were poorer in full-grade active schools than full-grade control schools.  No differences in information processing speed between schools. |
| Murphy et al. (2011) | N = 4350.  Age range = 9-11 years.  111 schools.  UK.  Mixed SES. | 12-month, single-blind (data analysts), BS, cluster-RCT.  Clustered by school.  Government funded SBP.  1) Active (Primary School Free Breakfast Initiative, included a milk-based drink or product, low sugar cereal, fruit, and bread).  2) Control (waitlist). | Administered pre- and post-intervention in a subsample of participants:  Word List Recall test (verbal memory). | N/A | N/A | The number of healthy breakfast items consumed in active schools significantly improved after 12 months. No differences in verbal memory between groups. SES of school did not affect results. |
| Shemilt et al.  (2004) | N = 6042 (3032 males, 3010 females).  Mean age = 9.86 years.  UK.  27 schools.  Low SES. | 12-month, BS, cluster-RCT (only analysed data from 3-month follow-up as schools became contaminated).  Clustered by school.  Government funded SBP.  1) Active (funding for SBP. Breakfast composition was chosen by each school)  2) Control (no funding). | Administered pre-, mid- (3 months), and post-intervention:  TMT-A [information processing speed]; TMT-B [mental flexibility/shifting]. | N/A | N/A | Post-intervention data were not analysed due to contamination in the control group.  Information processing speed was significantly faster in the active group than control group after 3 months. |

**Abbreviations**: ALA = alpha-linolenic acid, AA = arachidonic acid, BS = between subjects, CPAL = continuous paired associative learning, CPT = continuous performance task, DHA = docosahexaenoic acid, DPA = Docosapentaenoic*,* EPA = eicosapentaenoic acid, fMRI = functional magnetic resonance imaging, IQ = intelligence quotient, LA = linoleic acid, IDA = iron deficiency anaemia, ID = iron deficiency, MAT = Matrix Analogies Test, ObA = osbond acid, p/d = per day, p/w = per week, PAL = paired associate learning, PCA = principal component analysis, RCT = randomised controlled trial, RDA = recommended daily allowance, RT = reaction time, SBP = school breakfast programme, SES = socioeconomic status, TMT = trail making task, TSH = thyroid stimulating hormone, UIC = urinary iodine concentration, VLT = verbal learning test, WISC = Wechsler Intelligence Scale for Children, WS = within subjects.

**Table S10.** Longitudinal studies examining the effect of diet quality during infancy (<3 years) on adolescent outcomes.

| **Author**  **(year)** | **Sample characteristics** | **Dietary measure or intervention** | **Outcome measures** | | | **Results** |
| --- | --- | --- | --- | --- | --- | --- |
|  |  |  | **Cognitive measures** | **Academic measures** | **Biochemical and neurological measures** |  |
| **General diet quality (n = 10)** | | | | | | |
| Feinstein et al.(2008) | N = 5471 participants with dietary data at 1 year and academic grades at 10-11 years.  Predominantly middle SES.  UK.  ALSPAC cohort. | Administered at 3 years_1_:  43-item FFQ completed by primary caregiver (factor analysis identified 3 dietary patterns: *junk food*, *health-conscious*, and *traditional*). | N/A | Administered between 10-11 years:  Standardised academic tests (English, mathematics, and science grades combined via factor analysis). | N/A | Participant covariates: singleton or multiple birth, sex, ethnicity, birth weight, number of siblings at birth, dietary patterns at age 4 and 7, and television use between 18-42 months. Maternal covariates (obtained prior to or between 3-4 years): employment status, relationship status, job skill, difficulties with affording food, age at time of birth, education at time of birth, smoking status during pregnancy, breastfeeding, household weekly income, housing tenure, HOME score (index of cognitive stimulation and emotional warmth in home environment), and intake of vegetarian diet when infant was born.  Negative association between *junk food* dietary pattern at 3 years and average academic grades at 10-11 years, which weakened but remained significant after controlling for confounding variables.  Positive association between *health*-*conscious* dietary pattern at 3 years and average academic grades at 10-11 years. This was no longer significant after controlling for confounding variables. |
| Golley et al. (2013) | N = 4429 participants with dietary data at 6 months and IQ scores at 8.5 years.  Predominately middle SES.  UK.  ALSPAC cohort. | Administered at 6 months:  FFQ completed by primary caregiver (Complementary Feeding Utility Index score based on adherence to current infant feeding practices including breastfeeding duration, age at solid food introduction, and exposure to iron-rich, textured, ready-made, and high fat, salt, and sugar foods). | Administered at 8.5 years:  WISC-III (full-scale, verbal, and performance IQ). | N/A | N/A | Maternal covariates (obtained during pregnancy): maternal age, ethnicity, pre-pregnancy BMI, education, occupation, marital status, number of children <16 years in family home, and tobacco use. Participant covariates: birth weight, sex, dietary patterns at 7 years, gestational age at birth, stimulation in home, and singleton or multiple birth. Maternal IQ at 15 years (n = 1776).  After adjusting for all covariates, other than maternal IQ, a higher index score at 6 months was positively associated with total, verbal, and performance IQ scores at 8.5 years. However, when the analysis was additionally adjusted for maternal IQ, the association between index scores and performance IQ was no longer significant. |
| Mou et al. (2023) | N = 1888 participants with dietary data at 1 year and full-scale IQ scores at 13 years (914 male, 974 female).  Mostly middle SES.  Netherlands.  Generation R Study. | Administered at 1 year:  221-item FFQ completed by primary caregiver (Diet Quality Score based on adherence to Dutch dietary guidelines for 10 food groups. PCA identified 3 dietary patterns: *vegetables, potatoes, and grains*, *snacks, processed foods, and sugars*, and *butter, margarines, whole grains, and dairy*. | Administered at 13 years:  WISC-V (full-scale IQ). | N/A | Measured at 10 years:  Brain morphology (MRI scan). | Participant covariates: sex, age at neuroimaging assessment, ethnicity, energy intake at 1 and 8 years, and BMI at 10 years. Maternal covariates (obtained during pregnancy): maternal education, psychopathology symptoms, alcohol use, tobacco use, folic acid supplement use, diet quality, and household income.  No direct relationship between *snacks, processed foods, and sugars* dietary pattern at one year and full-scale IQ at 13 years.  Negative association between *snacks, processed foods, and sugars* dietary pattern at 1 year and cerebral white matter volume at 10 years.  Association between *snacks, processed foods, and sugars* dietary pattern at 1 year and full-scale IQ at 13 years was mediated by cerebral white matter volume at 10 years. |
| Northstone et al. (2012) | N = 7044 participants with dietary data at 3 years and IQ scores at 8.5 years (3516 males, 3528 females).  Predominately middle SES.  UK.  ALSPAC cohort. | Administered at 3 years_2_:  FFQ completed by primary caregiver (PCA identified 4 dietary patterns: *processed* (high fat and/or sugary processed/convenience foods), *traditional* (meat, potatoes, and vegetables), *health-conscious* (vegetables, fruits, fish, pasta, and rice), and *snack* (finger foods such as fruits, biscuits, and cakes). | Administered at 8.5 years:  WISC-III (full-scale, verbal, and performance IQ). | N/A | N/A | Maternal covariates (obtained during pregnancy): maternal education, housing tenure, oily fish intake, and SES. Participant covariates: sex, age at WISC-III assessment, WISC-III administrator, number of stressful life events experienced throughout childhood, breastfeeding duration, energy intake at home at 18 months, maternal age at birth, and dietary pattern scores at 3, 4, 7, and 8.5 years.  After adjusting for covariates, there was a negative association between a *processed* dietary pattern at 3 years and full-scale IQ at 8.5 years. There was also a positive association between a *snack* dietary pattern at 3 years and full-scale IQ at 8.5 years. The positive association between a *health-conscious* dietary pattern at 3 years and full-scale IQ at 8.5 years was no longer significant after adjusting for covariates. |
| Nyaradi et al. (2013) | N = 1346 participants with dietary data at 1 year and verbal IQ scores at 10 years (691 males, 655 females).  N = 1455 participants with dietary data at 1 year and non-verbal IQ scores at 10 years (752 males, 703 females).  Mixed SES.  Australia.  Raine study cohort (Generation 2). | Administered at 1, 2, and 3 years:  24-hr food recall questionnaire completed by primary caregiver (EAT diet score based on Dietary Guidelines for Children and Adolescents in Australia. Higher scores reflected a more frequent intake of wholegrains, vegetables, fruits, dairy, white meat, and legumes, and a less frequent intake of red and processed meats, snack foods, and sweetened beverages). | Administered at 10 years:  Peabody Picture Vocabulary Test-III (verbal IQ); Raven’s Matrices test (non-verbal IQ - perceptual reasoning). | N/A | N/A | Maternal covariates (obtained during pregnancy): maternal age and education. Maternal mental health distress (at 3 years). Participant covariates: family income (at 3 years), sex, biological father living with family (at 3 years), cognitive/language stimulation at home (at 3 years), and breastfeeding duration.  Positive association between EAT scores at 1 year and verbal and non-verbal IQ at 10 years, after adjusting for covariates. Diet scores at 2 and 3 years were significant predictors of non-verbal IQ at 10 years, but the relationship was no longer significant after adjustment for covariates.  After adjusting for covariates, sweetened beverage intake at 1 year was negatively associated with verbal and non-verbal IQ at 10 years. Fruit intake at 1 year was positively associated with verbal IQ at 10 years. Dairy intake at 2 and 3 years was positively associated with verbal IQ scores at 10 years, whilst dairy intake at 2 years was positively associated with non-verbal IQ at 10 years. |
| Nyaradi et al. (2015) | N = 717 participants with dietary data at 1 year and cognitive data at 17 years (male, female).  Mixed SES.  Australia.  Raine study cohort (Generation 2). | See Nyaradi et al. (2013) | Administered at 17 years:  CogState (Detection and Identification task [information processing speed], One Card Learning test [visual memory], and CPAL test [spatial memory]). | N/A | N/A | Maternal covariates (obtained during pregnancy): maternal age, race, and education. Maternal mental health distress (at 3 years). Participant covariates: family income (at 3 years), sex, biological father living with family (at 3 years), cognitive/language stimulation at home (at 3 years), and breastfeeding duration.  Positive association between EAT scores at 1 year and faster information processing speed at 17 years. No association with spatial and visual memory. |
| Nyaradi et al. (2016) | N = 2287 participants with dietary data at 1 year and academic data at 10 and 12 years.  Mixed SES.  Australia.  Raine study cohort (Generation 2). | See Nyaradi et al. (2013) | N/A | Administered at 10 and 12 years:  Western Australian Literature and Numeracy Assessment (mathematics, reading, writing, and spelling). | N/A | Maternal covariates (obtained during pregnancy): maternal age and race. Maternal education (at 8 years). Participant covariates: sex, presence of biological father in family (at 1, 2, and 3 years), family income (age 1, 2, and 3 years), breastfeeding duration, and cognitive/language stimulation at home.  The following associations remained after controlling for confounding factors:  Positive association between EAT scores at 1 year and reading, writing, spelling, and mathematics scores at 10 years and reading, spelling, and mathematics scores at 12 years. Positive association between EAT scores at 2 years and mathematics, writing, and spelling scores at 12 years. Positive association between EAT scores at 3 years and mathematics scores at 12 years. Positive association between fruit intake at 1 year and mathematics and reading scores at 10 and 12 years. Positive association between dairy intake at 1, 2, and 3 years and all academic measures at 10 and 12 years. |
| Smithers et al. (2012) | N = 7052, 5610, and 6366 participants with dietary data at 6, 15, and 24 months, respectively, and cognitive data at 8.5 years.  Predominately middle SES.  UK.  ALSPAC cohort. | Administered at 6, 15, and 24 months:  43-item, 70-item, and 72-item FFQ completed by primary caregiver (PCA identified 3 similar dietary patterns at all ages: *homemade* *traditional* [meat, vegetables, and puddings], *ready-to-eat* [ready-made baby foods at 6 and 15 months, and cereal, yoghurt, milk pudding, and bread at 24 months], and *discretionary* [sweet and salty processed snacks and soft drinks]. At 6 months, a *breastfeeding* dietary pattern was also identified, and a *contemporary* dietary pattern at 15 and 24 months [herbs, legumes, nuts, and raw fruits and vegetables]). | Administered at 8.5 years:  WISC-III (full-scale, verbal, and performance IQ). | N/A | N/A | Covariates: obtained during pregnancy (maternal age, education, SES, marital status, tobacco smoking, family income, parity, ethnicity and number of children (<16 years old) living in the family home), sex, gestational age at birth, birth weight, singleton or multiple birth, stimulation at home, other dietary pattern scores at each age, and other dietary patterns at previous age.  The following associations were significant after adjusting for all covariates:  Negative association between a *discretionary* dietary pattern at 6 months and all IQ scores at 8.5 years. Negative association between a *discretionary* dietary pattern at 15 months and full-scale and verbal IQ at 8.5 years. Negative association between a *discretionary* dietary pattern at 24 months and full-scale IQ at 8.5 months. Positive association between a *traditional* dietary pattern at 6 months, but not 15 and 24 months, and all IQ scores at 8.5 years. Positive association between a *breastfeeding* dietary pattern at 6 months and full-scale and verbal IQ at 8.5 years. Positive association between a *contemporary* dietary pattern at 15 and 24 months and both full-scale and verbal IQ scores at 8.5 years. A *ready-to-eat* dietary pattern at 6 and 15 months was negatively associated, and at 24 months positively associated, with both full-scale and verbal IQ at 8.5 years. |
| Smithers et al. (2013) | N = 7652 participants with dietary data between 6 – 24 months and cognitive data at 8 and/or 15 years (3799 males, 3853 females).  Predominately middle SES.  UK.  ALSPAC cohort. | Administered at 6, 15, and 24 months:  43-item, 70-item, and 72-item FFQ completed by primary caregiver (PCA identified 4 dietary patterns: *healthy* [breastfeeding at 6 months, and raw fruit/vegetables, dairy, and herbs at 15 and 24 months], *discretionary* [sweet and salty processed snacks at all ages], *traditional* [meat, cooked vegetables, and puddings at all ages], and *ready-to-eat* [ready-made baby foods at 6 and 15 months, and biscuits, bread, and cereals at 24 months]). | Administered at 8 years:  WISC-III (full-scale, verbal, and performance IQ).  Administered at 15 years:  WASI (full-scale IQ). | N/A | N/A | Covariates: obtained during pregnancy (maternal education, occupation, marital status, and alcohol/tobacco use), family income at 33 months, number of children <16 years of age living in family home at 6 months, stimulation in family home at 18 months, sex, ethnicity, gestational age at birth, birth weight, maternal age at birth, maternal parity, singleton or multiple birth, breastfeeding duration, maternal IQ at 15 years, dietary pattern scores at 6, 15, and 24 months, dietary pattern scores at 3 years, and pre-pregnancy BMI.  Positive association between a ‘healthy’ dietary pattern trajectory during infancy and full-scale and verbal IQ at 8 years but not 15 years.  Negative association between a ‘traditional’ or ’discretionary’ dietary pattern trajectory and full-scale IQ at 15 years but not 8 years. |
| Zhu et al. (2020) | N = 745 participants with dietary data between 6 – 23 months and cognitive data between 10 – 12 years (450 males, 295 females).  Infants of mothers who participated in a RCT on prenatal micronutrient supplementation.  Mixed SES.  Rural China.  Excluded infants who were not breastfed. | Administered at 6, 9, 12, 18, and 24 months:  FFQ completed by primary caregiver (composite feeding score based on adherence to WHO complementary feeding guidelines, including breastfeeding duration, use of MVM supplements, timing of complementary food introduction [beans, eggs, and milk], and intake of Fe-rich or Fe fortified foods. Also assessed the effect of the timing of complementary food introduction). | Administered between 10 – 12 years:  WISC-IV (full-scale and non-verbal IQ, verbal comprehension, working memory, and information processing speed). | N/A | N/A | Covariates: obtained during pregnancy (parental age, occupation, household income, and education, and maternal mid-upper arm circumference), BMI (at 10-12 years), school type (at 10-12 years), maternal parity, birth weight, maternal RCT group assignment, and sex.    Dose-response relationship between composite feeding scores and full-scale IQ, verbal comprehension, working memory, and information processing speed, after adjusting for covariates.  Those who regularly consumed iron-fortified or iron-rich foods between 6-24 months had higher full-scale IQ, non-verbal IQ, working memory, processing speed, and verbal comprehension scores than those who did not consume such foods. In those who consumed such foods, the timing of food introduction between 6-24 months was not associated with cognition.    Those who initiated cows/goats milk consumption after 13 months had lower full-scale IQ scores than those who initiated milk consumption between 10 – 12 months, but higher working memory scores than those who initiated milk consumption before 6 months.  Those who initiated high protein food intake by 6 months had lower full-scale IQ and non-verbal IQ scores than those who initiated high protein food intake between 7-9 month. |
| **Iron (n = 3)** | | | | | | |
| Algarin et al. (2013) | N = 132 participants who took part in a RCT as infants and had cognitive data at 10 years (106 males, 26 females).  Chile.  Low SES.  Recruited infants with and without IDA during at 6, 12, or 18 months. Haemoglobin concentrations were within the normal range at the end of trial in those with IDA at the start of the trial. 3 participants had IDA at the 10-year follow-up and were excluded from the analysis. | All infants, irrespective of iron status, received oral iron (15-30mg p/d, depending on age) for 1 year (6-month-old infants) or a minimum of 6 months (12- and 18-month-old infants). | Administered at 10 years:  Go/No-Go task (inhibitory control). | N/A | Measured at 10 years:  Event Related Potentials. | Covariates: SES, sex, and 10-year iron status.  Despite successful iron treatment, participants who had IDA during infancy had poorer inhibitory control, a longer latency to N2 peak, and a smaller P300 amplitude during adolescence than participants who did not have IDA during infancy. |
| Lozoff et al. (2000) | N = 167 participants who took part in a RCT as infants (aged 12-23 months) and had cognitive data between 11-14 years.  Low SES.  Costa Rica.  Baseline iron status varied from iron-sufficient to marked IDA. None of the infants were anaemic post-iron treatment, but some remained ID.  Comparisons were made between the good iron status group (those who were iron-sufficient before and/or after iron treatment) and chronic-ID group (those who were still ID after iron treatment).  2% of participants were ID at follow-up. | 3-month, double-blind, BS, RCT.  Iron-sufficient infants received a daily placebo for 3 months, whereas those with ID or IDA received 6mg iron p/kg p/d for 3 months. | Administered between 11-14 years:  WISC-R (verbal, performance, and full-scale IQ); Bender Visual-Motor Gestalt Test (perception); Central/  Incidental Serial Recall Test (selective attention); Attentional Capacity Test (auditory attention); Underlining Test (information processing speed); K-ABC Spatial Memory (spatial memory); Cognitive Abilities Tests (general cognitive abilities). | Measured between 11-14 years:  Academic grades (school records and teacher questionnaire); Wide Range Achievement Test-R (reading and arithmetic skills); Directed Writing Task (literacy skills) | N/A | Covariates: age, sex, maternal IQ or education, and HOME scores.  Arithmetic, writing, selective attention, and spatial memory scores between 11-14 years were poorer in those whose ID remained after iron treatment than those whose ID resolved after iron treatment or those who were iron sufficient during infancy. Differences in verbal and full-scale IQ scores were no longer significant after controlling for confounding factors. |
| Lukowski et al. (2010) | N = 114 participants who took part in a RCT during infancy and had cognitive data at 19 years.  See Lozoff et al. (2000) | See Lozoff et al. (2000) | Administered at 19 years:  TMT-A (information processing speed); TMT-B (mental flexibility/shifting); Cambridge Neuro-psychological Test Automated Battery (Spatial Working Memory subtest [spatial working memory]; Intra/Extra-dimensional Shift test [mental flexibility/shifting], Stockings of Cambridge [planning], Pattern Recognition Memory test, Delayed Match to Sample test, and Spatial Recognition Memory test [visual memory]; RT test and Rapid Visual Processing test [information processing speed]). | N/A | N/A | Covariates: age, sex, mothers IQ or education, and HOME scores.  Mental flexibility/shifting and planning abilities at 19 years were poorer in those with residual ID during infancy than those with normal iron levels before and/or after iron treatment. |

1 = dietary patterns were also obtained at 4 and 7 years. 2 = dietary patterns were also obtained at 4, 7, and 8.5 years.

EAT = Eating Assessment in Toddlers, BMI = body mass index, BS = between-subjects, CPAL = continuous paired associative learning, FFQ = food frequency questionnaire, IDA = iron-deficient anaemic, ID = iron deficiency, IQ = intelligence quotient, MRI = magnetic resonance imaging, MVM = multivitamin and mineral, PCA = Principal Component Analysis, RCT = randomised controlled trial, RT = reaction time, SES = socioeconomic status, TMT = trail-making test, WASI = Wechsler Abbreviated Scale of Intelligence, WCST = Wisconsin Card Sorting Test, WISC = Wechsler Intelligence Scale for Children, WHO = World Health Organisation.

**Table S11.** Prospective studies examining the effect of diet during early adolescence on outcomes during late adolescence.

| **Author**  **(year)** | **Sample characteristics** | **Dietary patterns or intervention** | **Outcome measures** | | | **Results** |
| --- | --- | --- | --- | --- | --- | --- |
|  |  |  | **Cognitive measures** | **Academic measures** | **Biochemical and neurological measures** |  |
| **Fish intake (n = 2)** | | | | | | |
| Åberg et al. (2009) | N = 3972 males with dietary data at 15 years and cognitive data at 18 years.  Sweden.  Mixed SES. | Measured at baseline (15 years):  Self-reported frequency of fish intake (more than once p/w, once p/w, or less than once p/w). | Measured at 3-year follow-up (18 years):  Intelligence tests completed during military service conscription at 18 years of age (combined IQ, verbal abilities, and visuospatial abilities). | N/A | N/A | Covariates obtained at 15 years: BMI, residence area, foreign descent, type of housing, having a dishwasher, parental education, and frequency of physical exercise.    Strong positive association between frequency of fish intake p/w at 15 years and combined IQ, verbal abilities, and visuospatial abilities at 17 years, after adjusting for covariates.  Compared to those who consumed fish less than once p/w, those who consumed fish more than once p/w at 15 years had significantly higher combined IQ scores and better verbal and visuospatial abilities. |
| Kim et al. (2010) | N = 9448 participants with dietary data at 15 years and academic grades at 16 years (4674 males, 4774 females).  Sweden.  Mixed SES. | Measured at baseline (15 years):  Self-reported frequency of fish intake (more than once p/w, once p/w, or less than once p/w). | N/A | Measured at 1-year follow-up (16 years):  Academic grade (standardised school tests. Sum of grades in 16 subjects). | N/A | Covariates obtained at 15 years: BMI, residence area, foreign descent, sex, type of housing, having a dishwasher, parental education, and frequency of physical exercise.    Compared to those who consumed fish less than once p/w. those who consumed fish once p/w at 15 years had higher average grades at 16 years. A larger difference in average grades was observed between those who consumed fish less than once p/w and more than once p/w at 15 years. |
| **General dietary quality (n = 7)** | | | | | | |
| Dubuc et al. (2019) | N = 187 participants with dietary, cognitive, and academic data at 12-13 years and 15-16 years (71 males, 116 females).  Canada.  1 school for elite students.  ASAP study. | Measured at baseline (12-13 years) and 3-year follow-up (15-16 years):  Self-reported dietary intake (number of meals p/d, servings of fruit and vegetables p/d, and breakfast intake on weekdays and weekends). | Administered at baseline and 3-year follow-up:  Arrow Flankers test (selective attention and inhibition) and n-back test (working memory). | Measured at baseline and 3-year follow-up:  Academic grades (standardised tests of science, mathematics, and language. Average grade for 9 subjects). | N/A | Covariates: age, ethnicity, pubertal status, and SES.  In females, changes in lifestyle habits (including diet, sleep, physical activity levels, screentime, social media, and video games) did not predict the change in academic grades after 3 years. The consumption of daily meals at baseline significantly predicted the change in attention and working memory after 3 years. Screen time and social media use at baseline were also independent predictors.    In males, an increase in daily servings of fruits and vegetables from baseline to follow-up was associated with an increase in average grades. |
| Faught et al. (2017) | N = 4253 participants with dietary data at 10-11 years and academic grades at 12-13 years (2007 males, 2246 females).  Mixed SES.  Canada.  CLASS study. | Administered at baseline (10-11 years):  Harvard Food Frequency Questionnaire (adherence to Eating Well with Canada’s Food Guide on previous day, including meeting recommendations for daily servings of fruit and vegetables [6 servings], grain products [6 servings], milk and alternatives [3-4 servings], meat and alternatives [2 servings], and free sugars and saturated fats [<10% of total energy intake]). | N/A | Measured at 1 year follow-up (12-13 years):  Academic grades (standardised tests of reading, writing, and mathematics. Categorised as meeting expectations or not meeting expectations). | N/A | Covariates: sex, parental education, area of residence, energy intake, and household income.  After adjusting for covariates, and adherence to other lifestyle behaviour recommendations (physical activity levels, sleep habits, screen time, and body weight), meeting recommendations for milk or meat at baseline was associated with an increased likelihood of meeting mathematics expectations 1 year later,  whilst meeting recommendations for all food groups at baseline (other than fruits and vegetables) was independently associated with an increased likelihood of meeting reading expectations 1 year later. Meeting recommendations for meat, free sugars, sleep, or screen time at baseline was independently associated with an increased likelihood of meeting writing expectations 1 year later. |
| Faught et al. (2019) | N = 11,016 (5288 males, 5728 females).  Mixed SES.  Canada.  65 schools.  COMPASS study. | Measured at baseline (13-18 years) and 1 year follow-up (14-19 years):  Self-reported dietary intake (Adherence to Eating Well with Canada’s Food Guide on previous day, including meeting recommendations for daily servings of fruit and vegetables [6 servings], grain products [6 servings], milk and alternatives [3-4 servings], and meat and alternatives [2 servings]). | N/A | Measured at baseline and 1 year follow-up:  Academic grades (self-reported mathematics and English grades). | N/A | Covariates: sex, age, ethnicity, available spending money, BMI, baseline academic grades, and physical activity levels.  Compared to those who did not meet recommendations at baseline or follow-up, those who met recommendations for meat at either or both time points, milk at both time points, or fruit and vegetables at follow-up, had an increased likelihood of obtaining better mathematics grades at follow-up.  Those who met meat recommendations at follow or both time points, or fruit and vegetables recommendations at follow-up only, had higher English grades at follow-up than those who did not meet recommendations at baseline or follow-up. |
| Mou et al. (2023) | N = 2326 participants with dietary data at 8 years and cognitive data at 13 years (1147 males, 1179 females).  Mostly middle SES.  Netherlands.  Generation R Study. | Administered at baseline (8 years):  221-item FFQ completed by primary caregiver (Diet-Quality Score based on adherence to Dutch dietary guidelines for 10 food groups. PCA identified 4 dietary patterns: *snacks, potatoes, and processed foods*, *fish, vegetables, and fruit*, *whole grains, soft fats, and dairy,* and *meat replacements, legumes, and nuts*. | Administered at 5-year follow-up (13 years):  WISC-V (full-scale IQ). | N/A | Measured at 2-year follow-up (10 years):  Brain morphology (MRI scan). | Covariates: sex, age at neuroimaging assessment, ethnicity, energy intake at 1 and 8 years, and BMI at 10 years. Covariates obtained during pregnancy: maternal education, psychopathology symptoms, alcohol use, tobacco use, folic acid supplement use, diet quality, and household income.  Higher adherence to the *whole grains, soft fats, and dairy* dietary pattern at 8 years was positively associated with total brain and cerebral gray matter volumes at 10 years. No association between this dietary pattern at 8 years and hippocampal or amygdala volume at 10 years.  The association between the *whole grains, soft fats, and dairy* dietary pattern at 8 years and full-scale IQ at 13 years was mediated by total brain and cerebral grey matter volumes at 10 years. |
| Nigg and Amato (2015) | N = 334 (150 males, 184 females).  Hawaii.  Recruited 3 age groups from the Fun 5 after-school programme. | Measured at baseline (9-12 years) and 5-year follow-up (14-17 years):  Self-reported daily servings of fruit and vegetable. | N/A | Measured at 5-year follow-up:  Academic grades (self-reported average academic grade). | N/A | Covariates: sex and ethnicity.  Fruit and vegetable intake at baseline negatively predicted academic grades 5 years later. |
| Nyaradi et al. (2014) | N = 602 participants with dietary data at 14 years and cognitive data at 17 years.  Australia.  Mixed SES.  Raine study cohort (Generation 2). | Administered at baseline (14 years):  212-item FFQ completed by primary caregiver (factor analysis identified 2 dietary patterns: *healthy* [high in fruits, vegetables, whole grains, legumes, and fish] and *Western* [high in soft drinks, fried/refined food, takeaways, and red/processed meat]). | Administered at 3-year follow-up (17 years):  CogState (Detection test [information processing speed], Identification test [attention], One Card Learning test [visual memory], CPAL test [spatial memory]), and Groton Maze Learning test [spatial memory]. | N/A | N/A | Covariates: sex, maternal education at 8 years, and family income, presence of biological father in family, family functioning, and total energy intake at 14 years.  A *Western* dietary pattern was associated with slower information processing speed and poorer delayed spatial memory (Groton Maze Learning test).  Lower intake of green leafy vegetables or fruit was associated with slower information processing speed and poorer spatial memory, respectively. Higher intake of fried potatoes or crisps was associated with slower information processing speed. Higher intake of fried potatoes was also associated with poorer attention and spatial memory (CPAL). Higher intake of crisps and red meat was associated with poorer visual memory. |
| Purtell and Gershoff (2015) | N = 8544 participants with dietary data at 10-11 years and academic grades at 13-14 years (4357 males, 4187 females).  USA.  Mixed SES.  ECLS-K cohort. | Measured at baseline (10-11 years):  Youth Risk Behaviour Surveillance System (self-reported fast-food intake throughout the previous week: daily, 4-6 times, 1-3 times, or none). | N/A | Measured at 3-year follow-up (13-14 years):  Academic grades (standardised tests of literacy, mathematics, and science). | N/A | Covariates obtained at 10-11 years: sex, ethnicity, parent-reported overall health, academic grades, intake of fruit, vegetables, 100% juice beverages, and soft drinks, household income-to-needs ratio, combined parental education, maternal employment, physical activity level, hours of TV, regular bedtime, area of residence, school poverty level, and household food insecurity.  Those who reported consuming fast food on a daily basis at 10-11 years had poorer mathematics, literacy, and science grades at 13-14 years than those who reported consuming no fast food. Those who reported consuming any amount of fast food at 10-11 years made smaller improvements in mathematics grades by 13-14 years. |
| **Mediterranean diet (n = 1)** | | | | | | |
| Hayek et al. (2021) | N = 563 participants with dietary and academic data between 15-18 years (283 males, 280 females).  Lebanon.  Mixed SES.  7 schools (4 private and 3 public). | Administered at baseline (15-18 years):  64-item self-report FFQ (adherence to Mediterranean diet – KIDMED index). | N/A | Measured at 6- and 12-month follow-up:  Academic grades (self-reported average academic grade). | N/A | Covariates: parenting style, age, sex, school type, family structure, religion, and parental education.  An increase in the adherence to the Mediterranean diet was significantly associated with an increase in academic achievement, irrespective of time (6 and 12 months later). |
| **School breakfast programs (n = 2)** | | | | | | |
| Murphy et al. (1998) | N = 133 (58 males, 75 females).  Mean age = 10.3 years (age range = 8-14 years).  USA.  3 schools.  Low SES (>70% of students were eligible for free or reduced-price meals). | Measured at baseline (before SBPs were implemented) and 4-month follow-up:  Number of times within a week that school breakfast was consumed (categorised as ‘often’, ‘sometimes’, or ‘rarely’. The change in participation after 4 months was categorised as ‘increased’, ‘decreased’, or ‘remained the same’). | N/A | Measured at baseline and 4-month follow-up:  Academic grades (mathematics, science, social studies, and reading). | N/A | Those who increased their participation in the SBPs after 4 months had significantly larger increases in mathematic grades than those whose participation remained the same or decreased. |
| Powell, Grantham-McGregor et al. (1983) | N = 115.  Age range = 12-13 years.  Jamaica.  1 rural school (3/10 classes selected. Lowest scholastic ability, attendance rates, and weight).  Low SES.  Participants were undernourished (according to weight-for-age). | One class received 100ml milk with a slice of cake or meat-filled pastry p/d for 3 months. The other classes received either a daily syrup drink or nothing. Breakfast was served at 9am. | N/A | Administered at baseline (1-month and immediately prior to SBP) and at 3-month follow-up:  Wide Range Achievement Test (arithmetic, spelling, and reading). | N/A | Covariates: sex, age, and treatment group.  The provision of a breakfast meal at school for 3 months was associated with an increase in arithmetic scores compared to those who received a syrup drink or nothing for breakfast. |

BMI = body mass index, CPAL = continuous paired associative learning, FFQ = food frequency questionnaire, IQ = intelligence quotient, MRI = magnetic resonance imaging, p/w = per week, PCA = principal component analysis, SES = socioeconomic status, SBP = school breakfast programme, WISC = Wechsler Intelligence Scale for Children.

Åberg, M. A. I., N. Åberg, J. Brisman, R. Sundberg, A. Winkvist and K. Torén (2009). "Fish intake of Swedish male adolescents is a predictor of cognitive performance." ACTA PAEDIATRICA **98**(3): 555–560.

Algarín, C., C. A. Nelson, P. Peirano, A. Westerlund, S. Reyes and B. Lozoff (2013). "Iron‐deficiency anemia in infancy and poorer cognitive inhibitory control at age 10 years." Developmental medicine and child neurology **55**(5): 453–458.

Bruner, A. B., A. Joffe, A. K. Duggan, J. F. Casella and J. Brandt (1996). "Randomised study of cognitive effects of iron supplementation in non-anaemic iron-deficient adolescent girls." The Lancet (British edition) **348**(9033): 992–996.

Buzina-Suboticanec K, B. R., Stavljenic A, Tadinac-Babic M, Juhovic-Markus V. (1998). "Effects of iron supplementation on iron nutrition status and cognitive functions in children." Food and nutrition bulletin **19**(4): 298–306.

Chellappa, A. R., & Karunanidhi, S. (2012). "Effect of iron and zinc supplementation on cognitive functions of female adolescents in Chennai, India." Paper presented at the International Conference on Nutrition and Food Sciences IPCBEE.

Chung, Y.-C. M. D. P. D., C.-H. M. D. Park, H.-K. B. N. Kwon, Y.-M. M. S. Park, Y. S. P. D. Kim, J.-K. M. D. P. D. Doo, D.-H. P. D. Shin, E.-S. M. S. Jung, M.-R. M. S. Oh and S. W. M. D. P. D. Chae (2012). "Improved cognitive performance following supplementation with a mixed-grain diet in high school students: A randomized controlled trial." Nutrition (Burbank, Los Angeles County, Calif.) **28**(2): 165–172.

Cueto, S. and M. Chinen (2008). "Educational impact of a school breakfast programme in rural Peru." International journal of educational development **28**(2): 132–148.

Devaki, P. B., Chandra, R. K., & Geisser, P. (2009). "Effects of oral iron(III) hydroxide polymaltose complex supplementation on hemoglobin increase, cognitive function, affective behavior and scholastic performance of adolescents with varying iron status: a single centre prospective placebo controlled study." Arzneimittel-Forschung **59**(6): 303–310.

Dubuc, M.-M., M. Aubertin-Leheudre and A. D. Karelis (2019). "Lifestyle habits predict academic performance in high school students: The adolescent student academic performance longitudinal study (ASAP)." International journal of environmental research and public health **17**(1): 243.

Faught, E. L., J. P. Ekwaru, D. Gleddie, K. E. Storey, M. Asbridge and P. J. Veugelers (2017). "The combined impact of diet, physical activity, sleep and screen time on academic achievement: A prospective study of elementary school students in Nova Scotia, Canada." The international journal of behavioral nutrition and physical activity **14**(1): 29–29.

Faught, E. L., W. Qian, V. L. Carson, K. E. Storey, G. Faulkner, P. J. Veugelers and S. T. Leatherdale (2019). "The longitudinal impact of diet, physical activity, sleep, and screen time on Canadian adolescents' academic achievement: An analysis from the COMPASS study." Preventive medicine **125**: 24–31.

Feinstein, L., R. Sabates, A. Sorhaindo, I. Rogers, D. Herrick, K. Northstone and P. Emmett (2008). "Dietary patterns related to attainment in school: the importance of early eating patterns." Journal of epidemiology and community health (1979) **62**(8): 734–739.

Golley, R. K., L. G. Smithers, M. N. Mittinty, P. Emmett, K. Northstone and J. W. Lynch (2013). "Diet quality of UK infants is associated with dietary, adiposity, cardiovascular, and cognitive outcomes measured at 7-8 years of age." The Journal of nutrition **143**(10): 1611–1617.

Gordon, R. C., M. C. Rose, S. A. Skeaff, A. R. Gray, K. M. D. Morgan and T. Ruffman (2009). "Iodine supplementation improves cognition in mildly iodine-deficient children." The American journal of clinical nutrition **90**(5): 1264–1271.

Grung, B., A. M. Sandvik, K. Hjelle, L. Dahl, L. Frøyland, I. Nygård and A. L. Hansen (2017). "Linking vitamin D status, executive functioning and self‐perceived mental health in adolescents through multivariate analysis: A randomized double‐blind placebo control trial." Scandinavian journal of psychology **58**(2): 123–130.

Handeland, K., J. Oyen, S. Skotheim, I. E. Graff, V. Baste, M. Kjellevold, L. Froyland, O. Lie, L. Dahl and K. M. Stormark (2017). "Fatty fish intake and attention performance in 14-15 year old adolescents: FINS-TEENS - A randomized controlled trial." Nutrition journal **16**(1): 64–64.

Handeland, K., S. Skotheim, V. Baste, I. E. Graff, L. Froyland, O. Lie, M. Kjellevold, M. W. Markhus, K. M. Stormark, J. Oyen and L. Dahl (2018). "The effects of fatty fish intake on adolescents' nutritional status and associations with attention performance: Results from the FINS-TEENS randomized controlled trial." Nutrition journal **17**(1): 30–30.

Haskell, C. F., A. B. Scholey, P. A. Jackson, J. M. Elliott, M. A. Defeyter, J. Greer, B. C. Robertson, T. Buchanan, B. Tiplady and D. O. Kennedy (2008). "Cognitive and mood effects in healthy children during 12 weeks' supplementation with multi-vitamin/minerals." British journal of nutrition **100**(5): 1086–1096.

Hayek, J., H. de Vries, M. Tueni, N. Lahoud, B. Winkens and F. Schneider (2021). "Increased adherence to the mediterranean diet and higher efficacy beliefs are associated with better academic achievement: A longitudinal study of high school adolescents in lebanon." International journal of environmental research and public health **18**(13): 6928.

Huda, S. N., S. M. Grantham-McGregor and A. Tomkins (2001). "Cognitive and Motor Functions of Iodine-Deficient but Euthyroid Children In Bangladesh Do not Benefit from Iodized Poppy Seed Oil (Lipiodol)." The Journal of nutrition **131**(1): 72–77.

Isa, Z. M. D., I. Z. Alias, K. A. Kadir and O. Ali (2000). "Effect of iodized oil supplementation on thyroid hormone levels and mental performance among Orang Asli schoolchildren and pregnant mothers in an endemic goitre area in Peninsular Malaysia." Asia Pacific journal of clinical nutrition **9**(4): 274–281.

Kalaichelvi, D. S., J.N. (2021). "Effectiveness of nutritional intervention in improving intelligence among adolescent girls." International Journal of Current Research **13**(11): 19560–19564.

Karkada, S., S. Upadhya, S. Upadhya and G. Bhat (2019). "Beneficial Effects of ragi (Finger Millet) on Hematological Parameters, Body Mass Index, and Scholastic Performance among Anemic Adolescent High-School Girls (AHSG)." Comprehensive child and adolescent nursing **42**(2): 141–150.

Kashyap, P., & Gopaldas, T. (1987). "Impact of hematinic supplementation on cognitive function in underprivileged school girls (8–15 yrs of age)." Nutrition Research **7**(11): 1117–1126.

Kennedy, D. O., P. A. Jackson, J. M. Elliott, A. B. Scholey, B. C. Robertson, J. Greer, B. Tiplady, T. Buchanan and C. F. Haskell (2009). "Cognitive and mood effects of 8 weeks' supplementation with 400 mg or 1000 mg of the omega-3 essential fatty acid docosahexaenoic acid (DHA) in healthy children aged 10-12 years." Nutritional neuroscience **12**(2): 48–56.

Kim, J. L., A. Winkvist, M. A. Åberg, N. Åberg, R. Sundberg, K. Torén and J. Brisman (2010). "Fish consumption and school grades in Swedish adolescents: a study of the large general population." ACTA PAEDIATRICA **99**(1): 72–77.

Kirby, A., A. Woodward, S. Jackson, Y. Wang and M. A. Crawford (2010). "A double-blind, placebo-controlled study investigating the effects of omega-3 supplementation in children aged 8–10 years from a mainstream school population." Research in developmental disabilities **31**(3): 718–730.

Lambert, A. K., Knaggs, K., Scragg, R., Metcalf, P., & Schaaf, D. (2002). "Effects of iron treatment on cognitive performance and working memory in non-anaemic, iron-deficient girls." New Zealand Journal of Psychology **31**(1): 19 – 28.

Lozoff, B., E. Jimenez, J. Hagen, E. Mollen and A. W. Wolf (2000). "Poorer behavioral and developmental outcome more than 10 years after treatment for iron deficiency in infancy." Pediatrics **105**(4): E51.

Lukowski, A. F., M. Koss, M. J. Burden, J. Jonides, C. A. Nelson, N. Kaciroti, E. Jimenez and B. Lozoff (2010). "Iron deficiency in infancy and neurocognitive functioning at 19 years: evidence of long-term deficits in executive function and recognition memory." Nutritional neuroscience **13**(2): 54–70.

Lynn, R. and E. P. Harland (1998). "A positive effect of iron supplementation on the IQS of iron deficient children." Personality and individual differences **24**(6): 883–885.

McNamara, R. K., J. Able, R. Jandacek, T. Rider, P. Tso, J. C. Eliassen, D. Alfieri, W. Weber, K. Jarvis, M. P. DelBello, S. M. Strakowski and C. M. Adler (2010). "Docosahexaenoic acid supplementation increases prefrontal cortex activation during sustained attention in healthy boys: a placebo-controlled, dose-ranging, functional magnetic resonance imaging study." The American journal of clinical nutrition **91**(4): 1060–1067.

Mou, Y., E. Blok, M. Barroso, P. W. Jansen, T. White and T. Voortman (2023). "Dietary patterns, brain morphology and cognitive performance in children: Results from a prospective population-based study." European journal of epidemiology **38**(6): 669–687.

Murphy, J. M., M. E. Pagano, J. Nachmani, P. Sperling, S. Kane and R. E. Kleinman (1998). "The Relationship of School Breakfast to Psychosocial and Academic Functioning: Cross-sectional and Longitudinal Observations in an Inner-city School Sample." Archives of pediatrics & adolescent medicine **152**(9): 899–907.

Murphy, S., G. F. Moore, K. Tapper, R. Lynch, R. Clarke, L. Raisanen, C. Desousa and L. Moore (2011). "Free healthy breakfasts in primary schools: a cluster randomised controlled trial of a policy intervention in Wales, UK." Public health nutrition **14**(2): 219–226.

Nidich, S. I., P. Morehead, R. J. Nidich, D. Sands and H. Sharma (1993). "The effect of the Maharishi Student Rasayana food supplement on non-verbal intelligence." Personality and individual differences **15**(5): 599–602.

Nigg, C. R. and K. Amato (2015). "The Influence of Health Behaviors During Childhood on Adolescent Health Behaviors, Health Indicators, and Academic Outcomes Among Participants from Hawaii." International journal of behavioral medicine **22**(4): 452–460.

Northstone, K., C. Joinson, P. Emmett, A. Ness and T. Paus (2012). "Are dietary patterns in childhood associated with IQ at 8 years of age? A population-based cohort study." Journal of epidemiology and community health (1979) **66**(7): 624–628.

Nyaradi, A., J. K. Foster, S. Hickling, J. Li, G. L. Ambrosini, A. Jacques and W. H. Oddy (2014). "Prospective associations between dietary patterns and cognitive performance during adolescence." Journal of child psychology and psychiatry **55**(9): 1017–1024.

Nyaradi, A., J. Li, J. K. Foster, S. Hickling, A. Jacques, T. A. O'Sullivan and W. H. Oddy (2016). "Good-quality diet in the early years may have a positive effect on academic achievement." ACTA PAEDIATRICA **105**(5): e209–e218.

Nyaradi, A., J. Li, S. Hickling, A. J. O. Whitehouse, J. K. Foster and W. H. Oddy (2013). "Diet in the early years of life influences cognitive outcomes at 10 years: a prospective cohort study." ACTA PAEDIATRICA **102**(12): 1165–1173.

Nyaradi, A., W. H. Oddy, S. Hickling, J. Li and J. K. Foster (2015). "The relationship between nutrition in infancy and cognitive performance during adolescence." Frontiers in nutrition (Lausanne) **2**: 2––.

O’Connor, P. J., X. Chen, L. M. Coheley, M. Yu, E. M. Laing, A. Oshri, A. Marand, J. Lance, K. Kealey and R. D. Lewis (2022). "The effects of 9 months of formulated whole-egg or milk powder food products as meal or snack replacements on executive function in preadolescents: A randomized, placebo-controlled trial." The American journal of clinical nutrition **116**(6): 1663–1671.

Perlman, A. I. M. D. M. P. H. F., J. P. Worobey, J. P. R. D. F. O'Sullivan Maillet, R. P. R. D. F. Touger-Decker, D. L. M. S. Hom and J. K. P. Smith (2010). "Multivitamin/Mineral Supplementation Does Not Affect Standardized Assessment of Academic Performance in Elementary School Children." Journal of the American Dietetic Association **110**(7): 1089–1093.

Petrova, D., M. A. Bernabeu Litrán, E. García-Mármol, M. Rodríguez-Rodríguez, B. Cueto-Martín, E. López-Huertas, A. Catena and J. Fonollá (2019). "Еffects of fortified milk on cognitive abilities in school-aged children: results from a randomized-controlled trial." European journal of nutrition **58**(5): 1863–1872.

Pinar-Martí, A., F. Gignac, S. Fernández-Barrés, D. Romaguera, A. Sala-Vila, I. Lázaro, O. T. Ranzani, C. Persavento, A. Delgado, A. Carol, J. Torrent, J. Gonzalez, E. Roso, J. Barrera-Gómez, M. López-Vicente, O. Boucher, M. Nieuwenhuijsen, M. C. Turner, M. Burgaleta, J. Canals, V. Arija, X. Basagaña, E. Ros, J. Salas-Salvadó, J. Sunyer and J. Julvez (2023). "Effect of walnut consumption on neuropsychological development in healthy adolescents: a multi-school randomised controlled trial." EClinicalMedicine **59**: 101954–101954.

Pollitt, E. (1997). "Iron Deficiency and Educational Deficiency." Nutrition reviews **55**(4): 133–141.

Pollitt, E., Hathiral, P., Kotchabhakdi, N. J., Missell, L., & Valyasevi, A. (1989). "Iron deficiency and educational achievement in Thailand." The American journal of clinical nutrition **50**(3): 687–697.

Pollitt, E., A. G. Soemantri, F. Yunis and N. S. Scrimshaw (1985). "Cognitive effects of iron-deficiency anaemia." Lancet **1**(8421): 158.

Portillo-Reyes, V., M. Pérez-García, Y. Loya-Méndez and A. E. Puente (2014). "Clinical significance of neuropsychological improvement after supplementation with omega-3 in 8–12 years old malnourished Mexican children: A randomized, double-blind, placebo and treatment clinical trial." Research in developmental disabilities **35**(4): 861–870.

Powell, C., S. Grantham-McGregor and M. Elston (1983). "An evaluation of giving the Jamaican government school meal to a class of children." Hum Nutr Clin Nutr **37**(5): 381–388.

Purtell, K. M. and E. T. Gershoff (2015). "Fast Food Consumption and Academic Growth in Late Childhood." Clinical pediatrics **54**(9): 871–877.

Rezaeian, A., M. Ghayour-Mobarhan, S. R. Mazloum, M. Yavari and S.-A. Jafari (2014). "Effects of iron supplementation twice a week on attention score and haematologic measures in female high school students." Singapore medical journal **55**(11): 587–592.

Schoenthaler, S. J., S. P. Amos, H. J. Eysenck, E. Peritz and J. Yudkin (1991). "Controlled trial of vitamin-mineral supplementation: Effects of intelligence and performance." Personality and individual differences **12**(4): 351–362.

Scott, S. P., L. E. Murray-Kolb, M. J. Wenger, S. A. Udipi, P. S. Ghugre, E. Boy and J. D. Haas (2018). "Cognitive Performance in Indian School-Going Adolescents Is Positively Affected by Consumption of Iron-Biofortified Pearl Millet: A 6-Month Randomized Controlled Efficacy Trial." The Journal of nutrition **148**(9): 1462–1471.

Sen, A. and S. J. Kanani (2009). "Impact of iron-folic acid supplementation on cognitive abilities of school girls in Vadodara." Indian pediatrics **46**(2): 137–143.

Shemilt, I., I. Harvey, L. Shepstone, L. Swift, R. Reading, M. Mugford, P. Belderson, N. Norris, J. Thoburn and J. Robinson (2004). "A national evaluation of school breakfast clubs: evidence from a cluster randomized controlled trial and an observational analysis." Child : care, health & development **30**(5): 413–427.

Smithers, L. G., R. K. Golley, M. N. Mittinty, L. Brazionis, K. Northstone, P. Emmett and J. W. Lynch (2012). "Dietary patterns at 6, 15 and 24 months of age are associated with IQ at 8 years of age." European journal of epidemiology **27**(7): 525–535.

Smithers, L. G., R. K. Golley, M. N. Mittinty, L. Brazionis, K. Northstone, P. Emmett and J. W. Lynch (2013). "Do Dietary Trajectories between Infancy and Toddlerhood Influence IQ in Childhood and Adolescence? Results from a Prospective Birth Cohort Study." PloS one **8**(3): e58904–e58904.

Snowden, W. (1997). "Evidence from an analysis of 2000 errors and omissions made in IQ tests by a small sample of schoolchildren, undergoing vitamin and mineral supplementation, that speed of processing is an important factor in IQ performance." Personality and individual differences **22**(1): 131–134.

Soemantri, A. G. (1989). "Preliminary findings on iron supplementation and learning achievement of rural Indonesian children." The American journal of clinical nutrition **50**(3): 698–702.

Soemantri, A. G., Pollitt, E. & Kim, I (1985). "Iron deficiency anemia and educational achievement,." The American Journal of Clinical Nutrition, **42**(6): 1221–1228,.

Sørensen, L. B., C. T. Damsgaard, S.-M. Dalskov, R. A. Petersen, N. Egelund, C. B. Dyssegaard, K. D. Stark, R. Andersen, I. Tetens, A. Astrup, K. F. Michaelsen and L. Lauritzen (2015). "Diet-induced changes in iron and n-3 fatty acid status and associations with cognitive performance in 8–11-year-old Danish children: secondary analyses of the Optimal Well-Being, Development and Health for Danish Children through a Healthy New Nordic Diet School Meal Study." British journal of nutrition **114**(10): 1623–1637.

Sørensen, L. B., C. T. Damsgaard, R. A. Petersen, S. M. Dalskov, M. F. Hjorth, C. B. Dyssegaard, N. Egelund, I. Tetens, A. Astrup, L. Lauritzen and K. F. Michaelsen (2016). "Differences in the effects of school meals on children’s cognitive performance according to gender, household education and baseline reading skills." European journal of clinical nutrition **70**(10): 1155–1161.

Sørensen, L. B., C. B. Dyssegaard, C. T. Damsgaard, R. A. Petersen, S.-M. Dalskov, M. F. Hjorth, R. Andersen, I. Tetens, C. Ritz, A. Astrup, L. Lauritzen, K. F. Michaelsen and N. Egelund (2015). "The effects of Nordic school meals on concentration and school performance in 8- to 11-year-old children in the OPUS School Meal Study: a cluster-randomised, controlled, cross-over trial." British journal of nutrition **113**(8): 1280–1291.

Southon, S., A. J. A. Wright, P. M. Finglas, A. L. Bailey, J. M. Loughridge and A. D. Walker (1994). "Dietary intake and micronutrient status of adolescents: effect of vitamin and trace element supplementation on indices of status and performance in tests of verbal and non-verbal intelligence." British journal of nutrition **71**(6): 897–918.

Tefagh, S., R. Mokaberinejad, M. Shakiba, M. Jafari, M. Salehi, M. Khayatkashani and N. Shakeri (2022). "Effect of Ustukhuddus Alavi, a multi-herbal product, on the cognitive performance of adolescent female students." Journal of ethnopharmacology **288**(NA): 114971–114971.

Teisen, M. N., S. Vuholm, J. Niclasen, J. J. Aristizabal-Henao, K. D. Stark, S. S. Geertsen, C. T. Damsgaard and L. Lauritzen (2020). "Effects of oily fish intake on cognitive and socioemotional function in healthy 8–9-year-old children: the FiSK Junior randomized trial." The American journal of clinical nutrition **112**(1): 74–83.

van der Wurff, I. S. M., C. von Schacky, T. Bergeland, R. Leontjevas, M. P. Zeegers, J. Jolles, P. A. Kirschner and R. H. M. de Groot (2019). "Effect of 1 year krill oil supplementation on cognitive achievement of dutch adolescents: A double-blind randomized controlled trial." Nutrients **11**(6): 1230.

van der Wurff, I. S. M., C. von Schacky, T. Bergeland, M. P. Zeegers, P. A. Kirschner and R. H. M. de Groot (2023). "Krill oil supplementation's effect on school grades in typically developing adolescents." Prostaglandins, leukotrienes and essential fatty acids **191**: 102553–102553.

Wang, X., Z. Hui, X. Dai, P. D. Terry, Y. Zhang, M. Ma, M. Wang, F. Deng, W. Gu, S. Lei, L. Li, M. Ma and B. Zhang (2017). "Micronutrient‐fortified milk and academic performance among Chinese middle school students: A cluster‐randomized controlled trial." Nutrients **9**(3): 226.

Zhu, Z., Y. Cheng, Q. Qi, Y. Lu, S. Ma, S. Li, H. Li, M. Elhoumed, S. Tsegaye, W. W. Fawzi, C. R. Sudfeld, H. Yan, M. J. Dibley and L. Zeng (2020). "Association of infant and young child feeding practices with cognitive development at 10-12 years: A birth cohort in rural Western China." British journal of nutrition **123**(7): 768–779.

Zimmermann, M. B., K. Connolly, M. Bozo, J. Bridson, F. Rohner and L. Grimci (2006). "Iodine supplementation improves cognition in iodine-deficient schoolchildren in Albania: a randomized, controlled, double-blind study." The American journal of clinical nutrition **83**(1): 108–114.
